# Synthesis and human carbonic anhydrase I, II, VA, and XII inhibition with novel amino acid–sulphonamide conjugates

**DOI:** 10.1080/14756366.2019.1710503

**Published:** 2020-01-08

**Authors:** Hasan Küçükbay, Nesrin Buğday, F. Zehra Küçükbay, Andrea Ageli, Gianluca Bartolucci, Claudiu T. Supuran

**Affiliations:** a Department of Chemistry, Faculty of Arts and Sciences, İnönü University, Malatya, Turkey; b Department of Basic Pharmaceutical Sciences, Faculty of Pharmacy, İnönü University, Malatya, Turkey; c Dipartimento Neurofarba, Sezione Di Scienze Farmaceutiche E Nutraceutiche, Università degli Studi di Firenze, Florence, Italy

**Keywords:** Carbonic anhydrase, inhibitor, sulphonamide, amino acid, conjugate

## Abstract

A series of amino acid–sulphonamide conjugates was prepared through benzotriazole mediated coupling reactions and characterised by ^1^H-NMR, ^13^C-NMR, MS, and FTIR spectroscopic techniques as well as elemental analysis. The carbonic anhydrase (CA, EC 4.2.1.1) inhibitory activity of the new compounds was determined against four human (h) isoforms, hCA I, hCA II, hCA VA, and hCA XII. Most of the synthesised compounds showed effective *in vitro* CA inhibitory properties. The new amino acid–sulphonamide conjugates showed potent inhibitory activity against hCA II, some of them at subnanomolar levels, exhibiting more effective inhibitory activity compared to the standard drug acetazolamide. Some of these sulphonamides were also found to be effective inhibitors of hCA I, hCA VA, and hCA XII, with activity from the low to high nanomolar range.

## 1. Introduction

The environmentally friendly synthesis of biologically active molecules is one of the most important issues in medicinal chemistry^1^. Sulphonamides are one of the oldest antimicrobial compounds class, discovered in the 1930s^2^ and they are present in many effective drugs, due to their excellent chemical and biological properties^3^. Today, many sulphonamides are used for the treatment of urinary infections, burns, especially in combination with dihydrofolate reductase inhibitors such as trimethoprim^4^. On the other hand, with the discovery of strong carbonic anhydrase (CA, EC 4.2.1.1) inhibition properties of primary sulphonamides and the relationship with various pathologies, including cancer^5–7^, studies in this field have gained a different dimension. However, the widespread use of sulphonamide antibacterials also leads to the development of bacterial resistance. For this reason, the development of novel classes of antimicrobial compounds with less side effect and improved selectivity is urgently needed. Therefore, synthesis of new sulphonamide derivatives is an active research topic for organic and pharmaceutical chemists^8^^,^^9^. Both sulphonamides^10–12^ and amino acids^13–15^ have been reported to have various biological activities, but there are few reports of their successful combination in one molecule as a hybrid drug candidate^16–18^. Due to their antimicrobial and CA inhibitory properties, both primary and secondary sulphonamide derivatives are under investigation to determine compounds possessing the highest activity with possibly few side-effects^19^^,^^20^. For this reason, many studies on sulphonamide derivatives have been done in recent years^3^^,^^8^^,^^9^^,^^12^^,^^18^^,^^21^. Moreover, our group previously observed the CA inhibitory properties of some primary sulphonamide derivatives incorporating dipeptide and amino acid moieties, which inhibited some pharmacologically relevant CA isoforms at nanomolar levels^16–18^. Following our synthetic and CA inhibition screening work on dipeptide and *N*-protected amino acid–sulphonamide conjugates, we synthesised and explored the inhibitory activity of new N-protected amino acid–sulphonamide conjugates against hCA I, II, VA, and XII enzymes, in the search of more effective and isoform-selective CA inhibitors (CAIs).

## 2. Material and methods

### 2.1. Chemistry

The starting materials and reagents used in the reactions were supplied commercially by Aldrich, Acros, Merck, AFG Bioscience, Bachem, Alfa Aesar, or Fluorochem (all from Milan, Italy). Nuclear magnetic resonance (^1^H-NMR, ^13^C-NMR) spectra were recorded using a Bruker Advance III 400 or 300 MHz spectrometers in DMSO-d_6_. Chemical shifts are reported in parts per million (ppm) and the coupling constants (*J*) are expressed in Hertz (Hz). The assignment of exchangeable protons (OH and NH) was confirmed by the addition of D_2_O. Positive or negative-ion electrospray ionisation (ESI) mass spectra were recorded on a double-focusing Finnigan MAT 95 instrument with BE geometry. Analytical thin-layer chromatography (TLC) was carried out on Merck silica gel F-254 plates. All microwave-assisted reactions were carried out in a microwave oven system manufactured by Milestone (Milestone Start S Microwave Labstation for Synthesis). Infra-red spectra were recorded with ATR equipment in the range 4000–650 cm^−1^ on a Perkin Elmer Spectrum one FTIR spectrophotometer. Elemental analyses were performed with a LECO CHNS-932 elemental analyser. Melting points were recorded using an Electrothermal-9200 melting point apparatus and are uncorrected. All starting N-protected dipeptides were prepared according to literature procedures^17^^,^^18^^,^^22–24^. The compounds **5**, **10,** and **18** are commercially available in the Scifinder database, but since no information is available, information on syntheses and characterisations is also provided (Spectral data are presented in Supplementary Material).

### 2.2. General procedure for the synthesis of amino acid–sulphonamide conjugates, 1–12

N-protected aminoacylbenzotriazole (1.0 equiv.) and 4-(2-aminoethyl)benzenesulphonamide (1.0 equiv.) were subjected to microwave irradiation (100 W, 70 °C) in DCM (5 ml) for 30 min. After completion of the reaction, all volatiles were removed by rotae vaporation and the obtained crude product was crystallised from ethanol.

#### 2.2.1. Benzyl (R)-(4-methyl-1-oxo-1-((4-sulphamoylphenethyl)amino)pentan-2-yl)carbamate (1)

White solid (75%); mp 174–175 °C; ^1^H-NMR (300 MHz, DMSO-d_6_): δ 8.02 (t, 1H, N*H*CH_2_CH_2_, *J* = 4.5 Hz), 7.74 (d, 2H, Ar-*H*, *J* = 6.0 Hz), 7.40–7.30 (m, 10H, OCON*H*CH + Ar-*H* + N*H_2_*), 5.09–4.98 (m, 2H, C*H_2_*OCO), 4.01–3.93 (m, 1H, OCONHC*H*), 3.35–3.27 (m, 2H, NHC*H_2_*CH_2_), 2.79 (t, 2H, NHCH_2_C*H_2_*, *J* = 7.5 Hz), 1.56–1.24 (m, 3H, CHC*H_2_*C*H*(CH_3_)_2_), 0.84 (t, 6H, CH(C*H_3_*)*_2_*, *J* = 6.0 Hz).

^13^C NMR (75 MHz, DMSO-d_6_): δ 172.2 (*C*ONHCH_2_CH_2_), 155.9 (CH_2_O*C*O), 143.6, 142.0, 137.1, 129.1, 128.3, 127.7, 127.7, 125.6 (Ar-*C*), 65.3 (*C*H_2_OCO), 53.2 (OCONH*C*H), 40.9 (CH*C*H_2_CH(CH_3_)_2_), 34.7 (CONHCH_2_*C*H_2_), 24.2 (CHCH_2_*C*H(CH_3_)_2_), 22.9 and 21.5 (CHCH_2_CH(*C*H_3_)*_2_*). _ν_(C–O)carbamate: 1651 cm^−1^, _ν_(C–O)amide: 1671 cm^−1^, _ν_(N-H)amine: 3304, 3676 cm^−1^. Anal. calculated for C_22_H_29_N_3_O_5_S: C, 59.04; H, 6.53; N, 9.39; S, 7.16. Found: C, 58.73; H, 6.19; N, 9.34; S, 7.09. HRMS *m/z* for C_22_H_29_N_3_O_5_S [M + H]^+^ calcd. 448.2, found 448.3; [M + Na]^+^ calcd. 470.2, found 470.3; [M + HCOO] ^−^ calcd. 492.2 found 492.3.

#### 2.2.2. Benzyl (R)-(4-(methylthio)-1-oxo-1-((4-sulphamoylphenethyl)amino)butan-2-yl)carbamate (2)

White solid (88%); mp 173–174 °C; ^1^H-NMR (300 MHz, DMSO-d_6_): δ 8.04 (t, 1H, N*H*CH_2_CH_2_, *J* = 4.5 Hz), 7.75 (d, 2H, Ar-*H*, *J* = 9.0 Hz), 7.48 (d, 1H, OCON*H*CH, *J* = 9.0 Hz), 7.41–7.31 (m, 9H, Ar-*H* + N*H_2_*), 5.09–4.98 (m, 2H, C*H_2_*OCO), 4.07–4.00 (m, 1H, OCONHC*H*), 3.36–3.27 (m, 2H, NHC*H_2_*CH_2_), 2.79 (t, 2H, NHCH_2_C*H_2_*, *J* = 6.0 Hz), 2.45–2.34 (m, 2H, CHCH_2_C*H_2_*SCH_3_), 2.02 (s, 3H, CHCH_2_CH_2_SC*H_3_*), 1.86–1.70 (m, 2H, CHC*H_2_*CH_2_SCH_3_).

^13^C-NMR (100 MHz, DMSO-d_6_): δ 171.4 (*C*ONHCH_2_CH_2_), 156.0 (CH_2_O*C*O), 143.6, 142.0, 137.0, 129.1, 128.3, 127.8, 127.7, 125.6 (Ar-*C*), 65.4 (*C*H_2_OCO), 53.9 (OCONH*C*H), 34.7 (CONHCH_2_*C*H_2_), 31.5 (CHCH_2_*C*H_2_SCH_3_), 29.7 (CHCH_2_CH_2_S*C*H_3_), 14.5 (CH*C*H_2_CH_2_SCH_3_). _ν_(C–O)carbamate: 1648 cm^−1^, _ν_(C–O)amide: 1677 cm^−1^, _ν_(N-H)amine: 3312 cm^−1^. Anal. calculated for C_21_H_27_N_3_O_5_S_2_: C, 54.18; H, 5.85; N, 9.03; S, 13.77. Found: C, 53.44; H, 5.85; N, 9.22; S, 13.06. HRMS *m/z* for C_21_H_27_N_3_O_5_S_2_ [M + H]^+^ calcd. 466.2 found 466.3; [M + Na]^+^ calcd. 488.1, found 488.3; [M + HCOO]^−^ calcd. 510.1 found 510.2.

#### 2.2.3. Benzyl (R)-(3-methyl-1-oxo-1-((4-sulphamoylphenethyl)amino)butan-2-yl)carbamate (3)

White solid (95%); mp 185–186 °C; ^1^H-NMR (300 MHz, DMSO-d_6_): δ 8.05 (t, 1H, N*H*CH_2_CH_2_, *J* = 4.5 Hz), 7.74 (d, 2H, Ar-*H*, *J* = 9.0 Hz), 7.41–7.23 (m, 10H, Ar-*H* + OCON*H*CH + N*H_2_*), 5.09–4.99 (m, 2H, C*H_2_*OCO), 3.78 (t, 1H, OCONHC*H*, *J* = 7.5 Hz), 3.41–3.26 (m, 2H, NHC*H_2_*CH_2_), 2.79 (t, 2H, NHCH_2_C*H_2_*, *J* = 6.0 Hz), 1.94–1.83 (m, 1H, C*H*(CH_3_)_2_), 0.79 (d, 6H, CH(C*H_3_*)*_2_*, *J* = 6.0 Hz).

^13^C NMR (75 MHz, DMSO-d_6_): δ 171.1 (*C*ONHCH_2_CH_2_), 156.1 (CH_2_O*C*O), 143.6, 142.0, 137.1, 129.1, 128.3, 127.7, 127.6, 125.6 (Ar-*C*), 65.3 (*C*H_2_OCO), 60.3 (OCONH*C*H), 39.6 (CONH*C*H_2_CH_2_), 34.7 (CONHCH_2_*C*H_2_), 30.1 (*C*H(CH_3_)_2_), 19.8 and 18.1 (CH(*C*H_3_)*_2_*). _ν_(C–O)carbamate: 1643 cm^−1^, _ν_(C–O)amide: 1690 cm^−1^, _ν_(N-H)amine: 3307, 3676 cm^−1^. Anal. calculated for C_21_H_27_N_3_O_5_S: C, 58.18; H, 6.28; N, 9.69; S, 7.40. Found: C, 57.75; H, 5.88; N, 9.65; S, 7.09. HRMS *m/z* for C_21_H_27_N_3_O_5_S [M + H]^+^ calcd. 434.2, found 434.3; [M + Na]^+^ calcd. 456.2, found 456.3; [M + HCOO]^−^ calcd. 478.2, found 478.3.

#### 2.2.4. Benzyl (S)-(1-oxo-3-(phenylthio)-1-((4-sulphamoylphenethyl)amino)propan-2-yl)carbamate (4)

White solid (85%); mp 117–118 °C; ^1^H-NMR (300 MHz, DMSO-d_6_): δ 8.25 (t, 2H, N*H*CH_2_CH_2_, *J* = 6.0 Hz), 7.75 (d, 2H, Ar-*H*, *J* = 6.0 Hz), 7.67 (d, 1H, OCON*H*CH_,_ *J* = 9.0 Hz), 7.41–7.21 (m, 14H, Ar-*H* + N*H_2_*), 5.11–5.00 (m, 2H, C*H_2_*OCO), 4.18–4.11 (m, 1H, OCONHC*H*), 3.35–3.26 (s, 3H, C*H_2_*SPh + NHC*H_2_*CH_2_), 3.11–3.04 (m, 1H, C*H_2_*SPh), 2.79 (t, 2H, NHCH_2_C*H_2_*, *J* = 6.0 Hz).

^13^C NMR (75 MHz, DMSO-d_6_): δ 169.8 (*C*ONHCH_2_CH_2_), 155.9 (CH_2_O*C*O), 143.6, 142.0, 136.9, 135.7, 129.2, 129.1, 128.3, 128.3, 127.8, 127.7, 125.9, 125.6 (Ar-*C*), 65.6 (*C*H_2_OCO), 54.2 (OCONH*C*H), 40.5 (NH*C*H_2_CH_2_), 34.9 (*C*H_2_SPh), 34.6 (CONHCH_2_*C*H_2_). _ν_(C–O)carbamate: 1652 cm^−1^, _ν_(C–O)amide: 1712 cm^−1^, _ν_(N-H)amine: 3318, 3676 cm^−1^. Anal. calculated for C_25_H_27_N_3_O_5_S_2_: C, 58.46; H, 5.30; N, 8.18; S, 12.48. Found: C, 57.34; H, 5.13; N, 8.13; S, 11.87. HRMS *m/z* for C_25_H_27_N_3_O_5_S_2_ [M + H]^+^ calcd. 514.2, found 514.3; [M + Na]^+^ calcd. 536.2, found 456.3; [M + HCOO]^−^ calcd. 558.2, found 558.2.

#### 2.2.5. tert-Butyl (R)-(1-oxo-1-((4-sulphamoylphenethyl)amino)propan-2-yl)carbamate (5)

White solid (90%); mp 165–166 °C; ^1^H-NMR (300 MHz, DMSO-d_6_): δ 7.88 (t, 1H, N*H*CH_2_CH_2_, *J* = 4.5 Hz), 7.74 (d, 2H, Ar-*H*, *J* = 9.0 Hz), 7.39 (d, 2H, Ar-*H*, *J* = 9.0 Hz), 7.30 (s, 2H, N*H_2_*), 6.85 (d, 1H, OCON*H*CH_,_ *J* = 9.0 Hz), 3.95–3.85 (m, 1H, OCONHC*H*), 3.38–3.25 (m, 2H, NHC*H_2_*CH_2_), 2.78 (t, 2H, NHCH_2_C*H_2_*, *J* = 6.0 Hz), 1.38 (s, 9H, OC(C*H_3_*)*_3_*), 1.12 (d, 3H, CHC*H_3_*, *J* = 9.0 Hz).

^13^C NMR (75 MHz, DMSO-d_6_): δ 172.7 (*C*ONHCH_2_CH_2_), 155.0 ((CH_3_)_3_CO*C*O), 143.6, 142.0, 129.1, 125.6 (Ar-*C*), 78.0 ((CH_3_)_3_*C*OCO), 49.7 (OCONH*C*H), 34.7 (CONHCH_2_*C*H_2_), 28.2 (OC(*C*H_3_)*_3_*), 18.3 (CH*C*H_3_). _ν_(C–O)carbamate: 1664 cm^−1^, _ν_(C–O)amide: 1685 cm^−1^, _ν_(N-H)amine: 3373 cm^−1^. Anal. calculated for C_16_H_25_N_3_O_5_S: C, 51.74; H, 6.78; N, 11.31; S, 8.63. Found: C, 51.65; H, 6.67; N, 11.25; S, 8.26. HRMS *m/z* for C_16_H_25_N_3_O_5_S [M + Na]^+^ calcd. 394.2, found 394.2; [M + HCOO]^−^ calcd. 416.2, found 416.1; [M-H]^−^ calcd. 370.2, found 370.1.

#### 2.2.6. (9H-fluoren-9-yl)methyl (2-oxo-2-((4-sulphamoylphenethyl)amino)ethyl)carbamate (6)

White solid (91%); mp 134–135 °C; ^1^H-NMR (300 MHz, DMSO-d_6_): δ 7.97 (t, 1H, N*H*CH_2_CH_2_, *J* = 4.5 Hz), 7.90 (d, 2H, Ar-*H*, *J* = 9.0 Hz), 7.74 (t, 4H, Ar-H, *J* = 7.5 Hz), 7.54 (t, 1H, OCON*H*CH_2,_ *J* = 6.0 Hz), 7.45–7.32 (m, 8H, Ar-*H* + N*H_2_*), 4.32–4.24 (m, 3H, C*H*C*H_2_*OCO), 3.58 (d, 2H, OCONHC*H_2_*_,_ *J* = 6.0 Hz), 3.34–3.29 (m, 2H, NHC*H_2_*CH_2_), 2.80 (t, 2H, NHCH_2_C*H_2_*, *J* = 7.5 Hz).

^13^C NMR (75 MHz, DMSO-d_6_): δ 169.0 (*C*ONHCH_2_CH_2_), 156.5 (CH_2_O*C*O), 143.8, 143.6, 142.0, 140.7, 129.1, 127.6, 127.1, 125.7, 125.2, 120.1 (Ar-*C*), 65.7 (*C*H_2_OCO), 46.6 (*C*HCH_2_OCO), 43.4 (OCONH*C*H_2_), 34.8 (CONHCH_2_*C*H_2_). _ν_(C–O)carbamate: 1661 cm^−1^, _ν_(C–O)amide: 1700 cm^−1^, _ν_(N-H)amine: 3312 cm^−1^. Anal. calculated for C_25_H_25_N_3_O_5_S: C, 62.62; H, 5.25; N, 8.76; S, 6.69. Found: C, 61.66; H, 5.29; N, 8.75; S, 6.06. HRMS *m/z* for C_25_H_25_N_3_O_5_S [M + H]^+^ calcd. 480.2, found 480.3; [M + NH_4_]^+^ calcd. 497.2, found 497.3; [M + HCOO]^−^ calcd. 524,2 found 524.2.

#### 2.2.7. (9H-fluoren-9-yl)methyl (S)-(1-oxo-3-phenyl-1-((4-sulphamoylphenethyl)amino)propan-2-yl)carbamate (7)

White solid (93%); mp 174–175 °C; ^1^H-NMR (300 MHz, DMSO-d_6_): δ 8.15 (t, 1H, N*H*CH_2_CH_2_, *J* = 6.0 Hz), 7.89 (d, 2H, Ar-*H*, *J* = 9.0 Hz), 7.75 (t, 2H, Ar-*H*, *J* = 6.0 Hz), 7.68–7.63 (m, 3H, Ar-*H* + OCON*H*CH), 7.74–7.17 (m, 13H, Ar-*H* + N*H_2_*), 4.23–4.14 (m, 4H, C*H*C*H_2_*OCONHC*H*), 3.33–3.26 (m, 2H, NHC*H_2_*CH_2_), 2.96–2.90 (m, 1H, CHC*H_2_*Ph), 2.82–2.75 (m, 3H, CHC*H_2_*Ph + NHCH_2_C*H_2_*).

^13^C NMR (75 MHz, DMSO-d_6_): δ 171.4 (*C*ONHCH_2_CH_2_), 155.7 (CH_2_O*C*O), 143.8, 143.7, 143.6, 142.0, 140.6, 138.2, 129.9, 129.2, 128.0, 127.6, 127.0, 126.2, 125.6, 125.4, 125.3, 120.1 (Ar-*C*), 65.6 (*C*H_2_OCO), 56.2 (*C*HCH_2_Ph), 46.5 (*C*HCH_2_OCO), 37.6 (CH*C*H_2_Ph), 34.7 (CONHCH_2_*C*H_2_). _ν_(C–O)carbamate: 1645 cm^−1^, _ν_(C–O)amide: 1683 cm^−1^, _ν_(N-H)amine: 3318 cm^−1^. Anal. calculated for C_32_H_31_N_3_O_5_S: C, 67.47; H, 5.49; N, 7.38; S, 5.63. Found: C, 66.24; H, 5.74; N, 7.47; S, 5.15. HRMS *m/z* for C_32_H_31_N_3_O_5_S [M + H]^+^ calcd. 592.2, found 592.4; [M + Na]^+^ calcd. 570.2, found 570.4; [M + HCOO]^−^ calcd. 614.2, found 614.3.

#### 2.2.8. Benzyl (R)-(3-(1H-indol-3-yl)-1-oxo-1-((4-sulphamoylphenethyl)amino)propan-2-yl)carbamate (8)

White solid (88%); mp 194–195 °C; ^1^H-NMR (400 MHz, DMSO-d_6_): δ 10.82 (s, 1H, Indole-N*H*), 8.14 (t, 2H, N*H*CH_2_CH_2_, *J* = 4.0 Hz), 7.74 (d, 2H, Ar-*H*, *J* = 8.0 Hz), 7.63 (d, 1H, OCON*H*CH_,_ *J* = 8.0 Hz), 7.41–7.27 (m, 11H, Ar-*H*), 7.15–6.97 (m, 3H, Ar-*H* + N*H_2_*), 4.97 (s, 2H, C*H_2_*OCO), 4.26–4.20 (m, 1H, OCONHC*H*), 3.37–3.24 (m, 2H, NHC*H_2_*CH_2_), 3.07–3.03 (m, 1H, (3-Indolyl)C*H_2_*CH), 2.93–2.87 (m, 1H, (3-Indolyl)C*H_2_*CH), 2.73 (t, 2H, NHCH_2_C*H_2_*, *J* = 6.0 Hz).

^13^C NMR (100 MHz, DMSO-d_6_): δ 172.3 (*C*ONHCH_2_CH_2_), 156.3 (CH_2_O*C*O), 144.2, 142.5, 137.5, 136.5, 129.6, 128.8, 128.2, 128.0, 127.7, 126.1, 124.3, 121.3, 119.0, 118.7, 111.8, 110.7 (Ar-*C*), 65.7 (*C*H_2_OCO), 56.1 (OCONH*C*H), 35.2 (CONHCH_2_*C*H_2_), 28.4 ((3-Indolyl)*C*H_2_CH). _ν_(C–O)carbamate: 1639 cm^−1^, _ν_(C–O)amide: 1687 cm^−1^, _ν_(N-H)amine: 3286, 3430, 3681 cm^−1^. Anal. calculated for C_27_H_28_N_4_O_5_S: C, 62.29; H, 5.42; N, 10.76; S, 6.16. Found: C, 62.92; H, 5.41; N, 10.84; S, 5.58. HRMS *m/z* for C_27_H_28_N_4_O_5_S [M + H]^+^ calcd. 521.2, found 521.3; [M-H]^−^ calcd. 519.2, found 519.2.

#### 2.2.9. tert-Butyl (S)-(4-(methylthio)-1-oxo-1-((4-sulphamoylphenethyl)amino)butan-2-yl)carbamate (9)

White solid (89%); mp 172–173 °C; ^1^H-NMR (400 MHz, DMSO-d_6_): δ 7.94 (t, 1H, N*H*CH_2_CH_2_, *J* = 4.0 Hz), 7.74 (d, 2H, Ar-*H*, *J* = 8.0 Hz), 7.39 (d, 2H, Ar-*H*, *J* = 8.0 Hz), 7.30 (s, 2H, N*H_2_*), 6.93 (d, 1H, OCON*H*CH_,_ *J* = 8.0 Hz), 3.98–3.93 (m, 1H, OCONHC*H*), 3.34–3.26 (m, 2H, NHC*H_2_*CH_2_), 2.79 (t, 2H, NHCH_2_C*H_2_*, *J* = 6.0 Hz), 2.46–2.34 (m, 2H, CHCH_2_C*H_2_*SCH_3_), 2.02 (s, 3H, CHCH_2_CH_2_SC*H_3_*), 1.85–1.67 (m, 2H, CHC*H_2_*CH_2_SCH_3_), 1.39 (s, 9H, OC(C*H_3_*)*_3_*).

^13^C NMR (100 MHz, DMSO-d_6_): δ 172.2 (*C*ONHCH_2_CH_2_), 155.8 ((CH_3_)_3_CO*C*O), 144.2, 142.5, 129.62, 126.1 (Ar-*C*), 78.6 ((CH_3_)_3_*C*OCO), 54.0 (OCONH*C*H), 40.3 (CONH*C*H_2_CH_2_), 35.2 (CONHCH_2_*C*H_2_), 32.2 (CHCH_2_*C*H_2_SCH_3_), 30.2 (CHCH_2_CH_2_S*C*H_3_), 28.7 (OC(*C*H_3_)*_3_*), 15.1 (CH*C*H_2_CH_2_SCH_3_). _ν_(C–O)carbamate: 1651 cm^−1^, _ν_(C–O)amide: 1680 cm^−1^, _ν_(N-H)amine: 3250, 3317, 3337 cm^−1^. Anal. calculated for C_18_H_29_N_3_O_5_S_2_: C, 50.10; H, 6.77; N, 9.74; S, 14.86. Found: C, 49.25; H, 7.04; N, 9.89; S, 15.29. HRMS *m/z* for C_18_H_29_N_3_O_5_S [M + Na]^+^ calcd. 454.1, found 454.3; [M + HCOO]^−^ calcd. 476.2, found 476.2.

#### 2.2.10. tert-Butyl (R)-(3-methyl-1-oxo-1-((4-sulphamoylphenethyl)amino)butan-2-yl)carbamate (10)

White solid (90%); mp 160–161 °C; ^1^H-NMR (400 MHz, DMSO-d_6_): δ 8.02 (t, 1H, N*H*CH_2_CH_2_, *J* = 6.0 Hz), 7.79 (d, 2H, Ar-*H*, *J* = 12.0 Hz), 7.39 (d, 2H, Ar-*H*, *J* = 8.0 Hz), 7.35 (s, 2H, N*H_2_*), 6.66 (d, 1H, OCON*H*CH_,_ *J* = 12.0 Hz), 3.76 (t, 1H, OCONHC*H, J* = 8.0 Hz), 3.41–3.30 (m, 2H, NHC*H_2_*CH_2_), 2.84 (t, 2H, NHCH_2_C*H_2_*, *J* = 8.0 Hz), 1.94–1.86 (m, 1H, C*H*(CH_3_)_2_), 1.45 (s, 9H, OC(C*H_3_*)*_3_*), 0.83 (d, 6H, CH(C*H_3_*)*_2_*, *J* = 4.0 Hz).

^13^C NMR (100 MHz, DMSO-d_6_): δ 171.8 (*C*ONHCH_2_CH_2_), 155.9 ((CH_3_)_3_CO*C*O), 144.1, 142.5, 129.6, 126.1 (Ar-*C*), 78.4 ((CH_3_)_3_*C*OCO), 60.3 (OCONH*C*H), 40.1 (CONH*C*H_2_CH_2_), 35.2 (CONHCH_2_*C*H_2_), 30.8 (*C*H(CH_3_)_2_), 28.7 (OC(*C*H_3_)*_3_*), 19.7 and 18.6 (CH(*C*H_3_)*_2_*). _ν_(C–O)carbamate: 1650 cm^−1^, _ν_(C–O)amide: 1679 cm^−1^, _ν_(N-H)amine: 3340, 3681 cm^−1^. Anal. calculated for C_18_H_29_N_3_O_5_S: C, 54.12; H, 7.32; N, 10.52; S, 8.02. Found: C, 53.80; H, 7.48; N, 10.92; S, 6.81. HRMS *m/z* for C_18_H_29_N_3_O_5_S [M + Na]^+^ calcd. 422.2, found 422.3; [M + HCOO]^−^ calcd. 444.2, found 444.2.

#### 2.2.11. tert-Butyl ((2R,3R)-3-methyl-1-oxo-1-((4-sulphamoylphenethyl)amino)pentan-2-yl)carbamate (11)

White solid (91%); mp 191–192 °C; ^1^H-NMR (400 MHz, DMSO-d_6_): δ 7.98 (t, 1H, N*H*CH_2_CH_2_, *J* = 4.0 Hz), 7.74 (d, 2H, Ar-*H*, *J* = 8.0 Hz), 7.40 (d, 2H, Ar-*H*, *J* = 8.0 Hz), 7.28 (s, 2H, N*H_2_*), 6.63 (d, 1H, OCON*H*CH_,_ *J* = 8.0 Hz), 3.75 (t, 1H, OCONHC*H, J* = 8.0 Hz), 3.39–3.26 (m, 2H, NHC*H_2_*CH_2_), 2.79 (t, 2H, NHCH_2_C*H_2_*, *J* = 6.0 Hz), 1.63–1.61 (m, 1H, CHC*H*(CH_3_)CH_2_CH_3_), 1.39 (s, 10H, OC(C*H_3_*)*_3_* + CHCH(CH_3_)C*H_2_*CH_3_), 1.10–1.00 (m, 1H, CHCH(CH_3_)C*H_2_*CH_3_), 0.79 (t, 3H, CHCH(CH_3_)CH_2_C*H_3_*, *J* = 8.0 Hz), 0.74 (d, 3H, CHCH(C*H_3_*)CH_2_CH_3_, *J* = 8.0 Hz).

^13^C NMR (100 MHz, DMSO-d_6_): δ 171.9 (*C*ONHCH_2_CH_2_), 155.8 ((CH_3_)_3_CO*C*O), 144.1, 142.6, 129.6, 126.1 (Ar-*C*), 78.4 ((CH_3_)_3_*C*OCO), 59.3 (OCONH*C*H), 40.1 (CONH*C*H_2_CH_2_), 36.9 (CHC*H*(CH_3_)CH_2_CH_3_), 35.2 (CONHCH_2_*C*H_2_), 28.7 (OC(C*H_3_*)*_3_*), 24.8 (CHCH(CH_3_)*C*H_2_CH_3_), 15.8 (CHCH(CH_3_)CH_2_*C*H_3_), 11.4 (CHCH(C*H_3_*)CH_2_CH_3_). _ν_(C–O)carbamate: 1651 cm^−1^, _ν_(C–O)amide: 1679 cm^−1^, _ν_(N-H)amine: 3286, 3312, 3340 cm^−1^. Anal. calculated for C_19_H_31_N_3_O_5_S: C, 55.19; H, 7.56; N, 10.16; S, 7.75. Found: C, 55.13; H, 7.33; N, 10.27; S, 7.01. HRMS *m/z* for C_19_H_31_N_3_O_5_S [M + Na]^+^ calcd. 436.2, found 436.3; [M-H]^−^ calcd. 412.2, found 412.2; [M + HCOO]^−^calcd. 458.2, found 458.2.

#### 2.2.12. Benzyl (R)-(1,4-dioxo-1,4-bis((4-sulphamoylphenethyl)amino)butan-2-yl)carbamate (12)

White solid (73%); mp 249–250 °C; ^1^H-NMR (400 MHz, DMSO-d_6_): δ 8.02 (t, 1H, N*H*CH_2_CH_2_, *J* = 6.0 Hz), 7.96 (t, 1H, N*H*CH_2_CH_2_, *J* = 6.0 Hz), 7.75 (d, 4H, Ar-*H*, *J* = 8.0 Hz), 7.40–7.32 (m, 14H, OCON*H*CH + Ar-*H* + N*H_2_*), 5.09–5.00 (m, 2H, C*H_2_*OCO), 4.35–4.30 (m, 1H, OCONHC*H*), 3.29 (bs, 4H, NHC*H_2_*CH_2_ + NHC*H_2_*CH_2_), 2.80–2.74 (m, 4H, NHCH_2_C*H_2_* + NHCH_2_C*H_2_*), 2.49–2.33 (m, 2H, CHC*H_2_*CO).

^13^C NMR (100 MHz, DMSO-d_6_): δ 171.6 (*C*ONHCH_2_CH_2_), 169.8 (*C*ONHCH_2_CH_2_), 156.2 (CH_2_O*C*O), 144.2, 142.5, 137.4, 129.7, 129.6, 128.8, 128.3, 128.2, 126.2, 126.1 (Ar-*C*), 66.0 (*C*H_2_OCO), 52.3 (OCONH*C*H), 40.5 (CONH*C*H_2_CH_2_), 40.4 (CONH*C*H_2_CH_2_), 38.1 (CH*C*H_2_CO), 35.3 (NHCH_2_*C*H_2_), 35.2 (NHCH_2_*C*H_2_). _ν_(C–O)carbamate: 1634 cm^−1^, _ν_(C–O)amide: 1684 cm^−1^, _ν_(N-H)amine: 3311 cm^−1^. Anal. calculated for C_28_H_33_N_5_O_8_S_2_: C, 53.24; H, 5.27; N, 11.09; S, 10.15. Found: C, 52.99; H, 5.46; N, 10.73; S, 9.91. HRMS *m/z* for C_28_H_33_N_5_O_8_S_2_ [M + H]^+^ calcd. 632.2, found 632.4; [M-H]^−^ calcd. 630.2, found 630.3.

### 2.3. General procedure for the synthesis of amino acid–sulphonamide conjugates, 13–24

N-protected aminoacylbenzotriazole (1.0 equiv.), (4-sulphamoylphenyl)methanaminium chloride (1.0 equiv.), and Et_3_N (2.5 equiv.) were subjected to microwave irradiation (100 W, 70 °C) in DCM (5 ml) for 30 min. After completion of the reaction (monitoring TLC plate), all volatiles were removed by rotavapour and the obtained crude product was crystallised from ethanol.

#### 2.3.1. Benzyl (R)-(4-methyl-1-oxo-1-((4-sulphamoylbenzyl)amino)pentan-2-yl)carbamate (13)

White solid (74%); mp 173–174 °C; ^1^H-NMR (300 MHz, DMSO-d_6_): δ 8.60 (t, 1H, CON*H*CH_2_, *J* = 6.0 Hz), 7.77 (d, 2H, Ar-*H*, *J* = 9.0 Hz), 7.52 (d, 1H, OCON*H*CH, *J* = 9.0 Hz), 7.43–7.31 (m, 9H, Ar-*H* + N*H_2_*), 5.05 (d, 2H, C*H_2_*OCO), 4.34 (d, 2H, CONHC*H_2_*, *J* = 3.0 Hz), 4.11–4.03 (m, 1H, OCONHC*H*), 1.67–1.39 (m, 3H, CHC*H_2_*C*H*(CH_3_)_2_), 0.90–0.85 (m, 6H, CH(C*H_3_*)*_2_*).

^13^C NMR (75 MHz, DMSO-d_6_): δ 172.6 (*C*ONHCH_2_), 156.0 (CH_2_O*C*O), 143.6, 142.5, 137.0, 128.3, 127.8, 127.7, 127.3, 125.6 (Ar-*C*), 65.4 (*C*H_2_OCO), 53.2 (OCONH*C*H), 41.7 (CONH*C*H_2_), 40.6 (CH*C*H_2_CH(CH_3_)_2_), 24.2 (CHCH_2_*C*H(CH_3_)_2_), 22.9 and 21.4 (CHCH_2_CH(*C*H_3_)*_2_*). _ν_(C–O)carbamate: 1659 cm^−1^, _ν_(C–O)amide: 1683 cm^−1^, _ν_(N-H)amine: 3304 cm^−1^. Anal. calculated for C_21_H_27_N_3_O_5_S: C, 58.18; H, 6.28; N, 9.69; S, 7.40. Found: C, 57.56; H, 5.88; N, 9.64; S, 7.15. HRMS *m/z* for C_21_H_27_N_3_O_5_S [M + H]^+^ calcd. 434.2, found 434.3*;* [M + Na]^+^ calcd. 466.2, found 466.3; [M + HCOO]^−^ calcd. 478.2, found 478.2.

#### 2.3.2. Benzyl (R)-(4-(methylthio)-1-oxo-1-((4-sulphamoylbenzyl)amino)butan-2-yl)carbamate (14)

White solid (80%); mp 167–168 °C; ^1^H-NMR (300 MHz, DMSO-d_6_): δ 8.59 (t, 1H, CON*H*CH_2_, *J* = 6.0 Hz), 7.78 (d, 2H, Ar-*H*, *J* = 9.0 Hz), 7.58 (d, 1H, OCON*H*CH, *J* = 6.0 Hz), 7.44–7.33 (m, 9H, Ar-*H* + N*H_2_*), 5.10–5.01 (m, 2H, C*H_2_*OCO), 4.35 (d, 2H, CONHC*H_2_*, *J* = 6.0 Hz), 4.18–4.11 (m, 1H, OCONHC*H*), 2.51–2.40 (m, 2H, CHCH_2_C*H_2_*SCH_3_), 2.03 (s, 3H, CHCH_2_CH_2_SC*H_3_*), 1.94–1.80 (m, 2H, CHC*H_2_*CH_2_SCH_3_).

^13^C-NMR (75 MHz, DMSO-d_6_): δ 171.7 (*C*ONHCH_2_), 156.1 (CH_2_O*C*O), 143.6, 142.5, 136.9, 128.3, 127.8, 127.7, 127.3, 125.6 (Ar-*C*), 65.5 (*C*H_2_OCO), 54.0 (OCONH*C*H), 41.7 (CONH*C*H_2_), 31.3 (CHCH_2_*C*H_2_SCH_3_), 29.7 (CHCH_2_CH_2_S*C*H_3_), 14.6 (CH*C*H_2_CH_2_SCH_3_). _ν_(C–O)carbamate: 1646 cm^−1^, _ν_(C–O)amide: 1692 cm^−1^, _ν_(N-H)amine: 3331 cm^−1^, _ν_(N-H)amine: 3331 cm^−1^. Anal. calculated for C_20_H_25_N_3_O_5_S_2_: C, 53.20; H, 5.58; N, 9.31; S, 14.20. Found: C, 53.15; H, 5.39; N, 9.22; S, 13.30. HRMS *m/z* for C_20_H_25_N_3_O_5_S_2_ [M + H]^+^ calcd. 452.1, found 452.2; [M + HCOO]^−^ calcd. 496.2, found 496.2.

#### 2.3.3. Benzyl (R)-(3-methyl-1-oxo-1-((4-sulphamoylbenzyl)amino)butan-2-yl)carbamate (15)

White solid (89%); mp 141–142 °C; ^1^H-NMR (300 MHz, DMSO-d_6_): δ 8.58 (t, 1H, CON*H*CH_2_, *J* = 4.5 Hz), 7.77 (d, 2H, Ar-*H*, *J* = 9.0 Hz), 7.45–7.33 (m, 10H, Ar-*H* + OCON*H*CH + N*H_2_*), 5.06 (s, 2H, C*H_2_*OCO), 4.36 (d, 2H, CONHC*H_2_*, *J* = 3.0 Hz), 3.87 (t, 1H, OCONHC*H*, *J* = 6.0 Hz), 2.05–1.94 (m, 1H, C*H*(CH_3_)_2_), 0.86 (d, 6H, CH(C*H_3_*)*_2_*, *J* = 6.0 Hz).

^13^C NMR (75 MHz, DMSO-d_6_): δ 171.4 (*C*ONHCH_2_), 156.2 (CH_2_O*C*O), 143.5, 142.6, 137.0, 128.3, 127.8, 127.7, 127.5, 125.6 (Ar-*C*), 65.4 (*C*H_2_OCO), 60.5 (OCONH*C*H), 41.7 (CONH*C*H_2_), 30.0 (*C*H(CH_3_)_2_), 19.2 and 18.2 (CH(*C*H_3_)*_2_*). _ν_(C–O)carbamate: 1649 cm^−1^, _ν_(C–O)amide: 1688 cm^−1^, _ν_(N-H)amine: 3287 cm^−1^. Anal. calculated for C_20_H_25_N_3_O_5_S: C, 57.26; H, 6.01; N, 10.02; S, 7.64. Found: C, 57.16; H, 6.22; N, 9.72; S, 7.11. HRMS *m/z* for C_20_H_25_N_3_O_5_S [M + H]^+^ calcd. 420.2, found 420.2; [M + Na]^+^ calcd. 442.1, found 442.3; [M + HCOO]^−^calcd. 464.2, found 464.2.

#### 2.3.4. Benzyl (S)-(1-oxo-3-(phenylthio)-1-((4-sulphamoylbenzyl)amino)propan-2-yl)carbamate (16)

White solid (72%); mp 196–197 °C; ^1^H-NMR (300 MHz, DMSO-d_6_): δ 8.76 (t, 2H, CON*H*CH_2_, *J* = 6.0 Hz), 7.76 (d, 2H, Ar-*H*, *J* = 9.0 Hz), 7.44–7.19 (m, 15H, Ar-*H* + OCON*H*CH + N*H_2_*), 5.11–4.99 (m, 2H, C*H_2_*OCO), 4.35 (d, 2H, CONHC*H_2_*, *J* = 3.0 Hz), 4.26–4.19 (m, 1H, OCONHC*H*), 3.42–3.35 (m, 1H, C*H_2_*SPh), 3.17–3.10 (m, 1H, C*H_2_*SPh).

^13^C NMR (75 MHz, DMSO-d_6_): δ 170.1 (*C*ONHCH_2_), 156.0 (CH_2_O*C*O), 143.3, 142.5, 136.84, 135.6, 129.1, 128.4, 128.3, 127.8, 127.7, 127.3, 126.0, 125.6 (Ar-*C*), 65.6 (*C*H_2_OCO), 54.3 (OCONH*C*H), 41.9 (CONH*C*H_2_), 34.8 (*C*H_2_SPh). _ν_(C–O)carbamate: 1648 cm^−1^, _ν_(C–O)amide:1673, 1689 cm^−1^, _ν_(N-H)amine: 3308, 3676 cm^−1^. Anal. calculated for C_24_H_25_N_3_O_5_S_2_: C, 57.70; H, 5.04; N, 8.41; S, 12.83. Found: C, 56.95; H, 4.94; N, 8.51; S, 11.79. HRMS *m/z* for C_24_H_25_N_3_O_5_S_2_ [M + H]^+^ calcd. 500.1, found 500.3; [M + HCOO]^−^ calcd. 544.1, found 544.2.

#### 2.3.5. tert-Butyl (2-oxo-2-((4-sulphamoylbenzyl)amino)ethyl)carbamate (17)

White solid (70%); mp 172–173 °C; ^1^H-NMR (300 MHz, DMSO-d_6_): δ 8.41 (t, 1H, CON*H*CH_2_, *J* = 6.0 Hz), 7.76 (d, 2H, Ar-*H*, *J* = 9.0 Hz), 7.43 (d, 2H, Ar-*H*, *J* = 9.0 Hz), 7.31 (s, 2H, N*H_2_*), 7.05 (d, 1H, OCON*H*CH_2_, *J* = 6.0 Hz), 4.35 (d, 2H, CONHCH_2_, *J* = 6.0 Hz), 3.58 (d, 2H, OCONHC*H_2_ J* = 6.0 Hz), 1.40 (s, 9H, OC(C*H_3_*)*_3_*).

^13^C NMR (75 MHz, DMSO-d_6_): δ 169.6 (*C*ONHCH_2_), 155.8 ((CH_3_)_3_CO*C*O), 143.6, 142.5, 127.4, 125.5 (Ar-*C*), 78.1 ((CH_3_)_3_*C*OCO), 43.4 (OCONH*C*H_2_), 41.6 (CONHCH_2_), 28.2 OC(*C*H_3_)*_3_*). _ν_(C–O)carbamate: 1644 cm^−1^, _ν_(C–O)amide: 1680 cm^−1^, _ν_(N-H)amine: 3326 cm^−1^. Anal. calculated for C_14_H_21_N_3_O_5_S: C, 48.97; H, 6.16; N, 12.24; S, 9.34. Found: C, 48.62; H, 6.01; N, 12.11; S, 9.25. HRMS *m/z* for C_14_H_21_N_3_O_5_S [M + Na]^+^ calcd. 366.1, found 366.1; [M-H]^−^ calcd. 342.1, found 342.1; [M + HCOO]^−^ calcd. 388.1, found 388.1.

#### 2.3.6. tert-Butyl (R)-(1-oxo-1-((4-sulphamoylbenzyl)amino)propan-2-yl)carbamate (18)

White solid (85%); mp 197–198 °C; ^1^H-NMR (300 MHz, DMSO-d_6_): δ 8.42 (t, 1H, CON*H*CH_2_, *J* = 6.0 Hz), 7.75 (d, 2H, Ar-*H*, *J* = 9.0 Hz), 7.42 (d, 2H, Ar-*H*, *J* = 9.0 Hz), 7.31 (s, 2H, N*H_2_*), 7.02 (d, 1H, OCON*H*CH, *J* = 9.0 Hz), 4.34 (d, 2H, CONHC*H_2_ J* = 6.0 Hz), 4.03–3.93 (m, 1H, OCONHC*H*), 1.40 (s, 9H, OC(C*H_3_*)*_3_*), 1.21 (d, 3H, CHC*H_3_*, *J* = 9.0 Hz).

^13^C NMR (75 MHz, DMSO-d_6_): δ 173.0 (*C*ONHCH_2_), 155.2 ((CH_3_)_3_CO*C*O), 143.7, 142.5, 127.1, 125.5 (Ar-*C*), 78.0 ((CH_3_)_3_*C*OCO), 49.9 (OCONH*C*H), 41.6 (CONH*C*H_2_), 28.2 (OC(*C*H_3_)*_3_*), 17.9 (CH*C*H_3_). _ν_(C–O)carbamate: 1650 cm^−1^, _ν_(C–O)amide: 1682 cm^−1^, _ν_(N-H)amine: 3328, 3676 cm^−1^. Anal. calculated for C_15_H_23_N_3_O_5_S: C, 50.41; H, 6.49; N, 11.76; S, 8.97. Found: C, 49.97; H, 6.73; N, 11.66; S, 8.88. HRMS *m/z* for C_15_H_23_N_3_O_5_S [M + Na]^+^ calcd. 380.1, found 380.2; [M-H]^−^ calcd. 356.1, found 356.1; [M + HCOO]^−^ calcd. 402.1, found 402.1.

#### 2.3.7. tert-Butyl (R)-(1-oxo-3-phenyl-1-((4-sulphamoylbenzyl)amino)propan-2-yl)carbamate (19)

White solid (95%); mp 198–199 °C; ^1^H-NMR (300 MHz, DMSO-d_6_): δ 8.55 (t, 1H, CON*H*CH_2_, *J* = 6.0 Hz), 7.72 (d, 2H, Ar-*H*, *J* = 9.0 Hz), 7.34–7.20 (m, 9H, Ar-*H* + N*H_2_*), 7.07 (d, 1H, OCON*H*CH_,_ *J* = 9.0 Hz), 4.33 (d, 2H, CONHC*H_2_*, *J* = 6.0 Hz), 4.24–4.16 (m, 1H, OCONHCH), 3.00–2.93 (m, 1H, CHC*H_2_*Ph), 2.83–2.75 (m, 1H, CHC*H_2_*Ph), 1.33 (s, 9H, OC(C*H_3_*)*_3_*).

^13^C NMR (75 MHz, DMSO-d_6_): δ 171.8 (*C*ONHCH_2_), 155.3 ((CH_3_)_3_CO*C*O), 143.3, 142.7, 138.1, 129.2, 128.0, 127.2, 126.9, 125.5 (Ar-*C*), 78.0 ((CH_3_)_3_*C*OCO), 56.0 (*C*HCH_2_Ph), 41.6 (CONHCH_2_), 37.3 (CH*C*H_2_Ph), 28.1 (OC(*C*H_3_)*_3_*). _ν_(C–O)carbamate: 1660 cm^−1^, _ν_(C–O)amide: 1677 cm^−1^, _ν_(N-H)amine: 3329, 3677 cm^−1^. Anal. calculated for C_21_H_27_N_3_O_5_S: C, 58.18; H, 6.28; N, 9.69; S, 7.40. Found: C, 57.87; H, 6.02; N, 9.60; S, 7.15. HRMS *m/z* for C_21_H_27_N_3_O_5_S [M + Na]^+^ calcd. 456.2, found 456.2; [M + HCOO]^−^ calcd. 478.2, found 478.2.

#### 2.3.8. (9H-fluoren-9-yl)methyl (S)-(1-oxo-3-phenyl-1-((4-sulphamoylbenzyl)amino)propan-2-yl)carbamate (20)

White solid (88%); mp 221–222 °C; ^1^H-NMR (300 MHz, DMSO-d_6_): δ 8.64 (t, 1H, CON*H*CH_2_, *J* = 6.0 Hz), 7.89 (d, 2H, Ar-*H*, *J* = 6.0 Hz), 7.74 (d, 2H, Ar-*H*, *J* = 9.0 Hz), 7.66 (t, 2H, Ar-*H*, *J* = 6.0 Hz), 7.44–7.21 (m, 14H, OCON*H*CH + Ar-*H* + N*H_2_*), 4.37–4.14 (m, 6H, C*H*C*H_2_*OCONHC*H*CONHC*H_2_*), 3.06–2.99 (m, 1H, CHC*H_2_*Ph), 2.89–2.81 (m, 1H, CHC*H_2_*Ph). ^13^C NMR (75 MHz, DMSO-d_6_): δ 171.5 (*C*ONHCH_2_), 155.8 (CH_2_O*C*O), 143.7, 143.3, 142.5, 140.6, 138.0, 129.2, 128.1, 127.6, 127.3, 127.0, 126.3, 125.6, 125.3, 120.1 (Ar-*C*), 65.6 (*C*H_2_OCO), 56.3 (*C*HCH_2_Ph), 46.5 (*C*HCH_2_OCO), 41.9 (CONH*C*H_2_), 37.5 (CH*C*H_2_Ph). _ν_(C–O)carbamate: 1641 cm^−1^, _ν_(C–O)amide: 1686 cm^−1^, _ν_(N-H)amine: 3263 cm^−1^. Anal. calculated for C_31_H_29_N_3_O_5_S: C, 67.01; H, 5.26; N, 7.56; S, 5.77. Found: C, 51.40; C, 67.78; H, 5.41; N, 7.71; S, 5.06. HRMS *m/z* for C_31_H_29_N_3_O_5_S [M + Na]^+^ calcd. 556.2, found 556.3; [M + HCOO]^−^calcd. 600.2, found 600.2.

#### 2.3.9. tert-Butyl (R)-(4-(methylthio)-1-oxo-1-((4-sulphamoylbenzyl)amino)butan-2-yl)carbamate (21)

White solid (85%); mp 142–143 °C; ^1^H-NMR (400 MHz, DMSO-d_6_): δ 8.48 (t, 1H, CON*H*CH_2_, *J* = 6.0 Hz), 7.75 (d, 2H, Ar-*H*, *J* = 8.0 Hz), 7.42 (d, 2H, Ar-*H*, *J* = 8.0 Hz), 7.32 (s, 2H, N*H_2_*), 7.09 (d, 1H, OCON*H*CH_,_ *J* = 8.0 Hz), 4.35 (d, 2H, CONHC*H_2_*, *J* = 8.0 Hz), 4.06–4.01 (m, 1H, OCONHC*H*), 2.49–2.40 (m, 2H, CHCH_2_C*H_2_*SCH_3_), 2.04 (s, 3H, CHCH_2_CH_2_SC*H_3_*), 1.89–1.80 (m, 2H, CHC*H_2_*CH_2_SCH_3_), 1.41 (s, 9H, OC(C*H_3_*)*_3_*).

^13^C NMR (100 MHz, DMSO-d_6_): δ 172.6 (*C*ONHCH_2_), 156.0 ((CH_3_)_3_CO*C*O), 144.2, 143.0, 127.7, 126.0 (Ar-*C*), 78.7 ((CH_3_)_3_*C*OCO), 54.2 (OCONH*C*H), 42.2 (CONH*C*H_2_), 31.8 (CHCH_2_*C*H_2_SCH_3_), 30.3 (CHCH_2_CH_2_S*C*H_3_), 28.7 (OC(*C*H_3_)*_3_*), 15.1 (CH*C*H_2_CH_2_SCH_3_). _ν_(C–O)carbamate: 1656 cm^−1^, _ν_(C–O)amide: 1695 cm^−1^, _ν_(N-H)amine: 3311, 3348 cm^−1^. Anal. calculated for C_17_H_27_N_3_O_5_S_2_: C, 48.90; H, 6.52; N, 10.06; S, 15.36. Found: C, C, 47.89; H, 6.78; N, 10.20; S, 14.53. HRMS *m/z* for C_17_H_27_N_3_O_5_S_2_ [M + Na]^+^ calcd. 440.1, found 440.2; [M-H]^−^ calcd. 416.1, found 416.2; [M + HCOO]^−^ calcd. 462.1, found 462.2.

#### 2.3.10. tert-Butyl (R)-(3-methyl-1-oxo-1-((4-sulphamoylbenzyl)amino)butan-2-yl)carbamate (22)

White solid (87%); mp 163–164 °C; ^1^H-NMR (400 MHz, DMSO-d_6_): δ 8.49 (t, 1H, CON*H*CH_2_, *J* = 6.0 Hz), 7.75 (d, 2H, Ar-*H*, *J* = 8.0 Hz), 7.43 (d, 2H, Ar-*H*, *J* = 8.0 Hz), 7.32 (s, 2H, N*H_2_*), 6.80 (d, 1H, OCON*H*CH_,_ *J* = 8.0 Hz), 4.35 (t, 2H, CONHC*H_2_*, *J* = 6.0 Hz), 3.77 (t, 1H, OCONHC*H, J* = 8.0 Hz), 1.99–1.91 (m, 1H, C*H*(CH_3_)_2_), 1.41 (s, 9H, OC(C*H_3_*)*_3_*), 0.85 (d, 6H, CH(C*H_3_*)*_2_*, *J* = 4.0 Hz).

^13^C NMR (100 MHz, DMSO-d_6_): δ 172.2 (*C*ONHCH_2_), 156.1 ((CH_3_)_3_CO*C*O), 144.1, 143.0, 127.9, 126.0 (Ar-*C*), 78.5 ((CH_3_)_3_*C*OCO), 60.7 (OCONH*C*H), 42.1 (CONH*C*H_2_), 30.4 (*C*H(CH_3_)_2_), 28.7 (OC(*C*H_3_)*_3_*), 19.8 and 18.9 (CH(*C*H_3_)*_2_*). _ν_(C–O)carbamate: 1654 cm^−1^, _ν_(C–O)amide: 1684 cm^−1^, _ν_(N-H)amine: 3333 cm^−1^. Anal. calculated for C_17_H_27_N_3_O_5_S: C, 52.97; H, 7.06; N, 10.90; S, 8.32. Found: C, 52.58; H, 7.20; N, 10.91; S, 7.89. HRMS *m/z* for C_17_H_27_N_3_O_5_S [M + Na]^+^ calcd. 408.2, found 408.2; [M-H]^−^ calcd. 384.2, found 384.2; [M + HCOO]^−^ calcd. 430.2, found 430.2.

#### 2.3.11. Benzyl (R)-(3-(1H-indol-3-yl)-1-oxo-1-((4-sulphamoylbenzyl)amino)propan-2-yl)carbamate (23)

White solid (91%); mp 184–185 °C; ^1^H-NMR (400 MHz, DMSO-d_6_): δ 10.86 (s, 1H, Indole-N*H*), 8.66 (t, 2H, CON*H*CH_2_, *J* = 6.0 Hz), 7.74 (d, 2H, Ar-*H*, *J* = 8.0 Hz), 7.66 (d, 1H, Ar-*H, J* = 8.0 Hz), 7.52 (d, 1H, Ar-*H, J* = 8.0 Hz), 7.38–7.28 (m, 10H, Ar-*H* + N*H_2_*), 7.18 (bs, 1H, Ar-*H*), 7.09 (t, 1H, Ar-*H, J* = 8.0 Hz), 6.99 (t, 1H, Ar-*H, J* = 6.0 Hz), 4.98 (s, 2H, C*H_2_*OCO), 4.37–4.32 (m, 3H, OCONHC*H*CONHC*H_2_*), 3.18–3.13 (m, 1H, (3-Indolyl)C*H_2_*CH), 3.00–2.94 (m, 1H, (3-Indolyl)C*H_2_*CH).

^13^C NMR (100 MHz, DMSO-d_6_): δ 172.5 (*C*ONHCH_2_), 156.4 (CH_2_O*C*O), 144.0, 143.0, 137.5, 136.6, 128.8, 128.2, 128.0, 127.8, 127.7, 126.1, 124.4, 121.4, 119.0, 118.7, 111.8, 110.6 (Ar-*C*), 65.8 (*C*H_2_OCO), 56.2 (OCONH*C*H), 42.3 (CONH*C*H_2_), 28.3 ((3-Indolyl)*C*H_2_CH). _ν_(C–O)carbamate: 1656 cm^−1^, _ν_(C–O)amide: 1682 cm^−1^, _ν_(N-H)amine: 3311, 3681 cm^−1^. Anal. calculated for C_26_H_26_N_4_O_5_S: C, 61.65; H, 5.17; N, 11.06; S, 6.33. Found: C, 61.85; H, 5.20; N, 11.03; S, 5.28. HRMS *m/z* for C_26_H_26_N_4_O_5_S [M + H]^+^ calcd. 507.2, found 507.3; [M-H]^−^ calcd. 505.2, found 505.2.

#### 2.3.12. tert-Butyl ((2R,3R)-3-methyl-1-oxo-1-((4-sulphamoylbenzyl)amino)pentan-2-yl)carbamate (24)

White solid (80%); mp 180–181 °C; ^1^H-NMR (400 MHz, DMSO-d_6_): δ 8.49 (t, 1H, N*H*CH_2_CH_2_, *J* = 6.0 Hz), 7.74 (d, 2H, Ar-*H*, *J* = 8.0 Hz), 7.43 (d, 2H, Ar-*H*, *J* = 8.0 Hz), 7.32 (s, 2H, N*H_2_*), 6.81 (d, 1H, OCON*H*CH_,_ *J* = 8.0 Hz), 4.35 (t, 2H, CONHC*H_2_, J* = 4.0 Hz), 3.81 (t, 1H, OCONHC*H, J* = 8.0 Hz), 1.73–1.69 (m, 1H, CHC*H*(CH_3_)CH_2_CH_3_), 1.41 (s, 10H, OC(C*H_3_*)*_3_* + CHCH(CH_3_)C*H_2_*CH_3_), 1.18–1.05 (m, 1H, CHCH(CH_3_)C*H_2_*CH_3_), 0.84–0.80 (m, 6H, CHCH(C*H_3_*)CH_2_C*H_3_*).

^13^C NMR (100 MHz, DMSO-d_6_): δ 172.3 (*C*ONHCH_2_), 156.0 ((CH_3_)_3_CO*C*O), 144.1, 143.0, 127.8, 126.0 (Ar-*C*), 78.5 ((CH_3_)_3_*C*OCO), 59.5 (OCONH*C*H), 42.1 (CONH*C*H_2_), 36.5 (CHC*H*(CH_3_)CH_2_CH_3_), 28.7 (OC(C*H_3_*)*_3_*), 25.0 (CHCH(CH_3_)*C*H_2_CH_3_), 15.9 (CHCH(*C*H_3_)CH_2_CH_3_), 11.4 (CHCH(C*H_3_*)CH_2_*C*H_3_). _ν_(C–O)carbamate: 1660 cm^−1^, _ν_(C–O)amide: 1678 cm^−1^, _ν_(N-H)amine: 3265, 3326, 3681 cm^−1^. Anal. calculated for C_18_H_29_N_3_O_5_S: C, 54.12; H, 7.32; N, 10.52; S, 8.02. Found: C, 53.77; H, 7.24; N, 10.75; S, 7.28. HRMS *m/z* for C_18_H_29_N_3_O_5_S [M + Na]^+^ calcd. 422.2, found 422.3; [M-H]^−^ calcd. 398.2, found 398.1; [M + HCOO]^−^ calcd. 444.2, found 444.2.

### 2.4. CA inhibition

An Applied Photophysics Stopped-Flow instrument has been used for assaying the CA catalysed CO_2_ hydration activity by using method of Khalifah^25^. Phenol red (at a concentration of 0.2 mM) has been used as indicator, working at the absorbance maximum of 557 nm, with 20 mM HEPES (pH 7.5) as buffer, and 20 mM Na_2_SO_4_ (for maintaining constant the ionic strength), following the initial rates of the CA-catalysed CO_2_ hydration reaction for a period of 10–100 s. The CO_2_ concentrations ranged from 1.7 to 17 mM for the determination of the kinetic parameters and inhibition constants. For each inhibitor at least six traces of the initial 5–10% of the reaction have been used for determining the initial velocity. The uncatalyzed rates were determined in the same manner and subtracted from the total observed rates. Stock solutions of inhibitor (0.1 mM) were prepared in distilled–deionised water and dilutions up to 0.01 nM were done thereafter with the assay buffer. Inhibitor and enzyme solutions were pre-incubated together for 15 min at room temperature prior to assay, in order to allow for the formation of the E-I complex. The inhibition constants were obtained by non-linear least-square methods using PRISM (www.graphpad.com), and non-linear least squares methods, values representing the mean of at least three different determinations, as described earlier by us^26–30^.

## 3. Results and discussion

### 3.1. Synthesis and characterisation of the new amino acid–sulphonamides conjugates

**Scheme 1.**
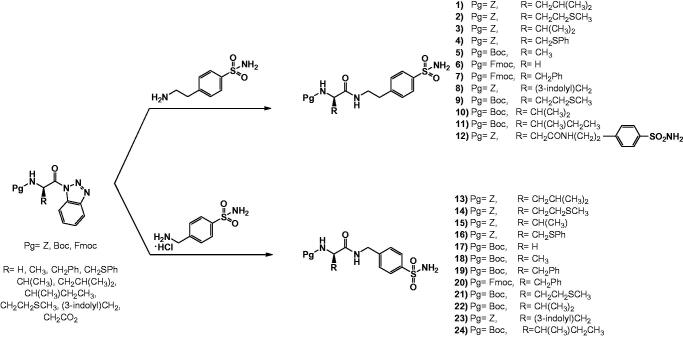
Synthetic pathways of N-protected peptide-benzimidazole conjugates, **1**–**24**.

This work was designed to synthesise, characterise, and explore the potential CA inhibitory properties of N-protected amino acid–sulphonamide conjugates. The syntheses of novel homosulphonamide and 4-aminoethylsulphonamide–amino acid conjugates prepared in this study are shown in Scheme 1. Since *N*-acylbenzotriazoles have been successfully utilised in many acylation reaction for the preparation N-, O-, S-, and C- substituted amino acid or peptides^31–40^, we chose the benzotriazole-mediated methodology to synthesise the targeted amino acid**–**sulphonamide conjugates. Compounds **1–12** were prepared through a facile benzotriazole mediated acylation reactions in one step (Scheme 1) at 70 °C under microwave irradiation for 30 min, in dry dichloromethane as solvent, whereas compounds **13**–**24** were prepared using the same method except the presence of triethylamine in the reaction mixture, in order to remove hydrogen chloride from the homosulphonamide . HCl reagent.

All compounds were fully characterised by ^1^H, ^13^C NMR, MS, and FTIR (ATR) spectroscopy and elemental analyses. The analytical and spectral data of all the synthesised compounds were in full agreement with the proposed structures. The characteristic NH resonances of the sulphonamide part of the amino acid conjugates **1**–**12** were observed at 7.88–8.25 ppm region as triplet peak whereas compounds **13**–**24** were observed at 8.41–8.76 ppm region as triplet peak in the ^1^H NMR spectrum. NH_2_ resonances of sulphonamide part were observed generally in the aromatic region together with aromatic protons. These NH or NH_2_ protons were confirmed by D_2_O exchange. The singlet that peak around 5.00 ppm for compounds **1**–**4**, **8**, **12**, **13**–**16,** and **23** was assigned to the CH_2_ protons for benzyloxycarbonyl protected group whereas the upfield singlet that signals around 1.30 ppm was assigned to the tert-butyl protons of Boc-protected group for compounds **5**, **9**–**11**, **17**–**22,** and **24**. The ^1^H NMR spectra of compounds **13**–**24** revealed a doublet peak due to CH_2_ protons at 4.32–4.36 ppm. The multiplet peak around 4.20 ppm for compounds **6**, **7,** and **20,** those containing Fmoc protecting group, were assigned to the CH and CH_2_ protons for Fmoc group. Carbonyl resonances of the amide carbonyls and carbamate carbonyl were observed downfield around 170 and 155 ppm, respectively. All other aliphatic and aromatic protons and carbons were observed in the expected regions (see Materials and methods). The molecular ion or appropriate positive or negative ion peaks were observed for all proposed structures of novel compounds in the mass spectra. The IR spectra of amino-sulphonamide conjugates, **1**–**24**, showed characteristic amide carbonyl peaks around between 1671 and 1712 cm^−1^, whereas the carbamate carbonyl peaks around between 1634 and 1664 cm^−1^. All other spectral data were in accordance with the assumed structures.

### 3.2. Carbonic anhydrase inhibition

As can be seen from Table 1, some derivatives of 4-(2-aminoethyl) sulphonamide bearing leucine, methionine, valine, and glycine (**1**–**3**, **5,** and **6**) showed better inhibitory properties with K_I_ values ranging from 9.6 to 73.5 nM against hCA I than standard sulphonamide compound **AZA**. Four homosulphonamide derivatives bearing leucine, methionine, valine, and isoleucine (**13**–**15** and **24**) showed better inhibitory properties with K_I_ values ranging from 9.3 to 177.8 nM against hCA I than standard sulphonamide compound **AZA**. Among the sulphonamide derivatives, those carrying benzyloxycarbonyl (Z) protecting group generally showed a better inhibitory property against the hCA I compared to the corresponding deprotected derivatives. Most of the compounds exhibited inhibition constants K_I_ values ranging from 0.5 to 8.9 nM against hCA II, being more effective inhibitors than the standard sulphonamide acteazolamide **AZA**. In parallel to the results in hCA I, 4-(2-aminoethyl)benzenesulphonamide derivatives (**1**–**12**) showed better inhibition than the corresponding homosulphonamide derivatives (**13**–**24**). Compounds **1** and **3** also showed a better inhibition against hCA VA compared to **AAZ**, while compounds **2**, **4**–**6** showed a comparable inhibition to that of **AAZ**. Other compounds were found to be ineffective at the low nanomolar levels, being inhibitory only in the micromolar range. Two out of the twenty-four sulphonamide derivatives (compounds **8** and **21**) showed strong inhibitory properties against hCA XII, being more effective than the standard drug. Among these compounds, **3**, **4**, **8**, **17,** and **19** also showed comparable inhibitory properties against hCA XII to that of **AAZ**. The results of Table 1 show that alkyl and benzyloxycarbonyl protected groups contribute positively to sulphonamide conjugates inhibitory properties. This type of structure-activity relationship was observed for other structurally-related CAIs belonging to other chemotypes, when the tails of the amino acyl type or of other nature, effectively contributed to the inhibitory effects, due to favourable contacts made with the middle and external part of the active site^41–51^.

**Table 1.** Inhibition data of hCA I, hCA II, hCA VA, and hCA XII with compounds **1–24** and the standard sulphonamide inhibitor acetazolamide (**AAZ**) by a stopped flow CO_2_ hydrase assay.

K_I_ (nM)*				
Compound	hCA I	hCAI I	hCA VA	hCA XII
**1**	49.5	8.3	58.7	27.4
**2**	9.6	1.7	63.4	53.9
**3**	51.6	0.5	60.3	7.2
**4**	352.2	0.6	63.3	8.4
**5**	73.5	6.6	68.7	45.6
**6**	36.0	6.4	67.9	8.2
**7**	696.9	76.4	3442	38.9
**8**	528.0	6.5	3005	5.5
**9**	4390	0.9	610.6	329.4
**10**	2992	0.8	660.7	197.5
**11**	348.5	0.6	3870	56.2
**12**	689.4	215.5	3140	24.4
**13**	61.1	86.6	578.4	39.8
**14**	9.3	8.9	384.1	74.3
**15**	8.1	28.4	503.4	67.7
**16**	763.5	3.3	3961	37.1
**17**	3340	6.8	3653	8.8
**18**	3890	6.8	5360	20.6
**19**	3780	61.4	733.8	6.9
**20**	5175	693.2	6826	26.7
**21**	5356	315.6	4988	3.9
**22**	752.5	168.1	4779	90.1
**23**	305.1	0.9	401.2	78.1
**24**	177.8	0.8	4576	50.3
**AAZ**	250.0	12.1	63.0	5.7

^*^ Mean from three different assays, by a stopped flow technique (errors were in the range of ± 5–10% of the reported values).

## 4. Conclusions

In conclusion, novel sulphonamide derivatives containing amino acid moieties have been prepared in good to excellent yields, using a facile benzotriazole mediated reaction, by condensation of 4-amino-substituted sulphonamides with desired benzyloxycarbonyl protected amino acid derivatives. The CA inhibitory properties of the novel compounds were determined using a stopped flow instrument and four human CA isoforms with pharmacologic relevance. Most of the new sulphonamide–amino acid conjugates exhibited better inhibitory properties against hCA I and II than standard sulphonamide AAZ. Some compounds were also found to be efficient inhibitors against hCA VA and hCA XII, in nanomolar levels, with K_I_ values ranging from 58.7 to 68.7 nM and from 3.9 to 8.8 nM, respectively.

## Supplementary Material

Supplemental Material
