# Supplementary material for: Synthesis and human carbonic anhydrase I, II, VA, and XII inhibition with novel amino acid–sulphonamide conjugates
Source: J Enzyme Inhib Med Chem. 2020 Jan 8;35(1):489–97. doi: 10.1080/14756366.2019.1710503 (PMC6968503; doi:10.1080/14756366.2019.1710503)

Supporting Information

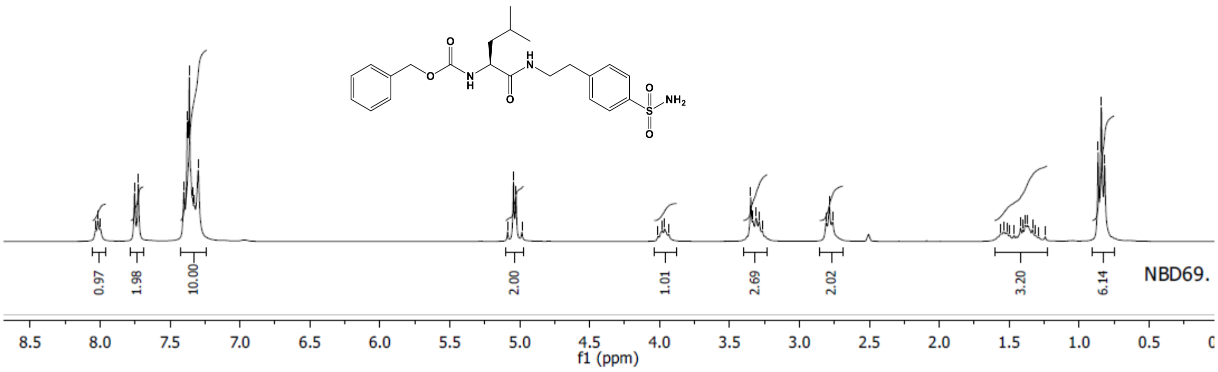

^{1}H spectrum of **1**

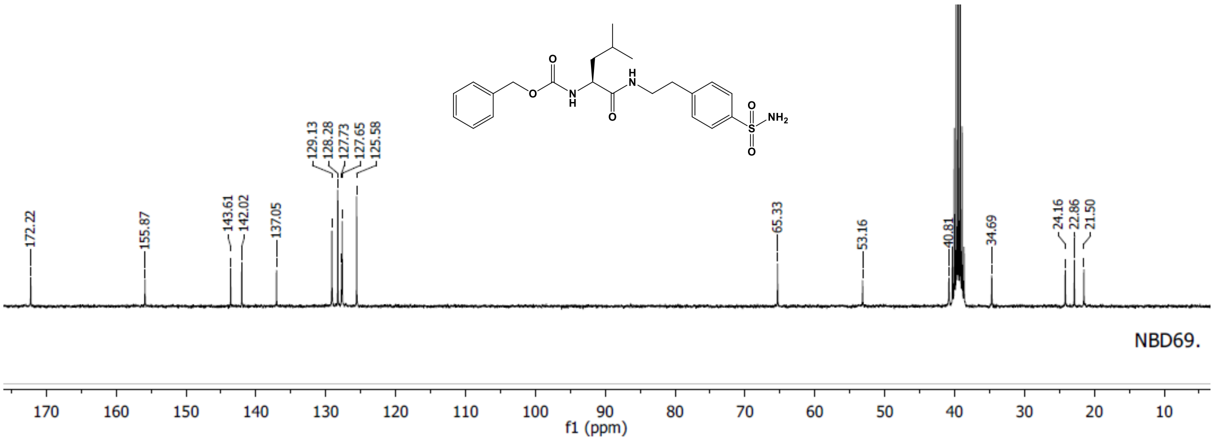

^{13}C spectrum of **1**

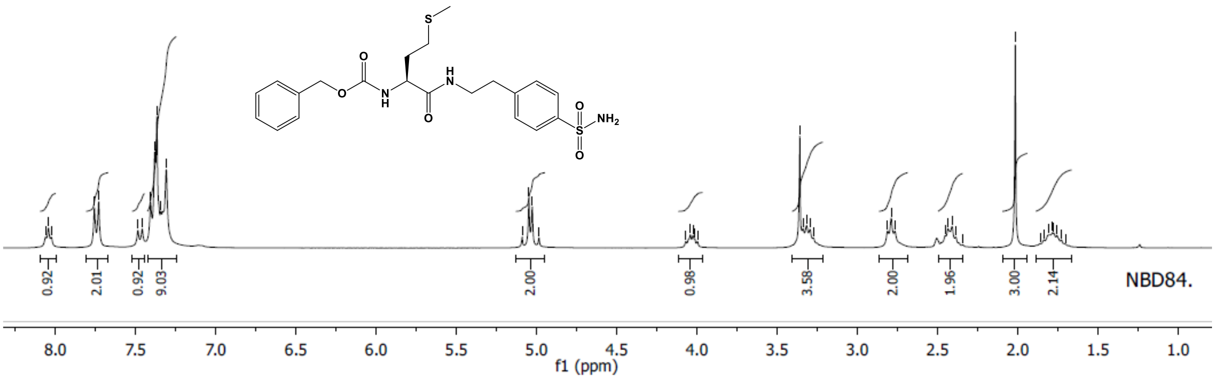

^{1}H spectrum of **2**

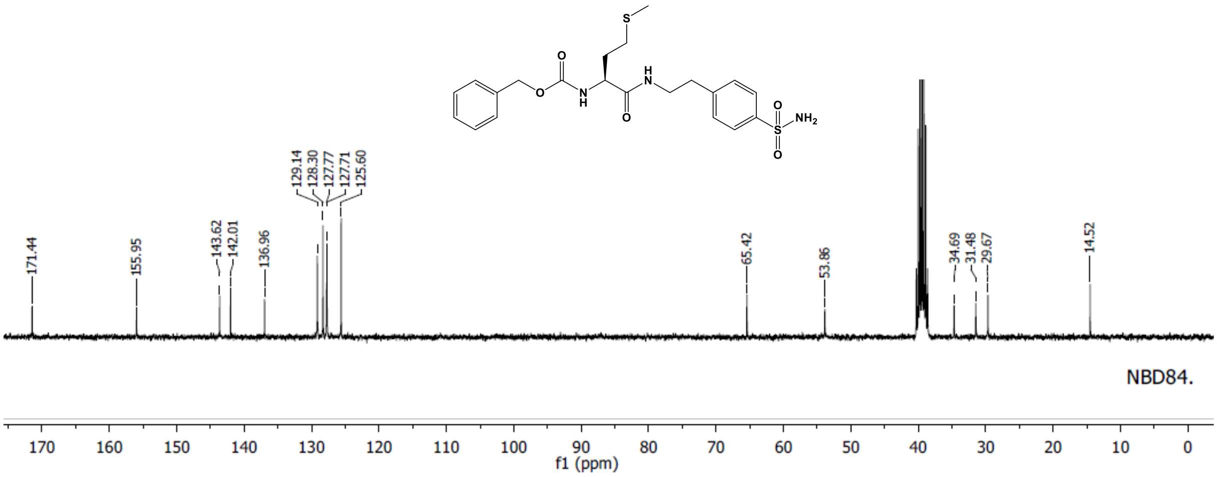

^{13}C spectrum of **2**

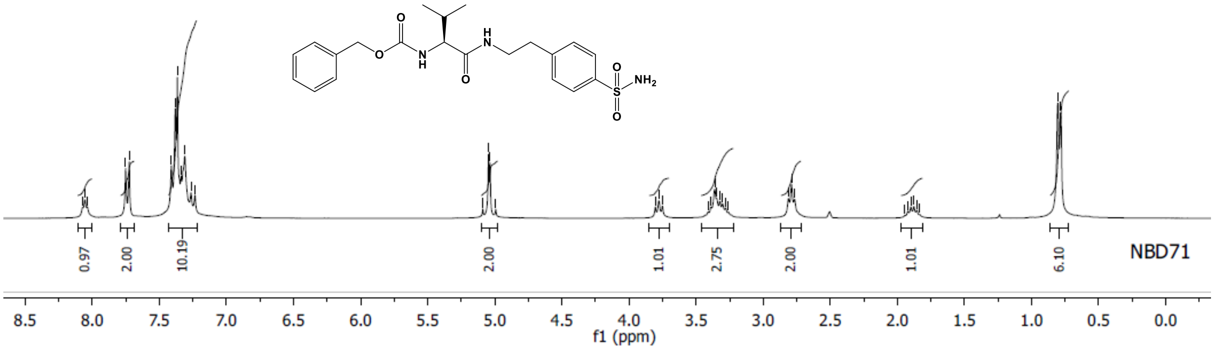

^{1}H spectrum of **3**

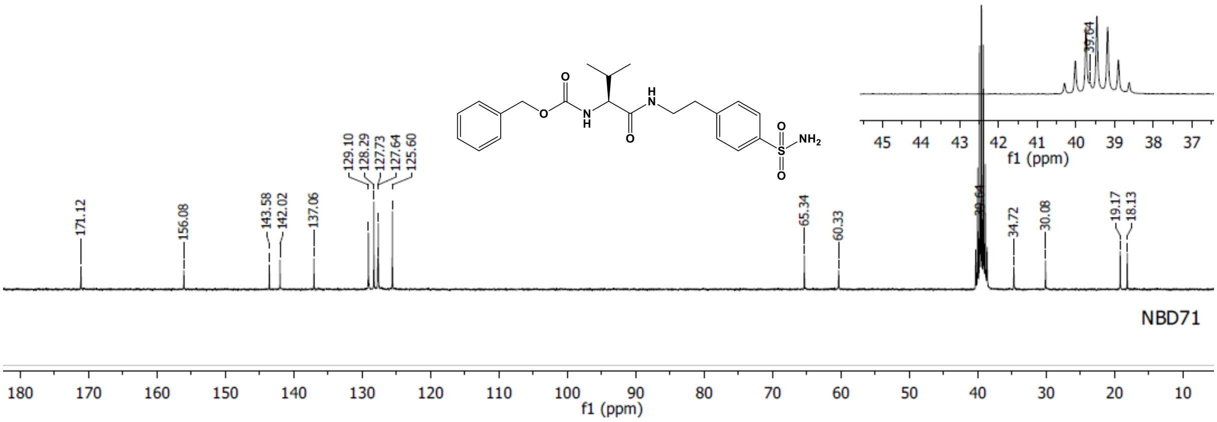

^{13}C spectrum of **3**

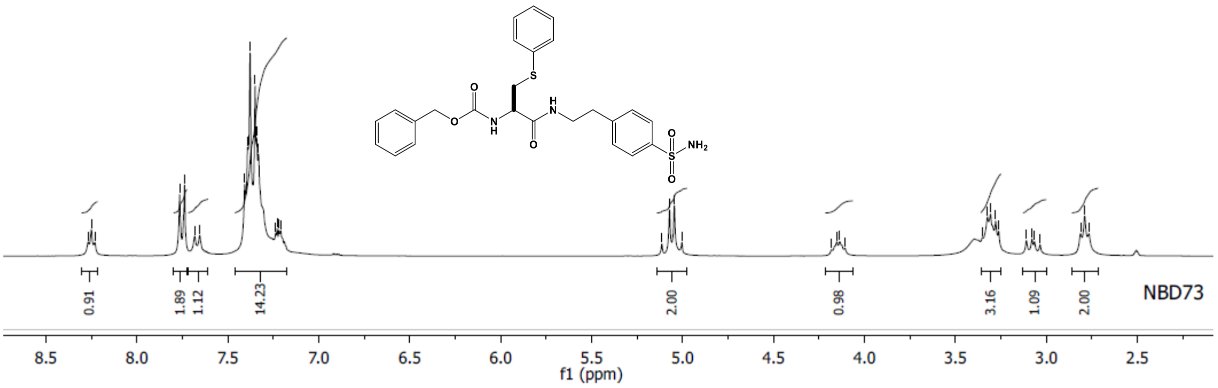

^{1}H spectrum of **4**

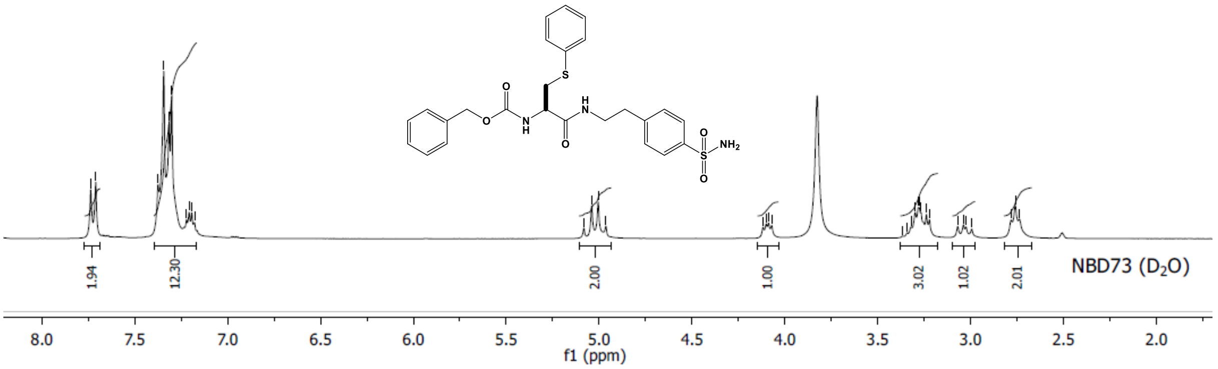

^{1}H (D_2O) spectrum of **4**

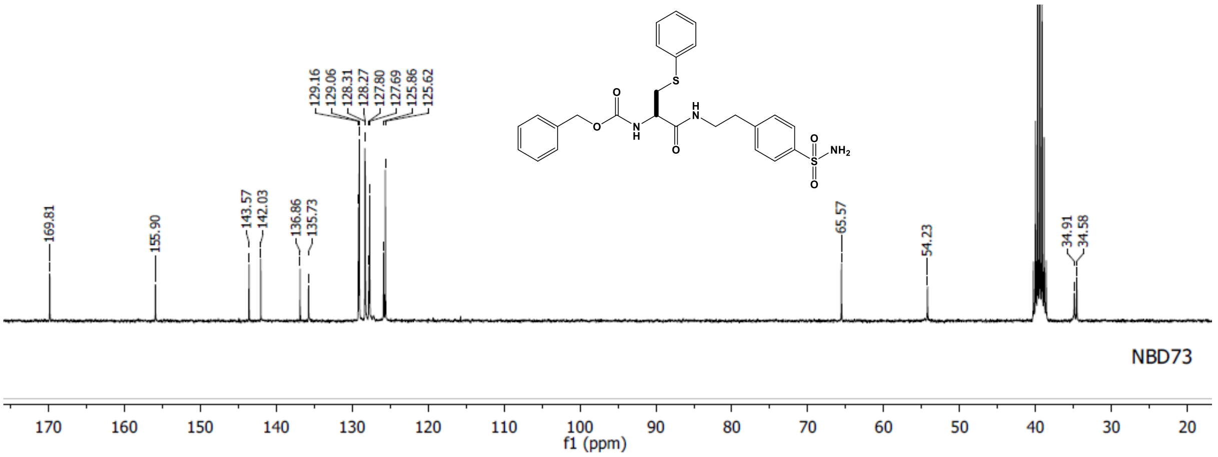

^{13}C spectrum of **4**

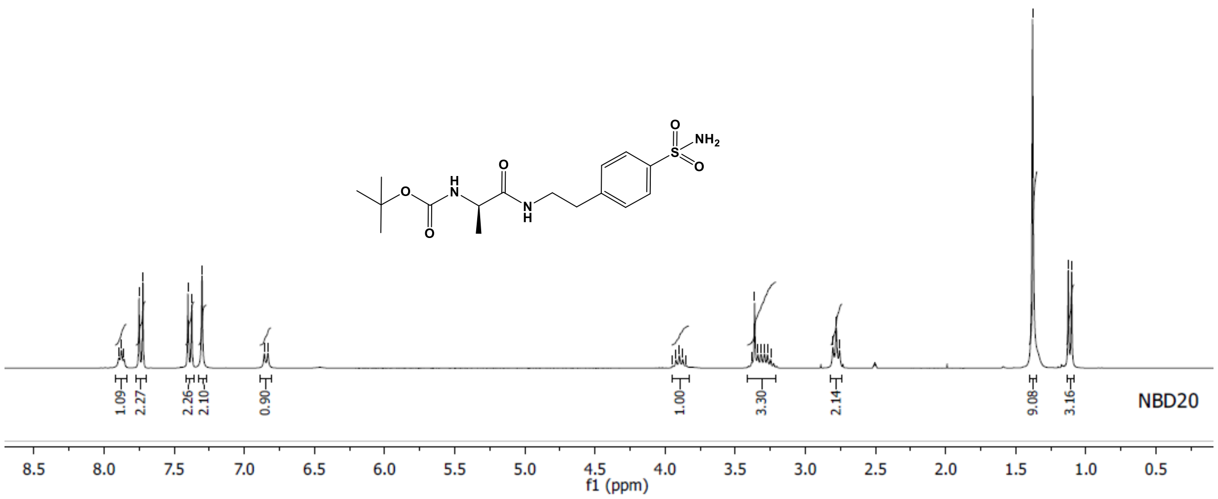

^{1}H spectrum of **5**

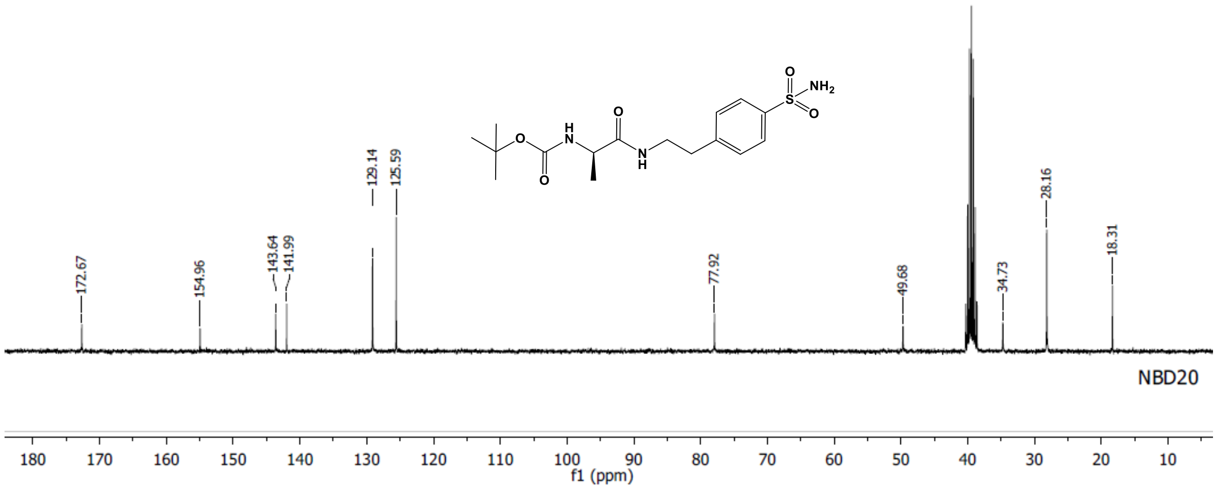

^{13}C spectrum of **5**

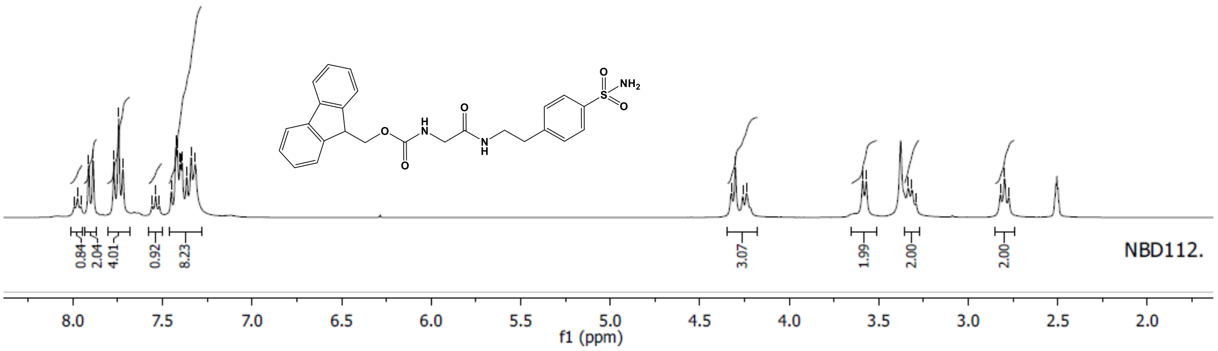

^{1}H spectrum of **6**

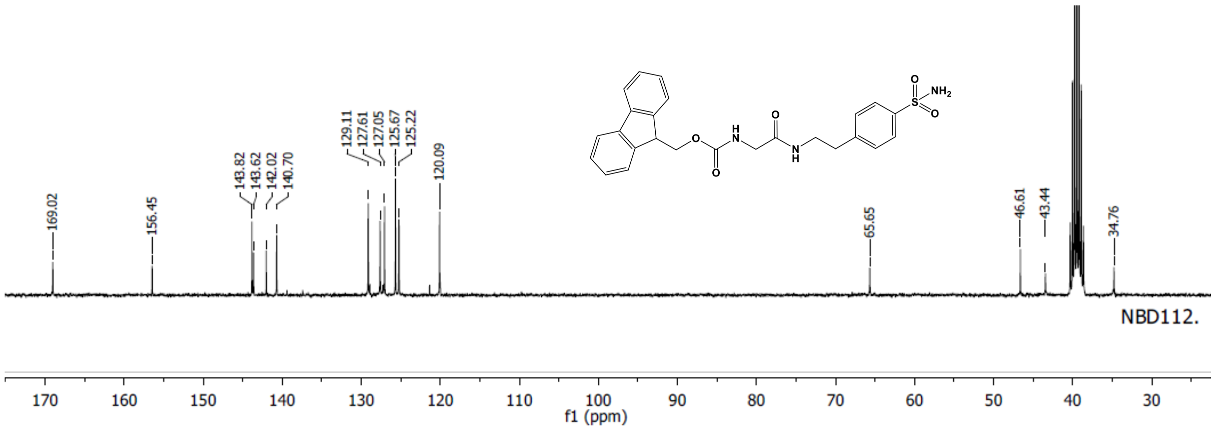

^{13}C spectrum of **6**

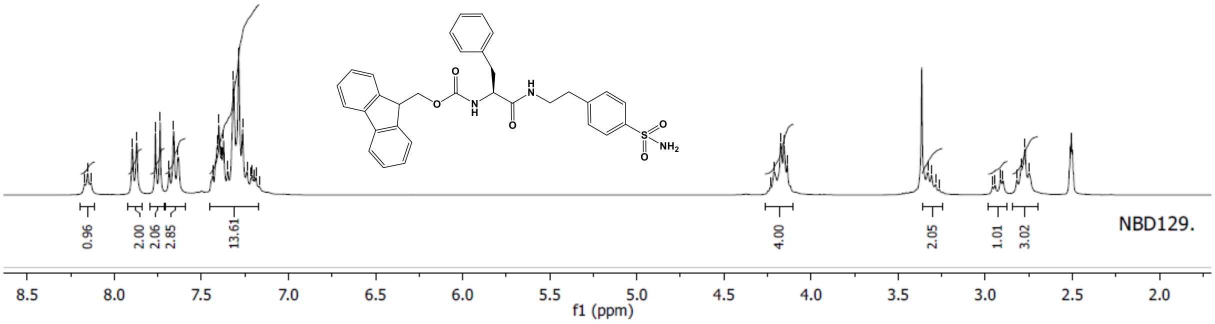

^{1}H spectrum of **7**

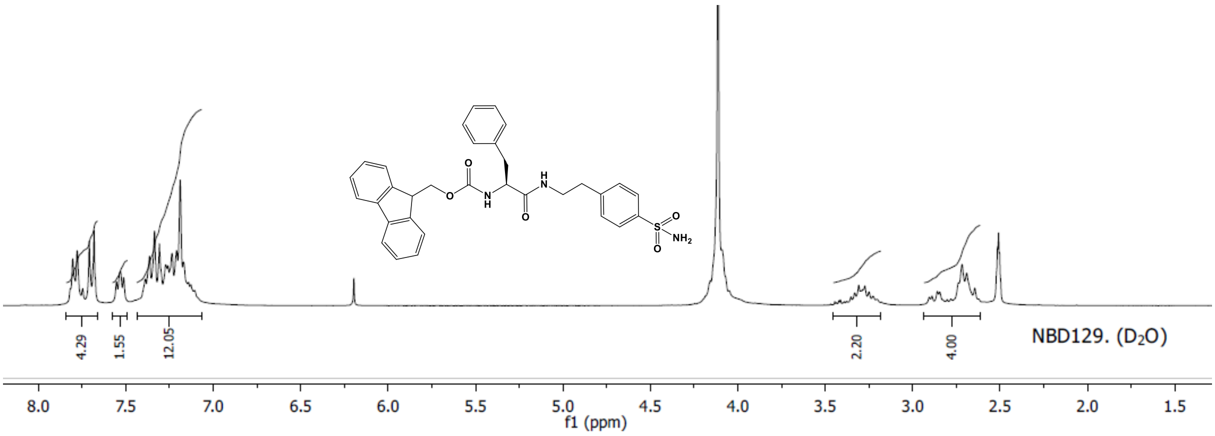

^{1}H (D_2O) spectrum of **7**

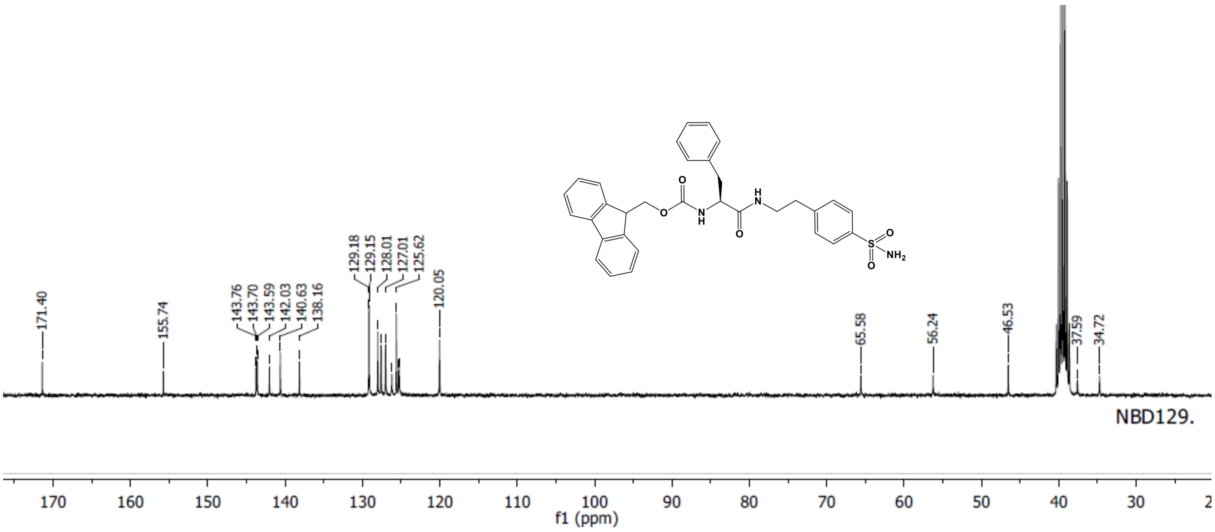

^{13}C spectrum of **7**

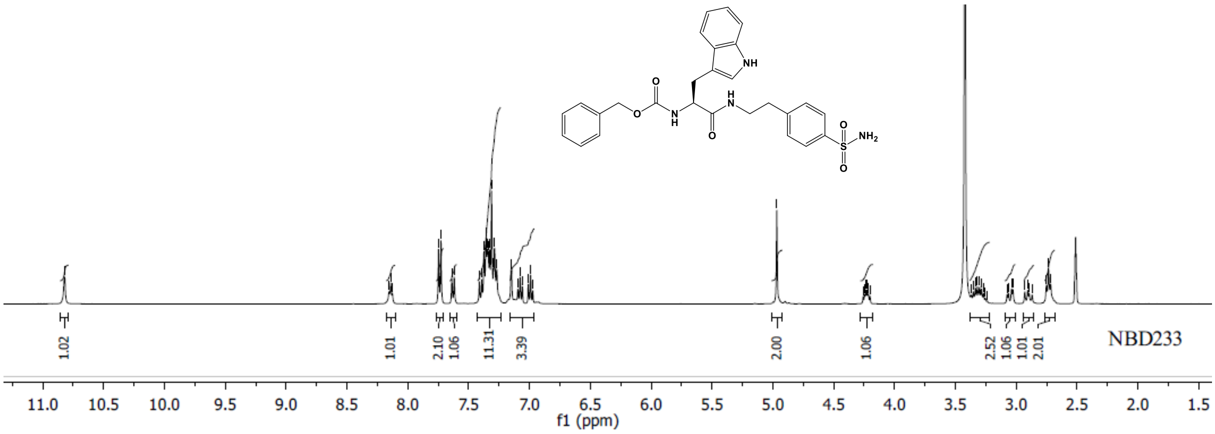

^{1}H spectrum of **8**

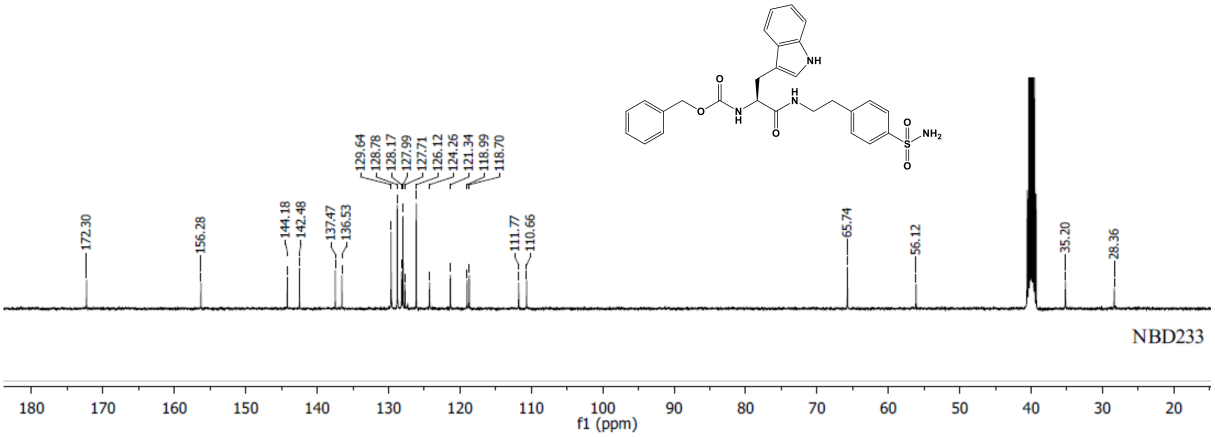

^{13}C spectrum of **8**

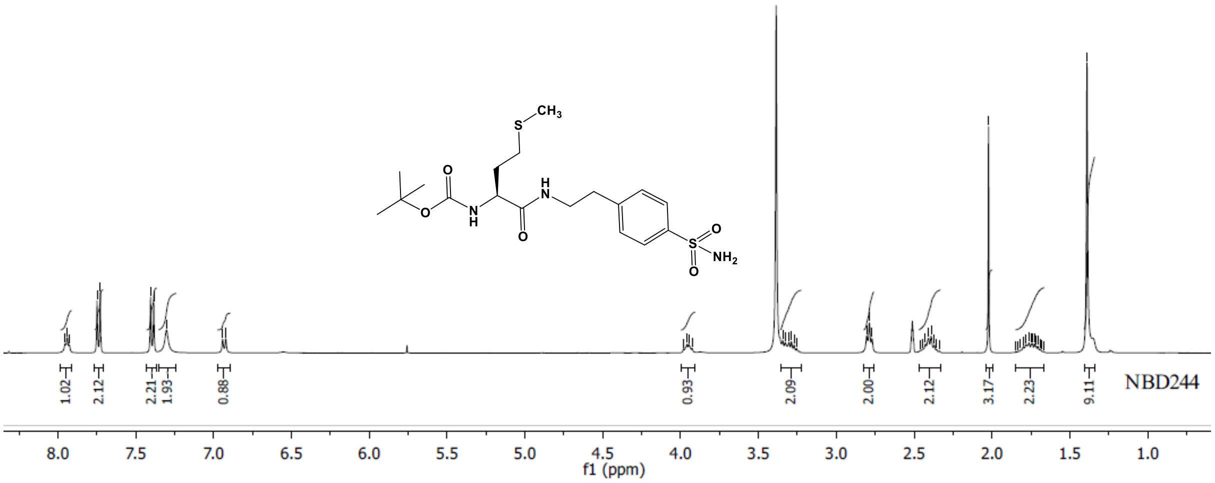

^{1}H spectrum of **9**

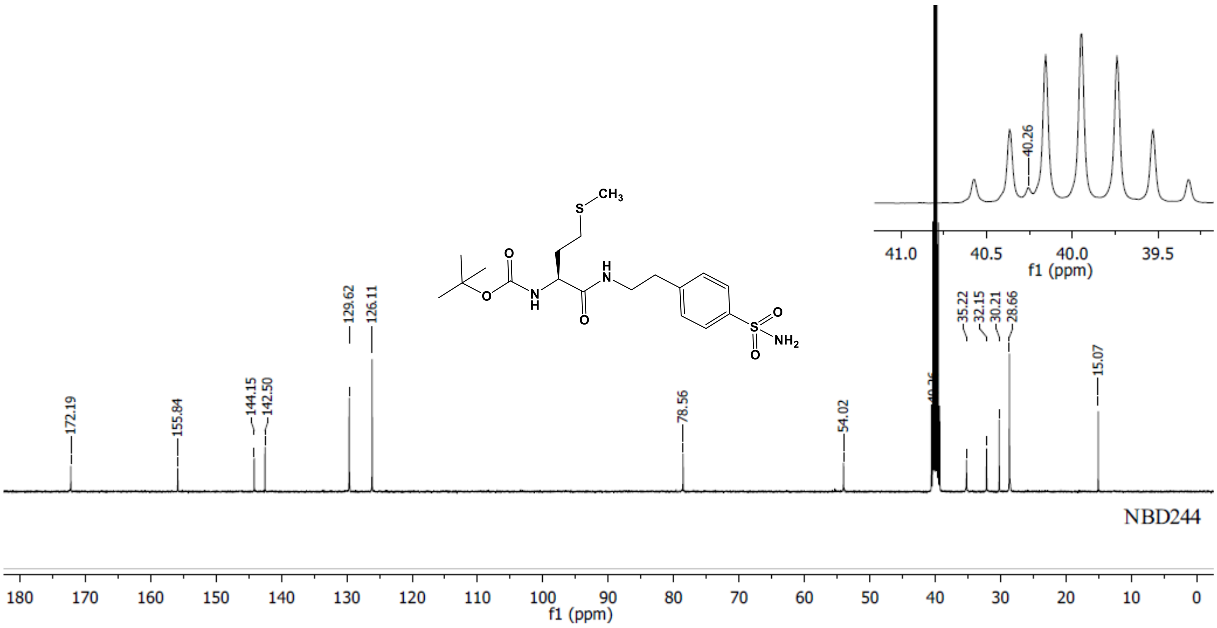

^{13}C spectrum of **9**

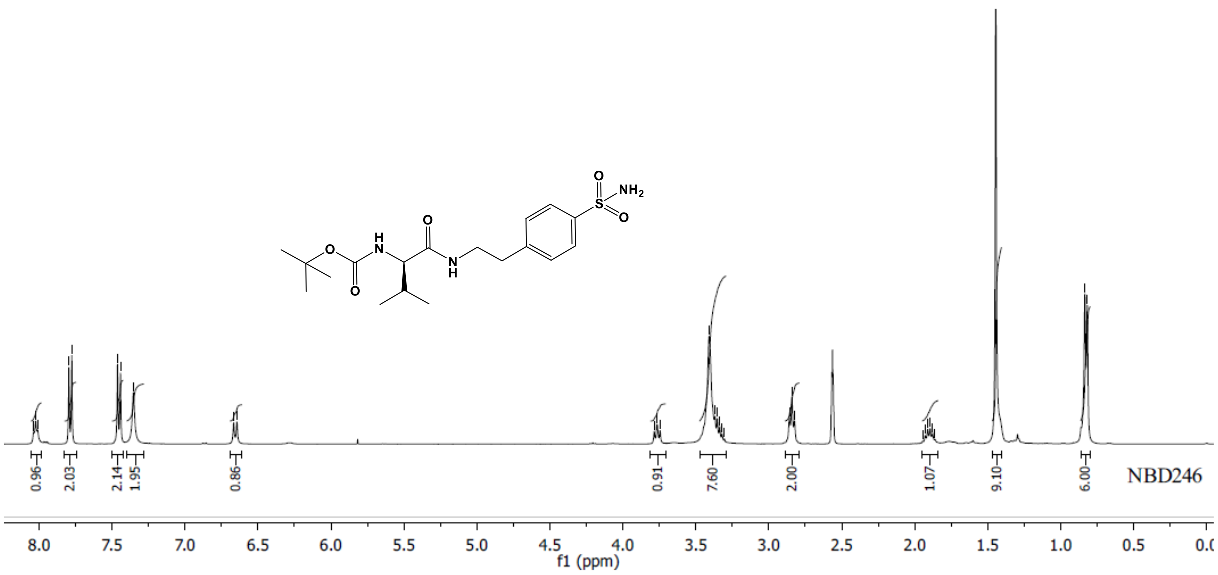

^{1}H spectrum of **10**

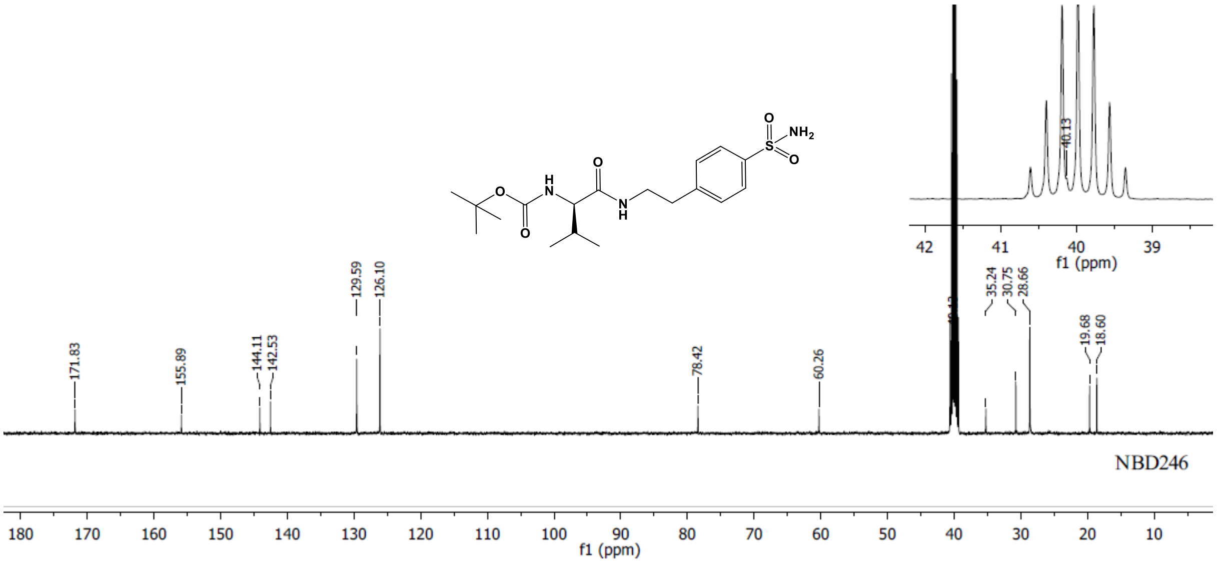

^{13}C spectrum of **10**

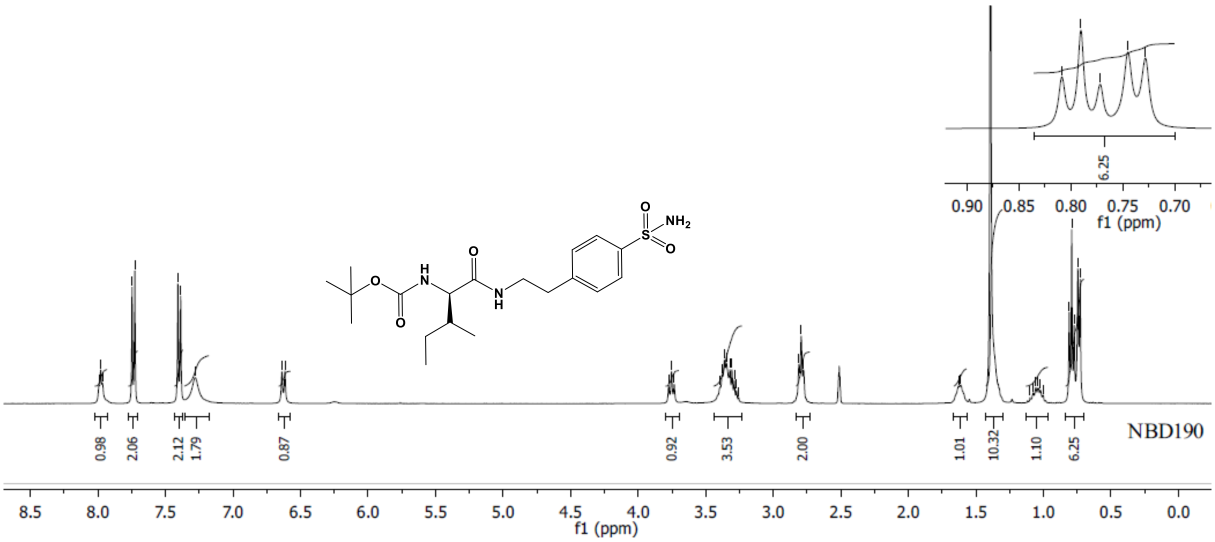

^{1}H spectrum of **11**

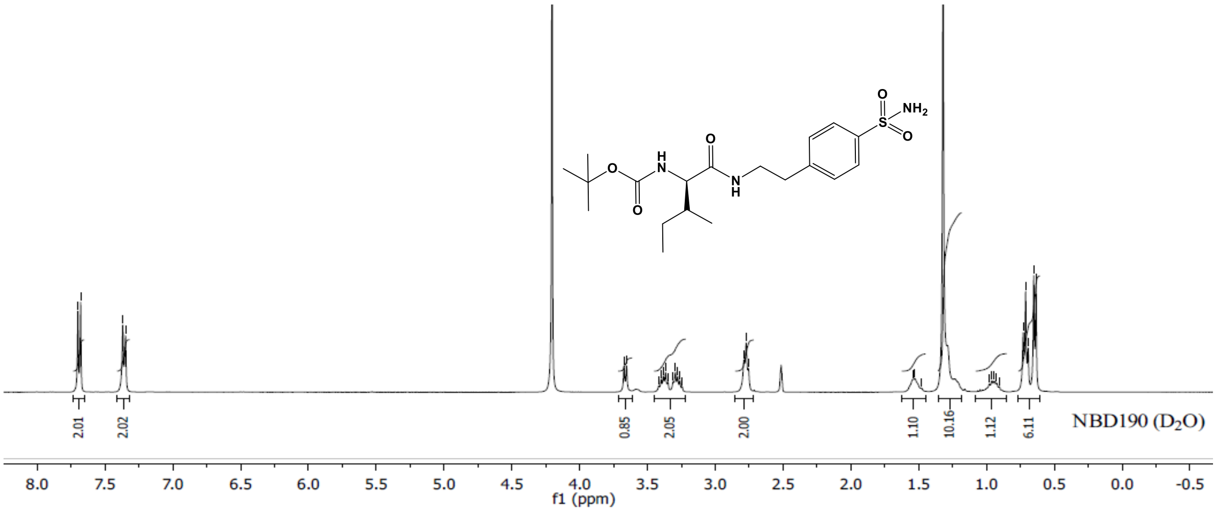

^{1}H (D_2O) spectrum of **11**

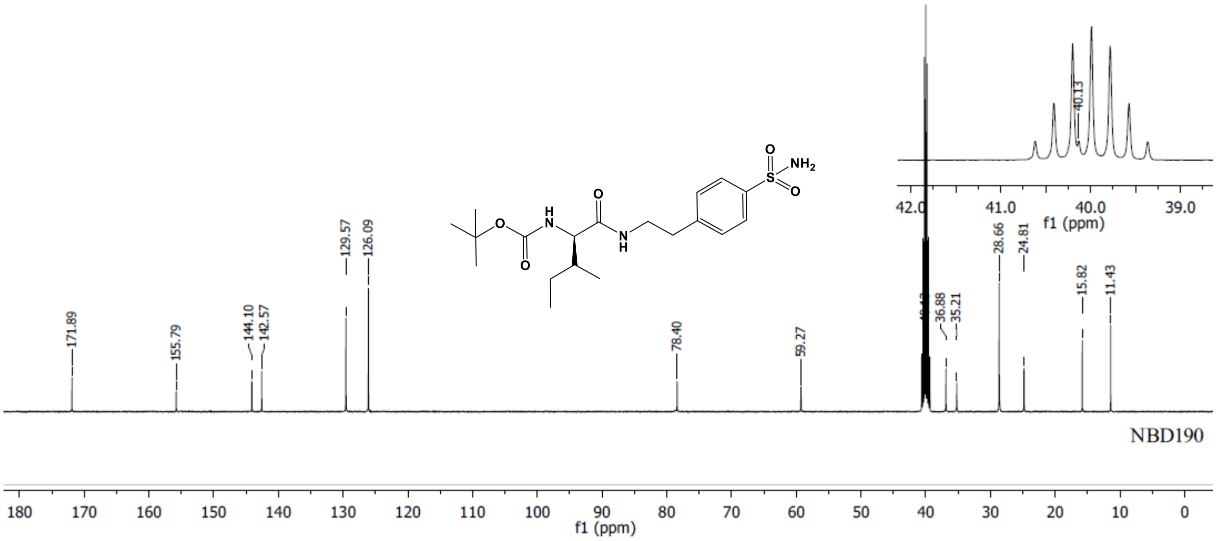

^{13}C spectrum of **11**

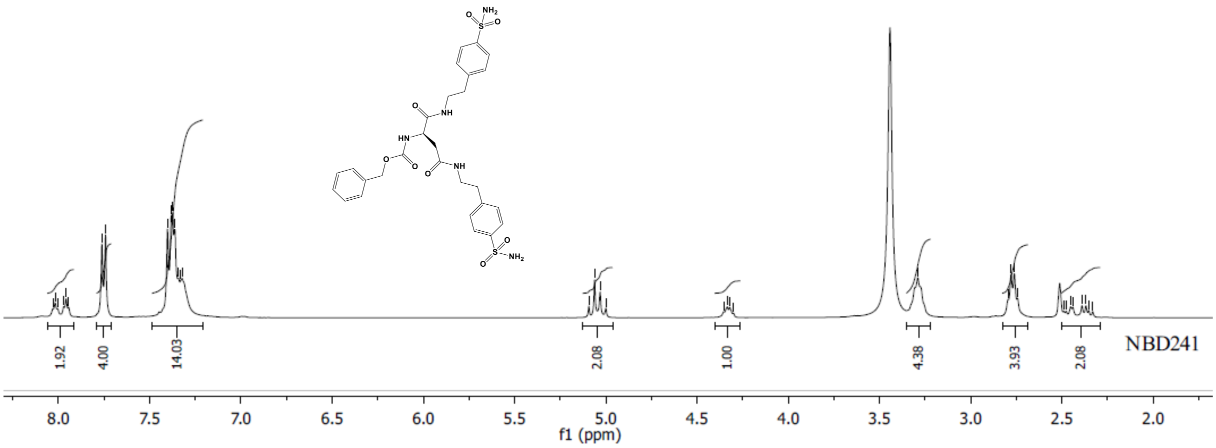

^{1}H spectrum of **12**

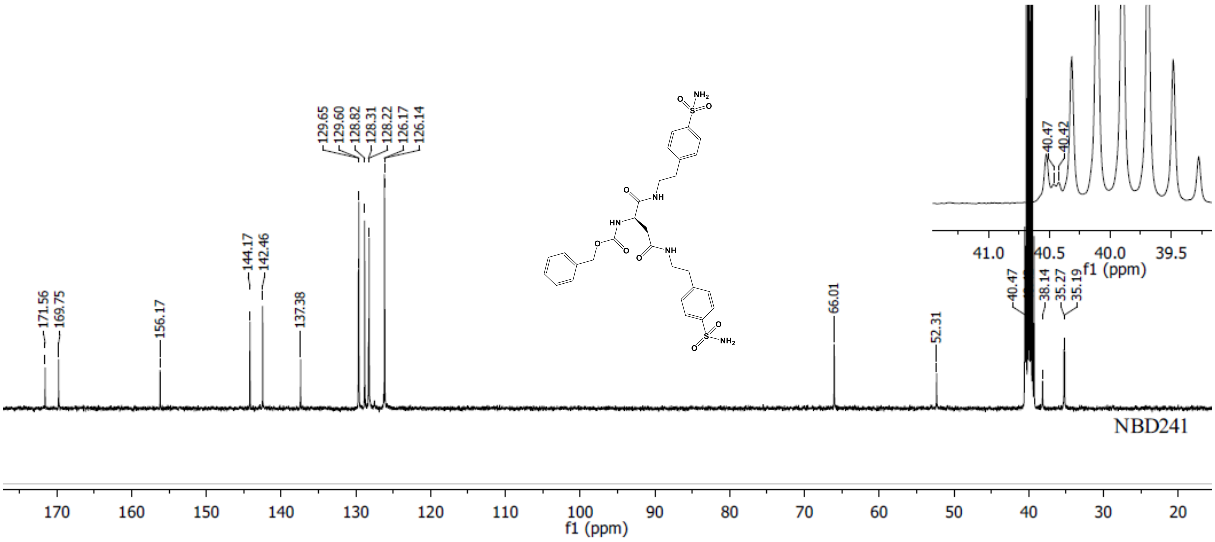

^{13}C spectrum of **12**

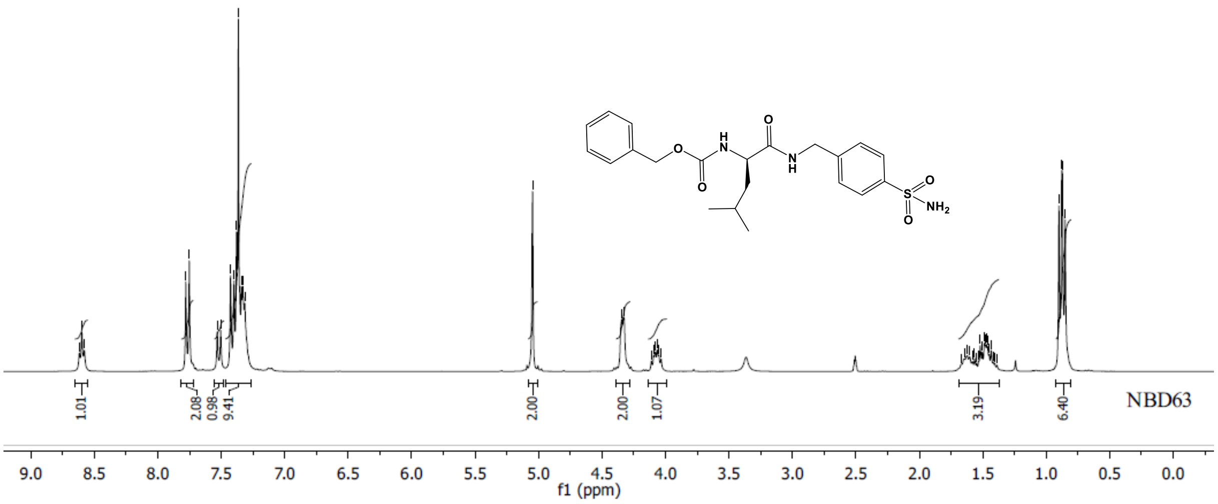

^{1}H spectrum of **13**

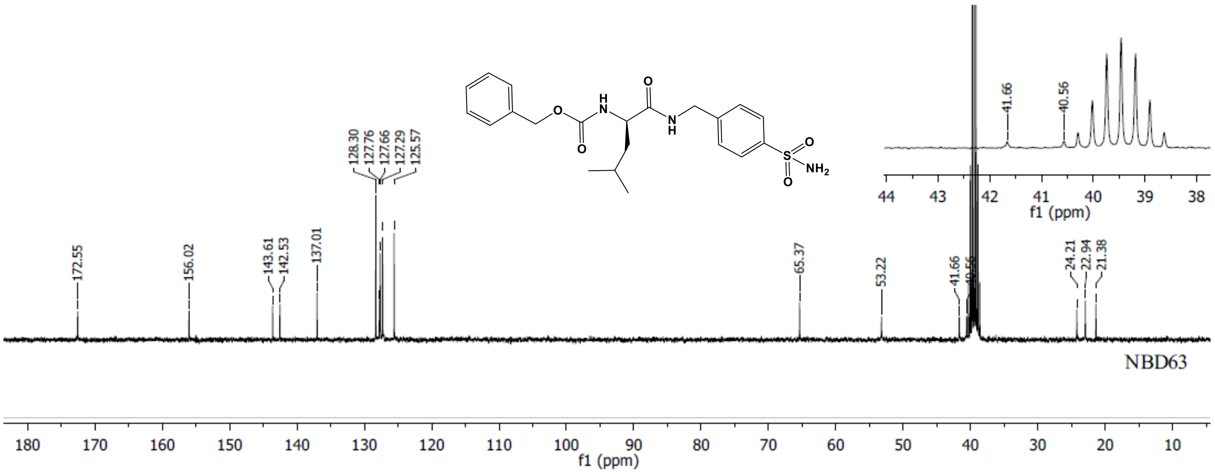

^{13}C spectrum of **13**

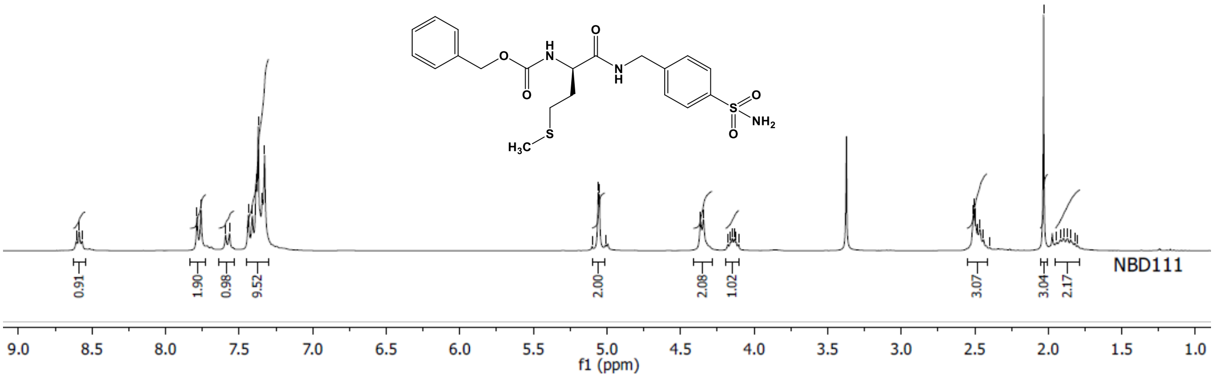

^{1}H spectrum of **14**

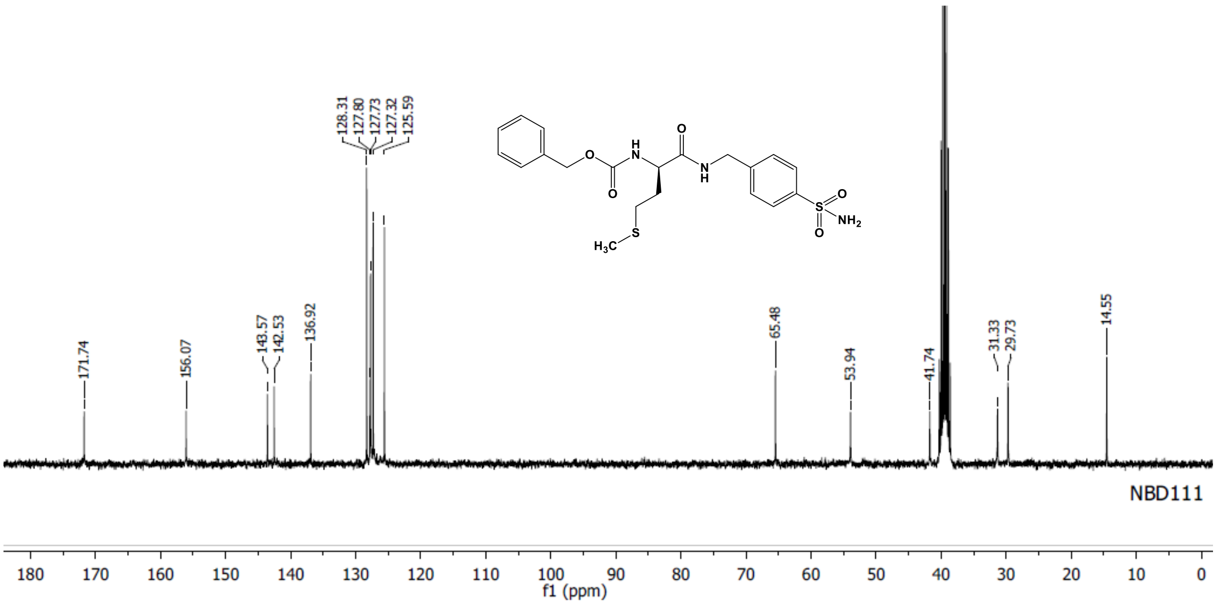

^{13}C spectrum of **14**

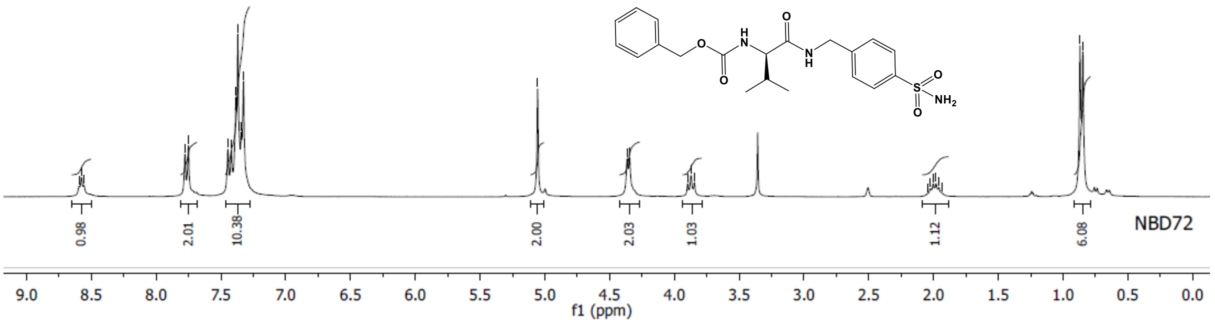

^{1}H spectrum of **15**

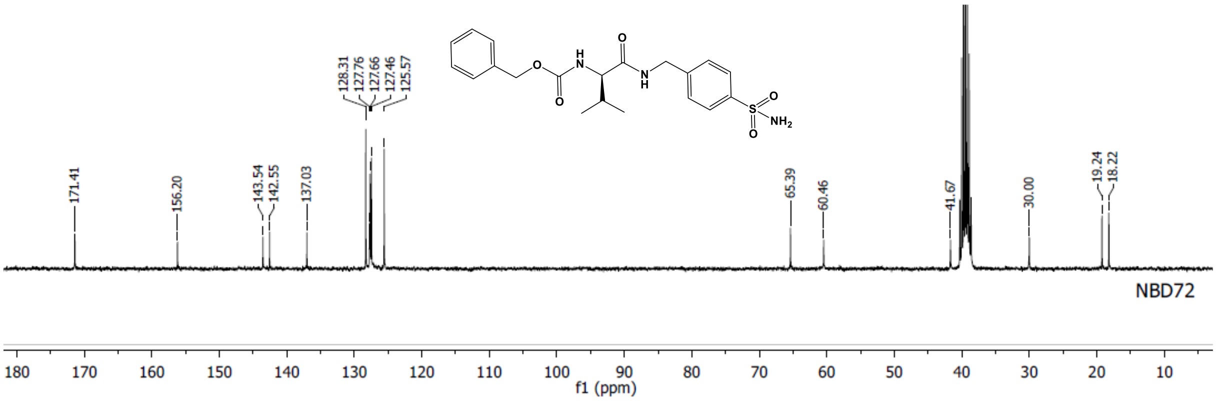

^{13}C spectrum of **15**

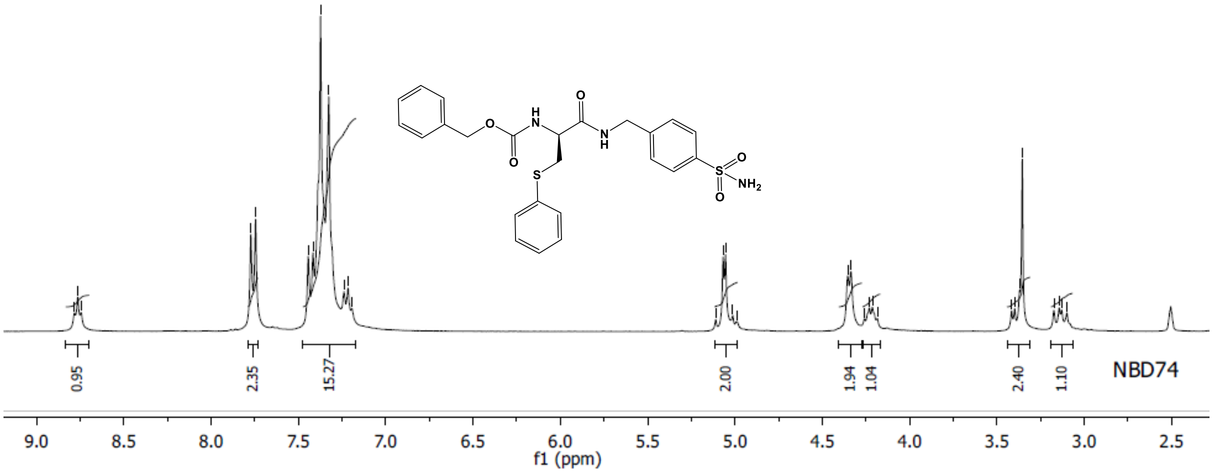

^{1}H spectrum of **16**

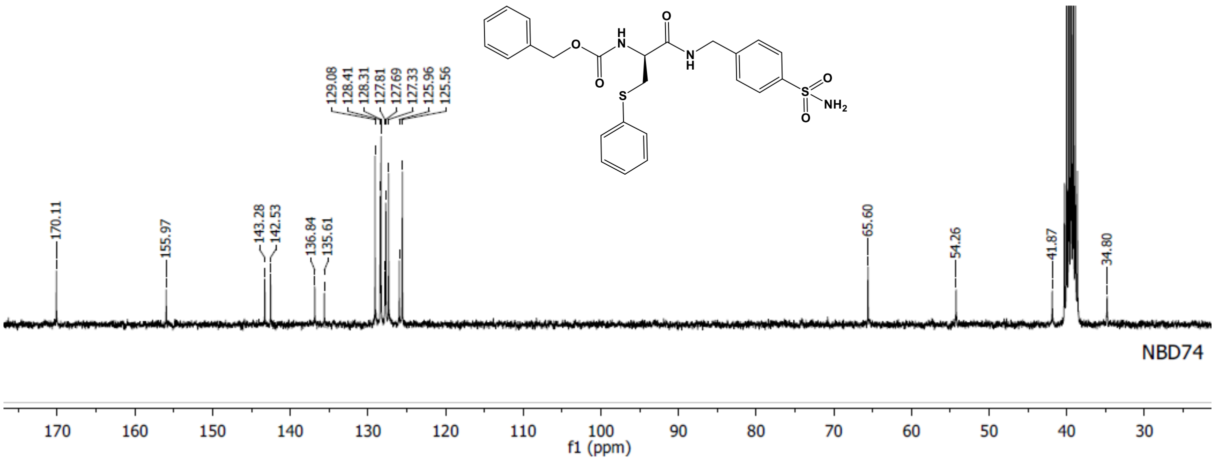

^{13}C spectrum of **16**

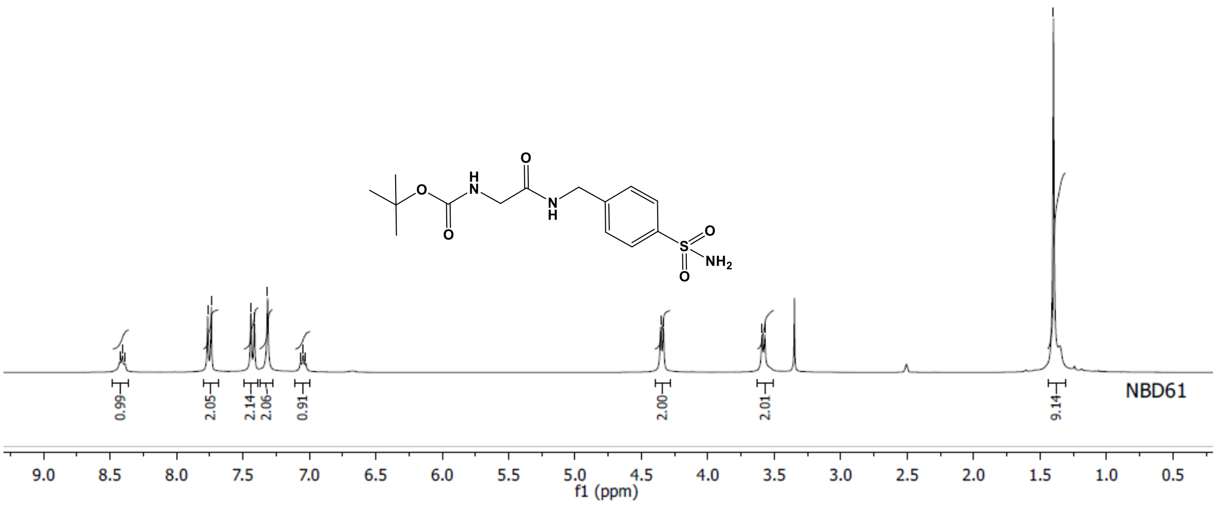

^{1}H spectrum of **17**

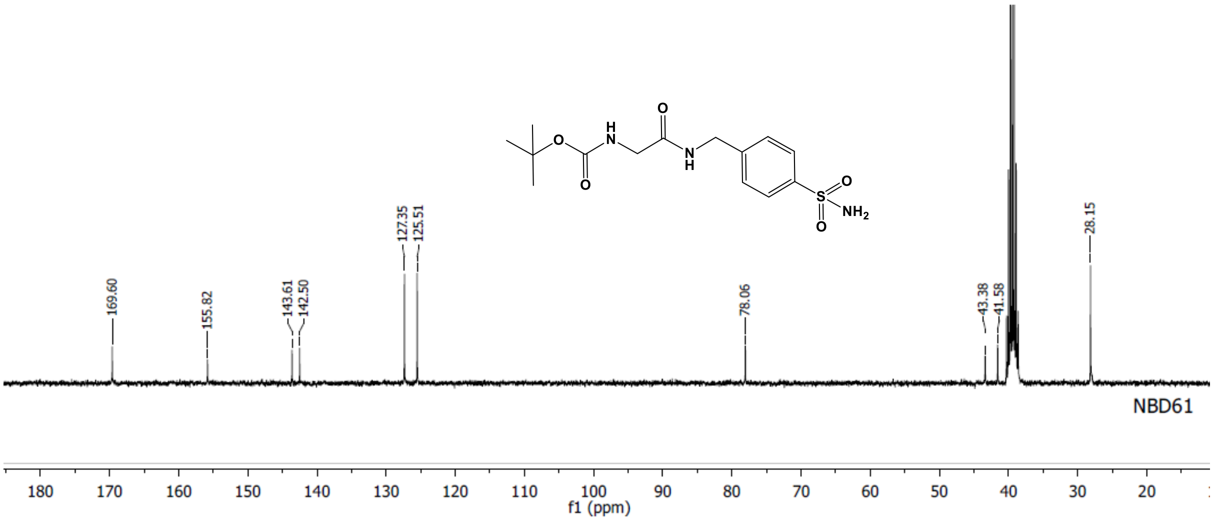

^{13}C spectrum of **17**

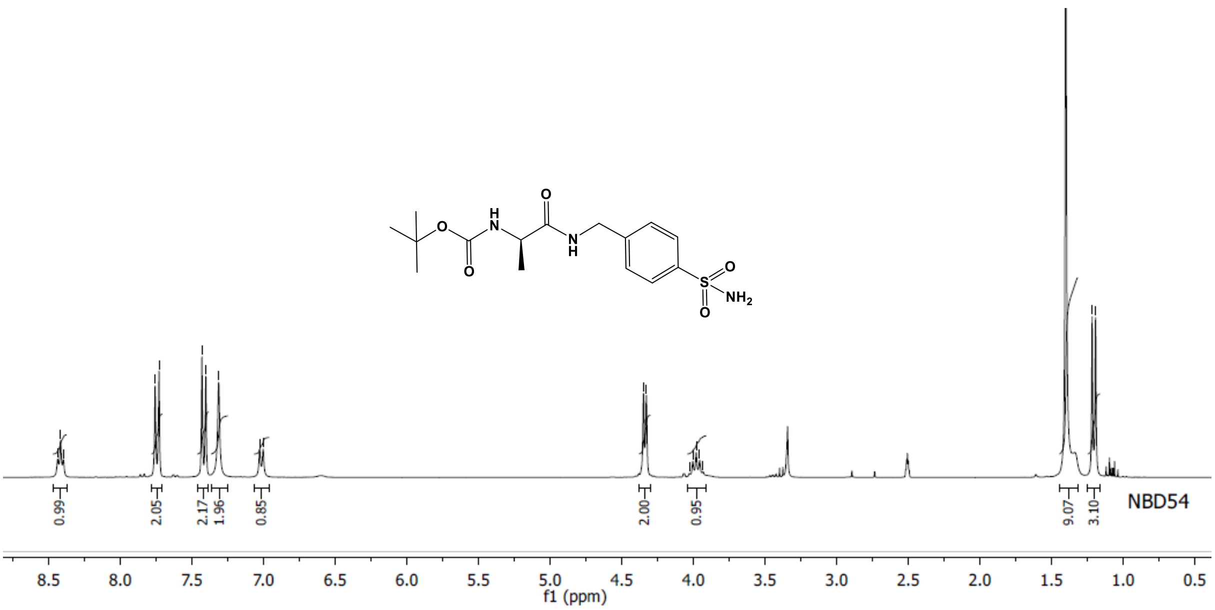

^{1}H spectrum of **18**

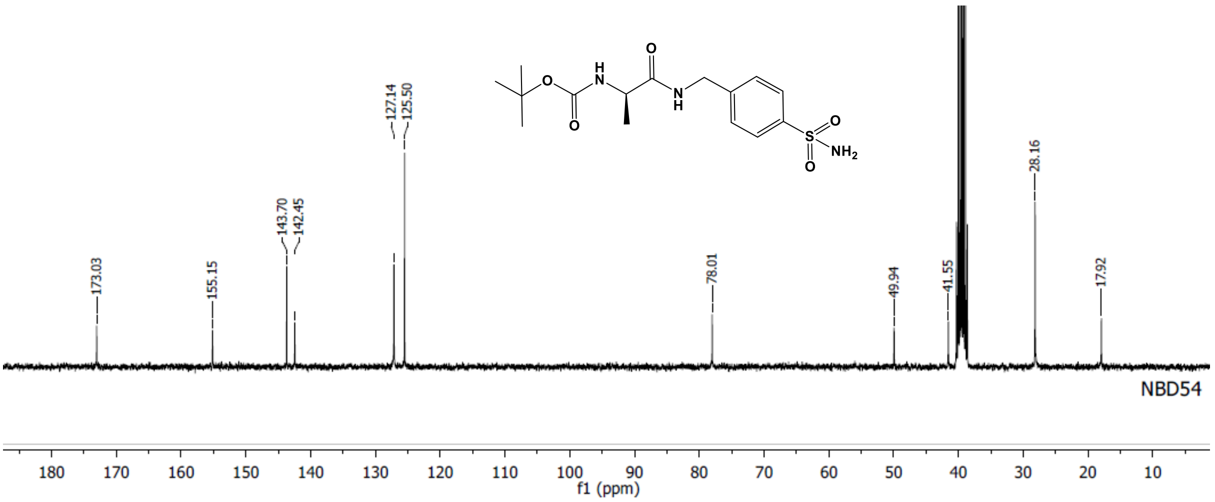

^{13}C spectrum of **18**

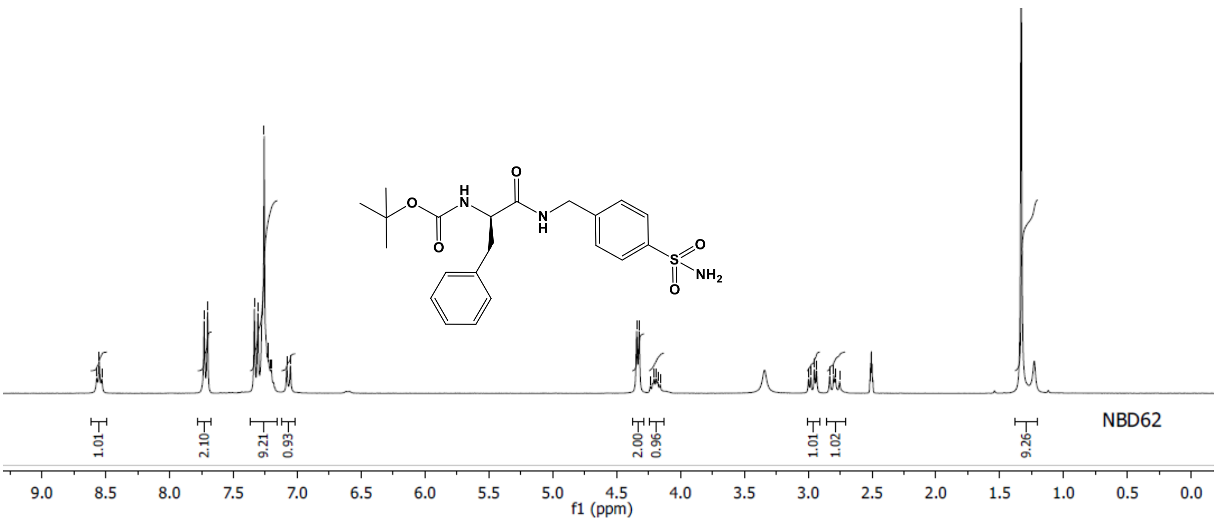

^{1}H spectrum of **19**

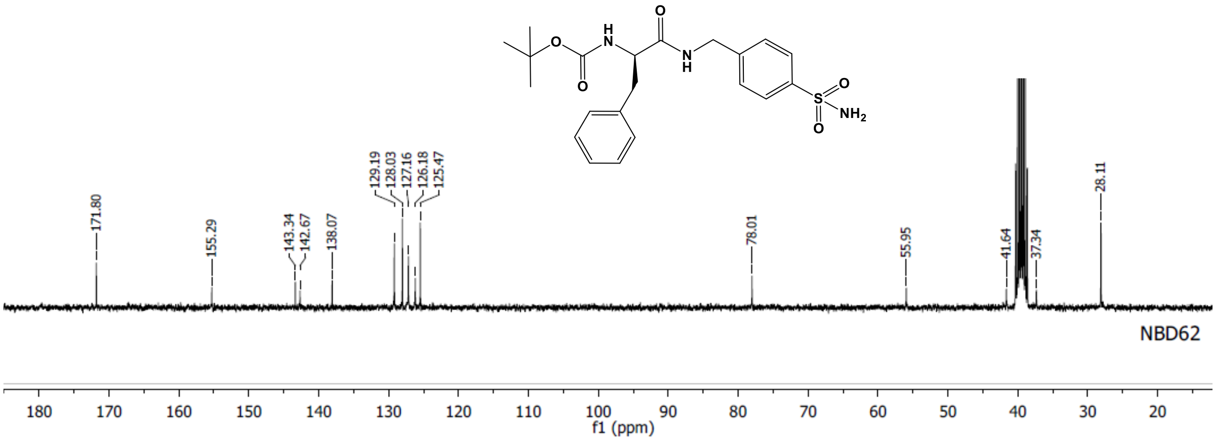

^{13}C spectrum of **19**

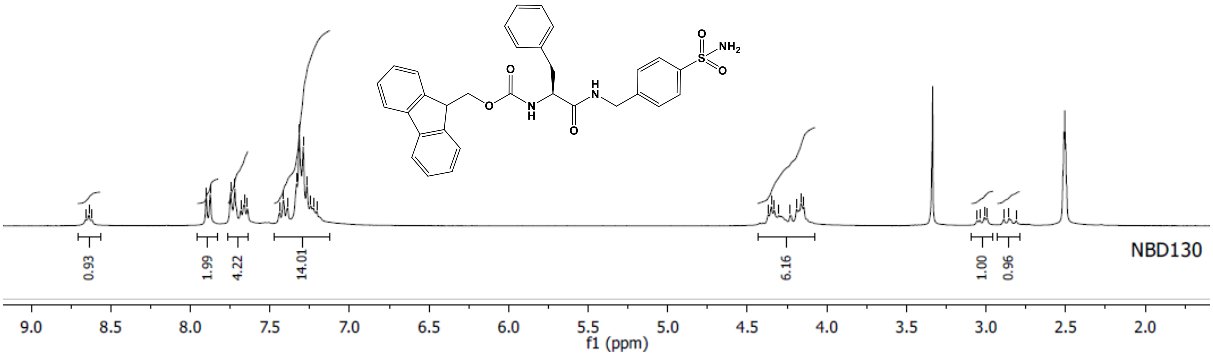

^{1}H spectrum of **20**

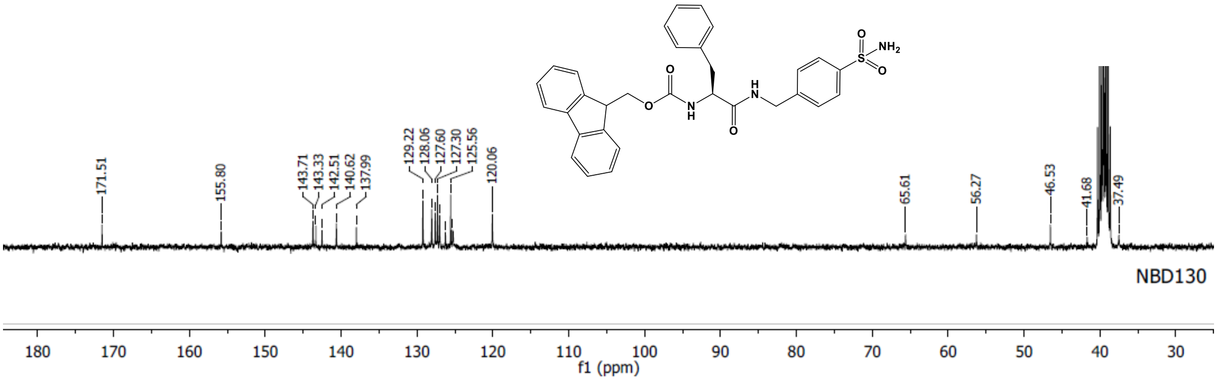

^{13}C spectrum of **20**

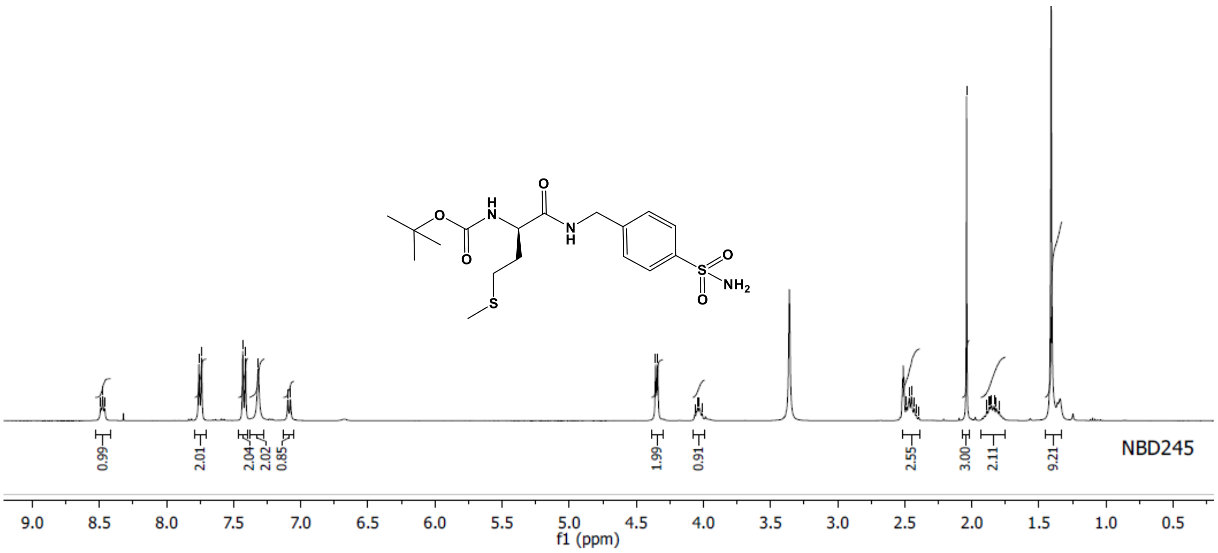

^{1}H spectrum of **21**

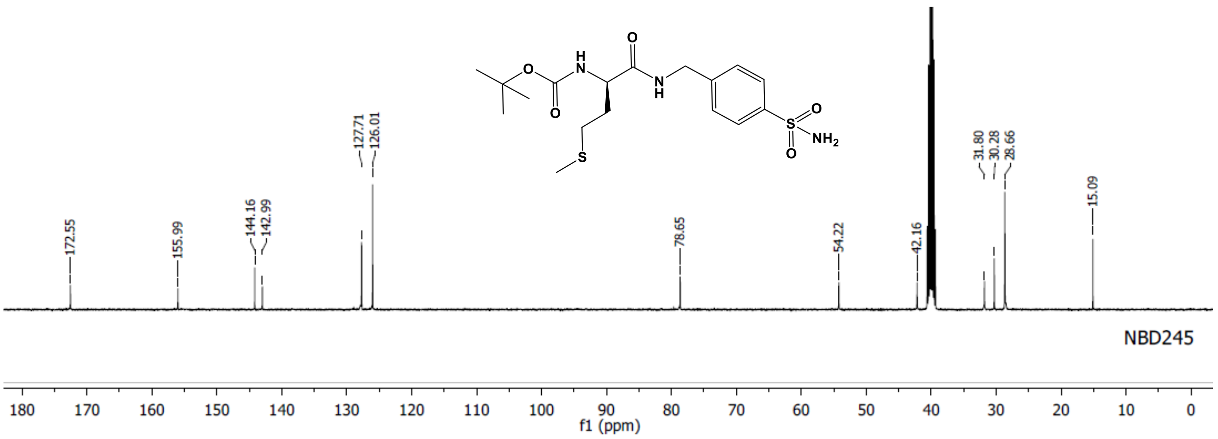

^{13}C spectrum of **21**

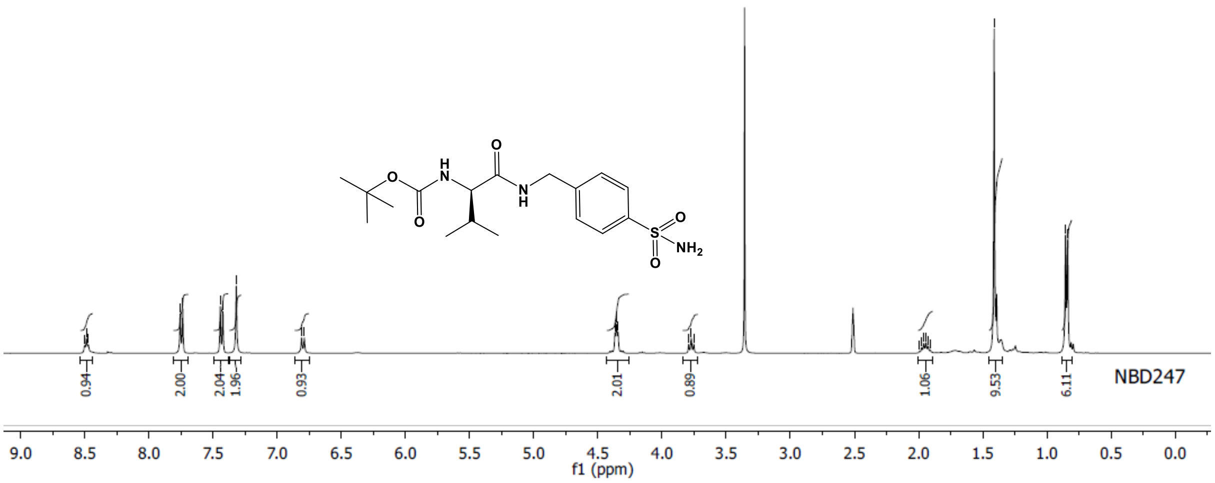

^{1}H spectrum of **22**

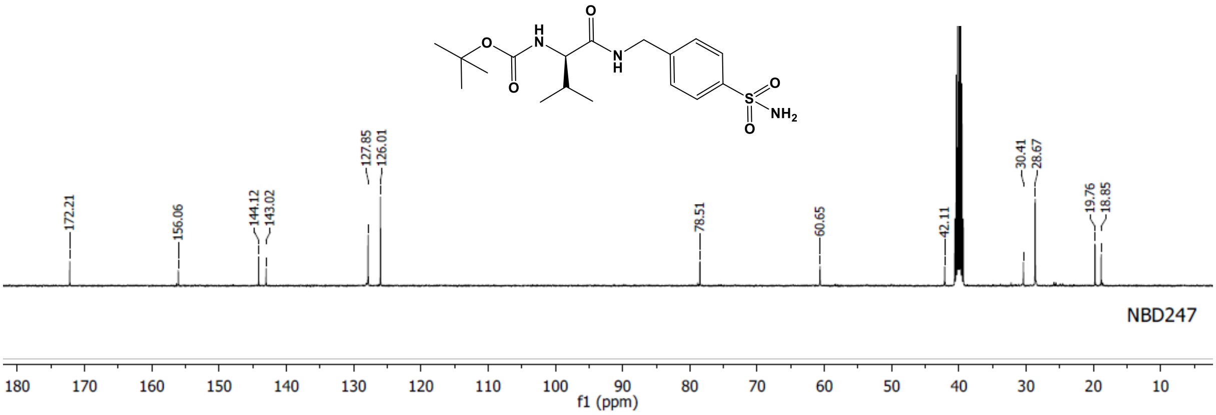

^{13}C spectrum of **22**

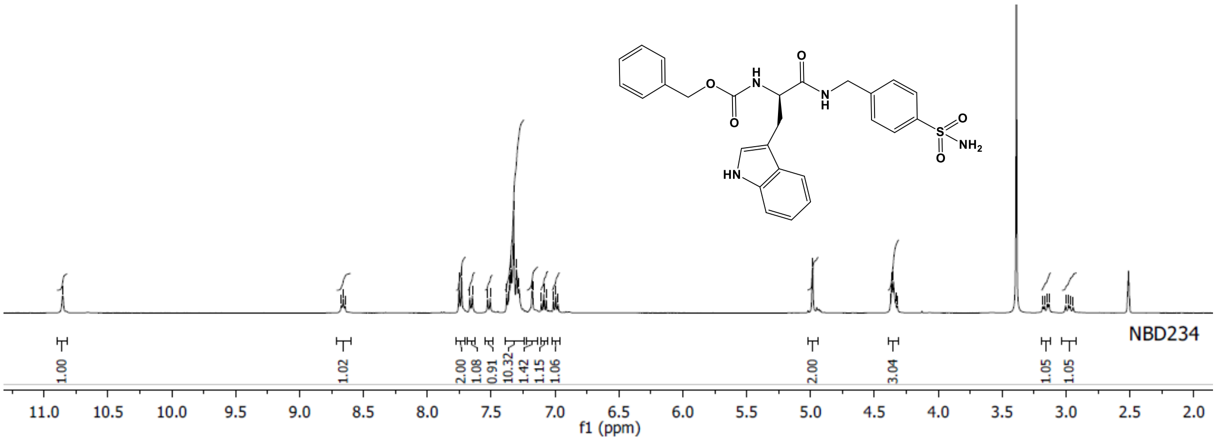

^{1}H spectrum of **23**

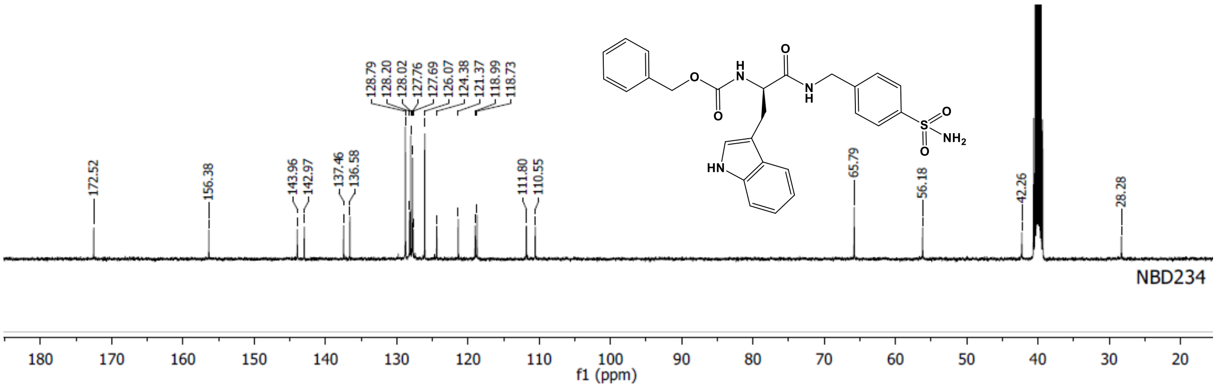

^{13}C spectrum of **23**

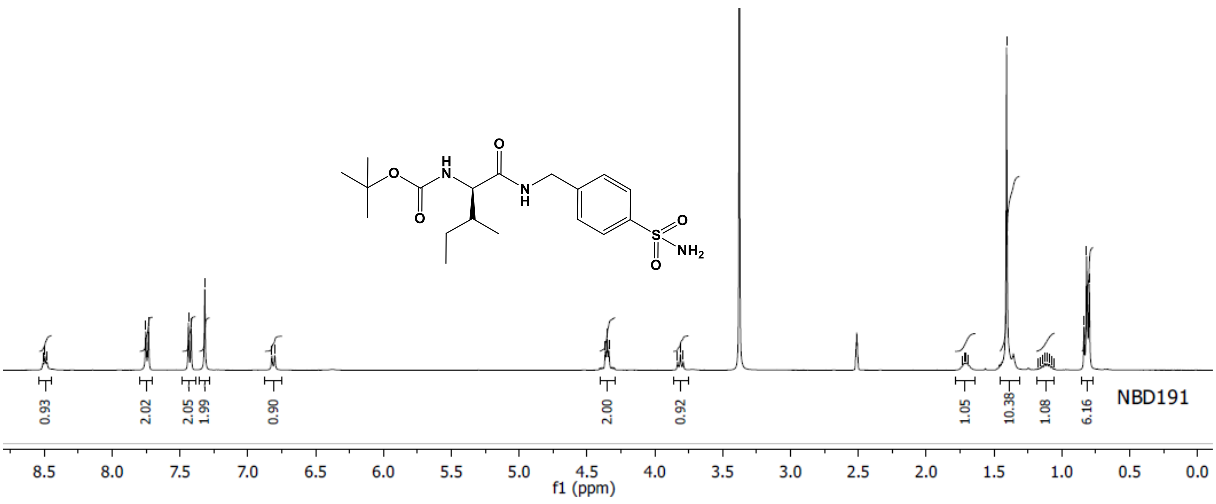

^{1}H spectrum of **24**

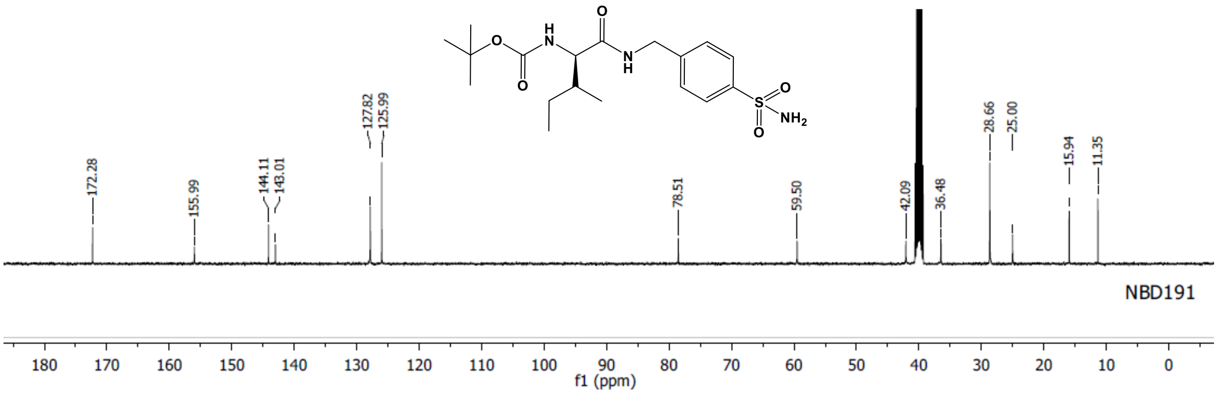

^{13}C spectrum of **24**

CODE	CHEMICAL STRUCTURE	CHEMICAL FORMULA	EXACT MOLECULAR WEIGHT
1	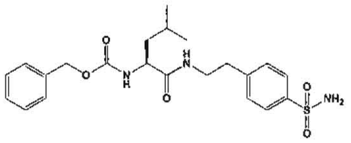	$C_{22}H_{29}N_3O_5S$ 1,42mg	447.18
2	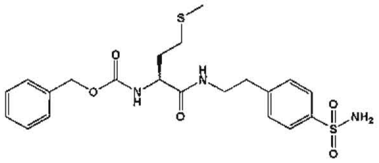	$C_{21}H_{27}N_3O_5S_2$ 1,48mg	465.14
3	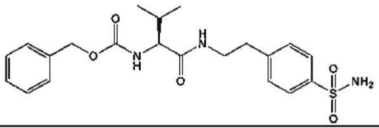	$C_{21}H_{27}N_3O_5S$ 1,02mg	433.17
4	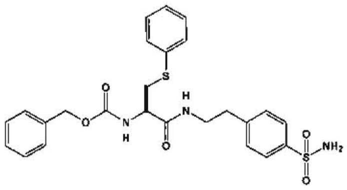	$C_{25}H_{27}N_3O_5S_2$ 1,34mg.	513.14
5	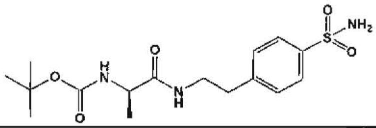	$C_{16}H_{25}N_3O_5S$ 1,49mg	371.15
6	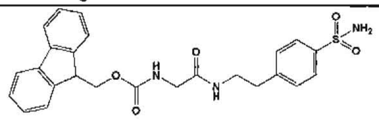	$C_{25}H_{25}N_3O_5S$ 1,24mg	479.15
7	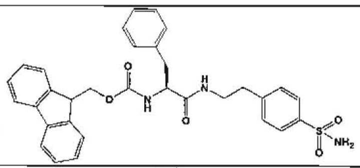	$C_{32}H_{31}N_3O_5S$ 0,98mg	569.20
8	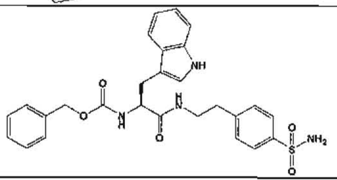	$C_{27}H_{28}N_4O_5S$ 1,36mg	520.18
9	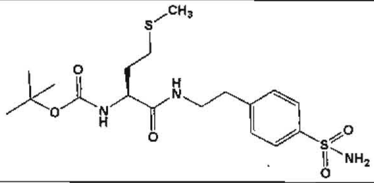	$C_{18}H_{29}N_3O_5S_2$ 1,28mg	431.15

SET C

SET C

**10**	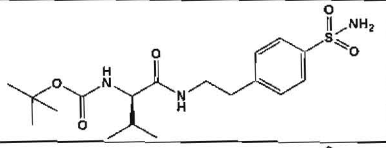	$C_{18}H_{29}N_3O_5S$ 0,89 mg	399.18
**11**	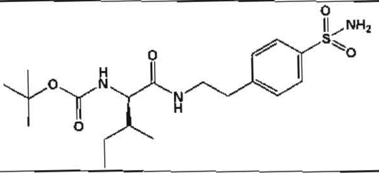	$C_{19}H_{31}N_3O_5S$ 0,92 mg	413.20
**12**	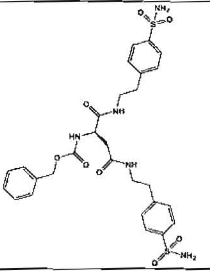	$C_{28}H_{33}N_5O_8S_2$ 1,18 mg.	631.18
**13**	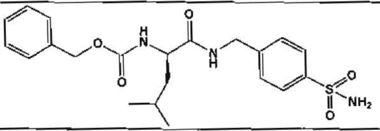	$C_{21}H_{27}N_3O_5S$ 1,02 mg.	433.17
**14**	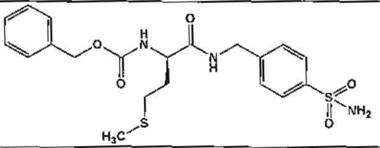	$C_{20}H_{25}N_3O_5S_2$ 1,35 mg.	451.12
**15**	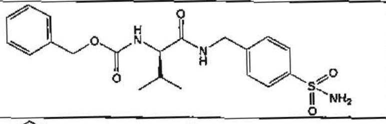	$C_{20}H_{25}N_3O_5S$ 0,80 mg.	419.15
**16**	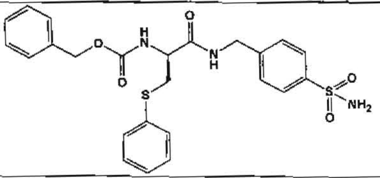	$C_{24}H_{25}N_3O_5S_2$ 1,17 mg.	499.12
**17**	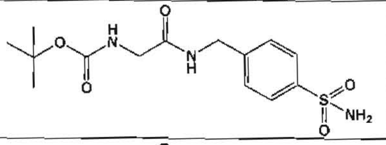	$C_{14}H_{21}N_3O_5S$ 1,47 mg	343.12
**18**	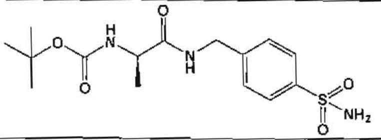	$C_{15}H_{23}N_3O_5S$ 0,82 mg.	357.14
**19**	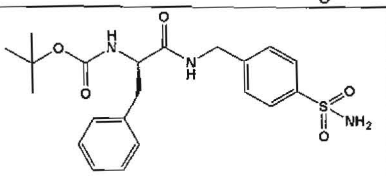	$C_{21}H_{27}N_3O_5S$ 1,22 mg	433.17
**20**	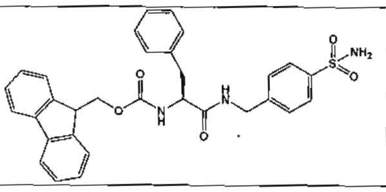	$C_{31}H_{29}N_3O_5S$ 0,90 mg	555.18

**21**	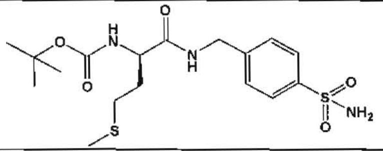	$C_{17}H_{27}N_3O_5S_2$ 1,41 mg.	417.14
**22**	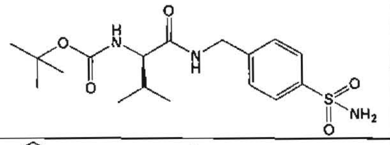	$C_{17}H_{27}N_3O_5S$ 0,93 mg	385.17
**23**	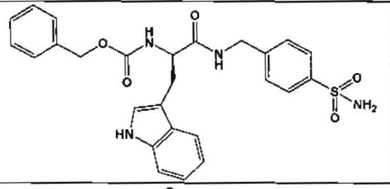	$C_{26}H_{26}N_4O_5S$ 1,10 mg.	506.16
**24**	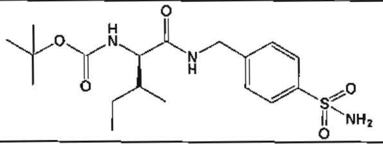	$C_{18}H_{29}N_3O_5S$ 0,85 mg.	399.18

Print Date: 23 Jul 2018 11:20:30

Spectra Plots - 23/07/2018 11:18

1 A Scan 17 from d:\marta\2018\hasan\19 luglio 2018\sample1c_p.xms
1 B Scan 17 from d:\marta\2018\hasan\19 luglio 2018\sample1c_n.xms

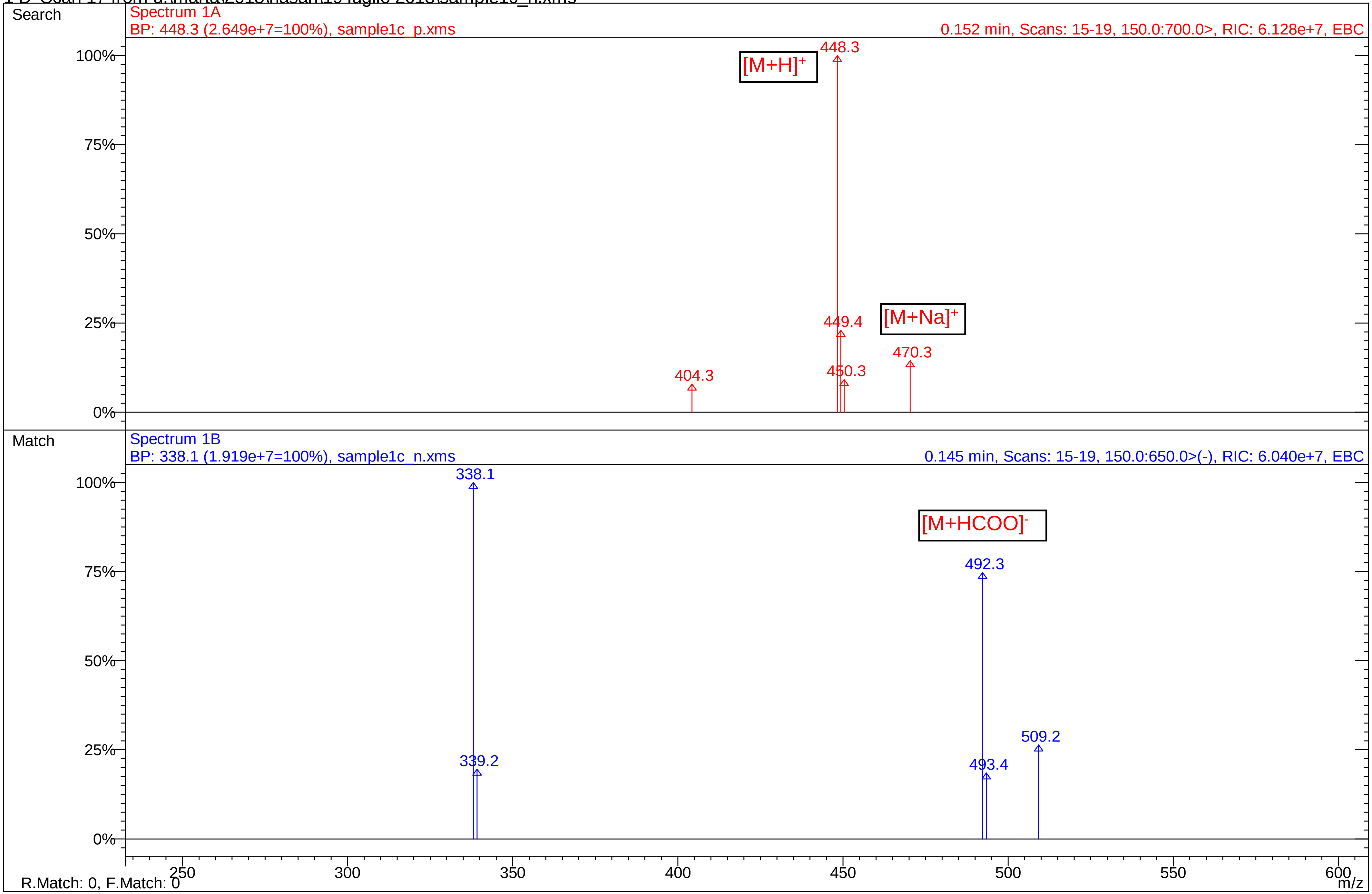

Print Date: 23 Jul 2018 11:24:52

Spectra Plots - 23/07/2018 11:24

1 A Scan 17 from d:\marta\2018\hasan\19 luglio 2018\sample2c_p.xms
1 B Scan 16 from d:\marta\2018\hasan\19 luglio 2018\sample2c_n.xms

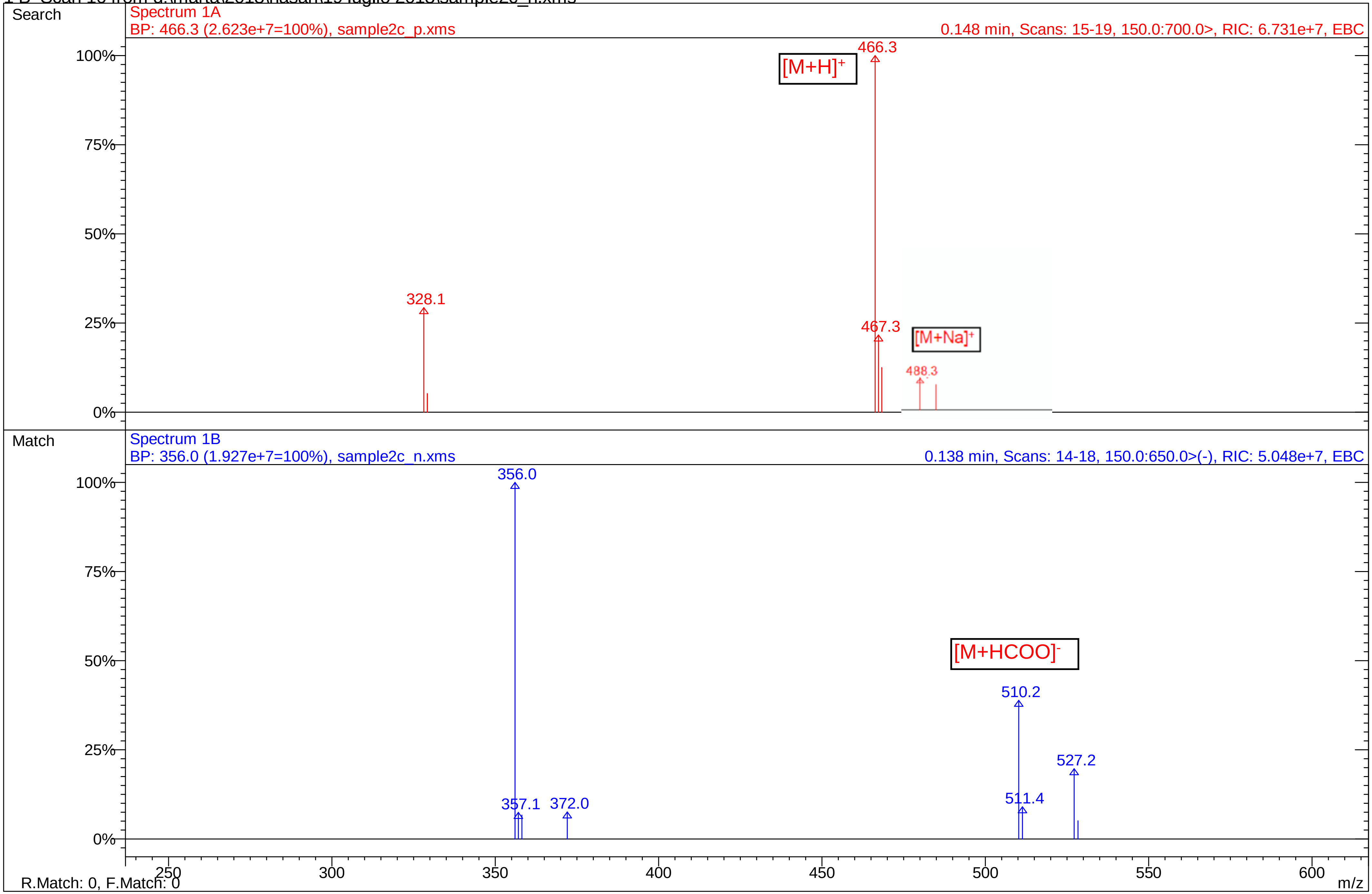

Print Date: 23 Jul 2018 11:26:31

Spectra Plots - 23/07/2018 11:26

1 A Scan 16 from d:\marta\2018\hasan\19 luglio 2018\sample3c_p.xms
1 B Scan 18 from d:\marta\2018\hasan\19 luglio 2018\sample3c_n.xms

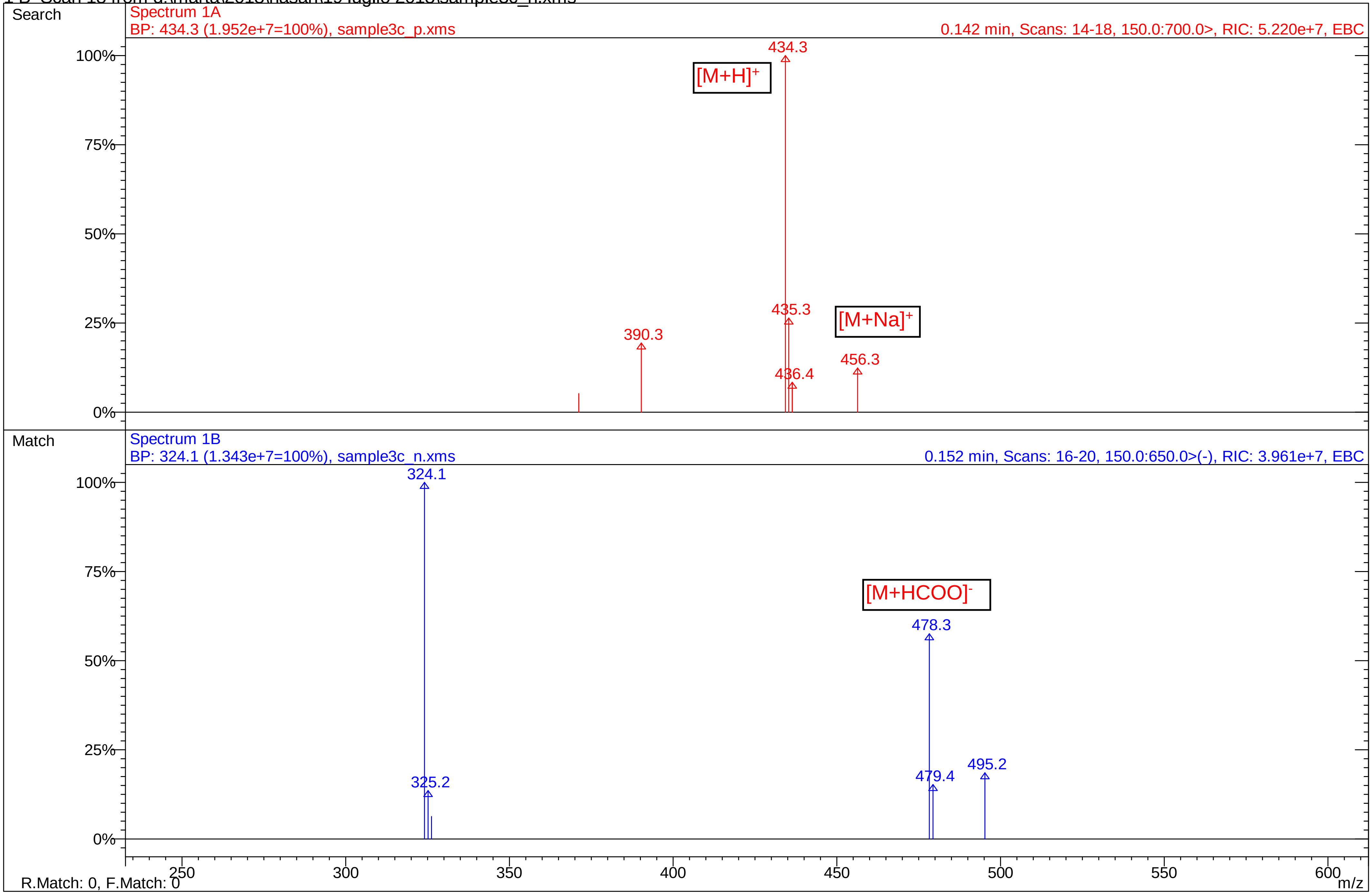

Print Date: 23 Jul 2018 11:27:49

Spectra Plots - 23/07/2018 11:27

1 A Scan 16 from d:\marta\2018\hasan\19 luglio 2018\sample4c_p.xms
1 B Scan 17 from d:\marta\2018\hasan\19 luglio 2018\sample4c_n.xms

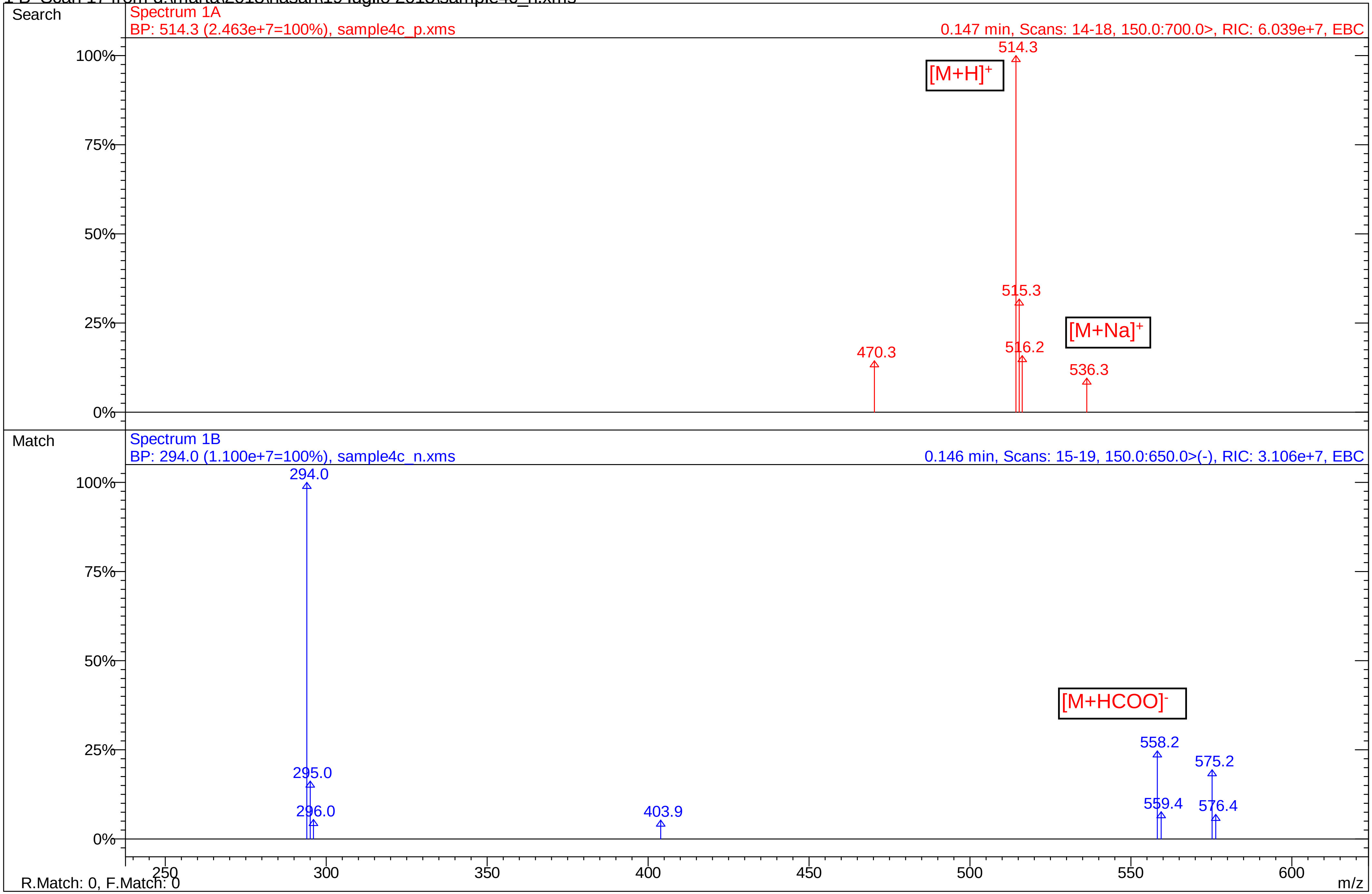

Print Date: 23 Jul 2018 11:29:16

Spectra Plots - 23/07/2018 11:29

1 A Scan 17 from d:\marta\2018\hasan\19 luglio 2018\sample5c_p.xms
1 B Scan 17 from d:\marta\2018\hasan\19 luglio 2018\sample5c_n.xms

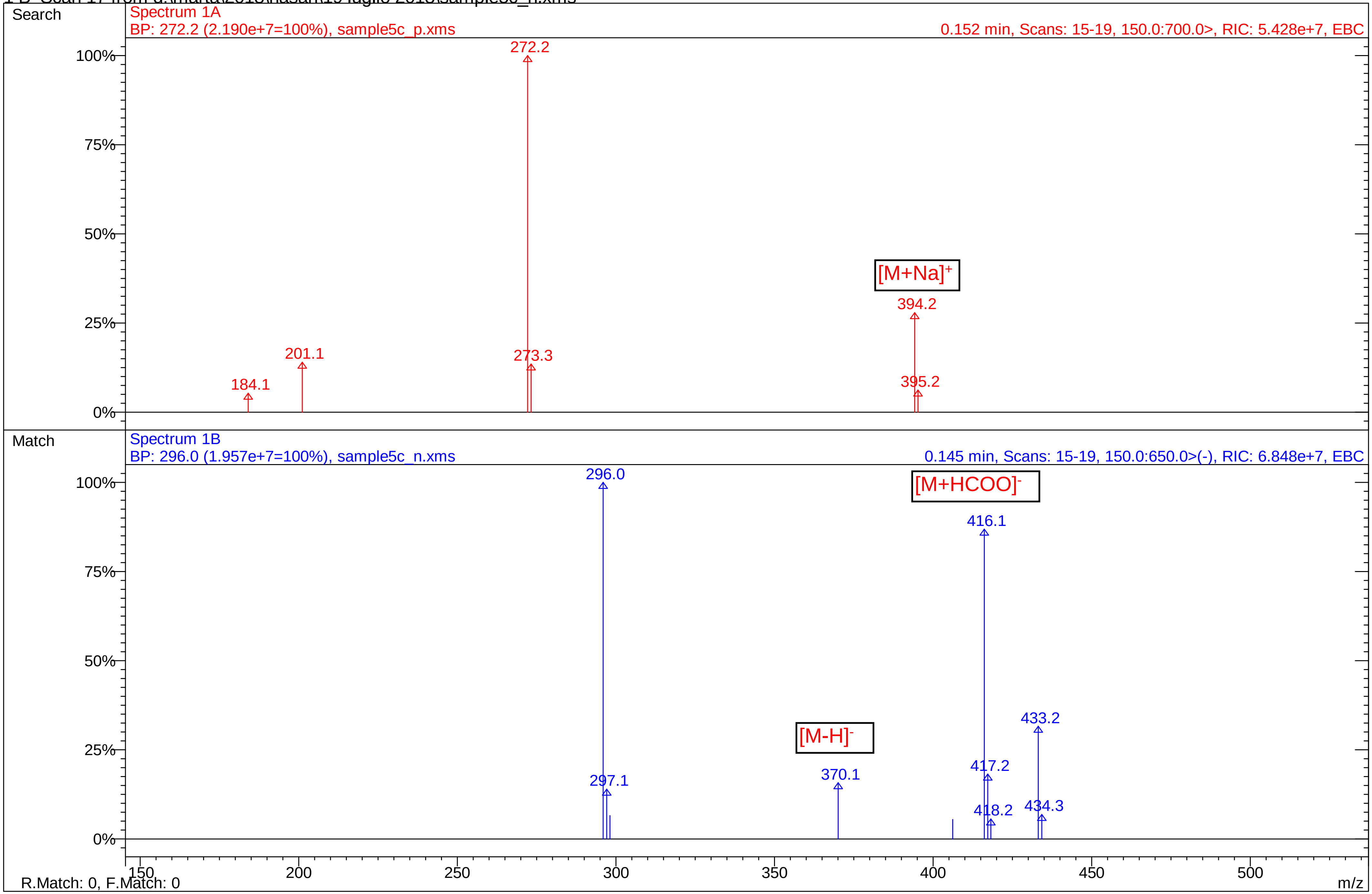

Print Date: 23 Jul 2018 11:30:57

Spectra Plots - 23/07/2018 11:30

1 A Scan 17 from d:\marta\2018\hasan\19 luglio 2018\sample6c_p.xms
1 B Scan 17 from d:\marta\2018\hasan\19 luglio 2018\sample6c_n.xms

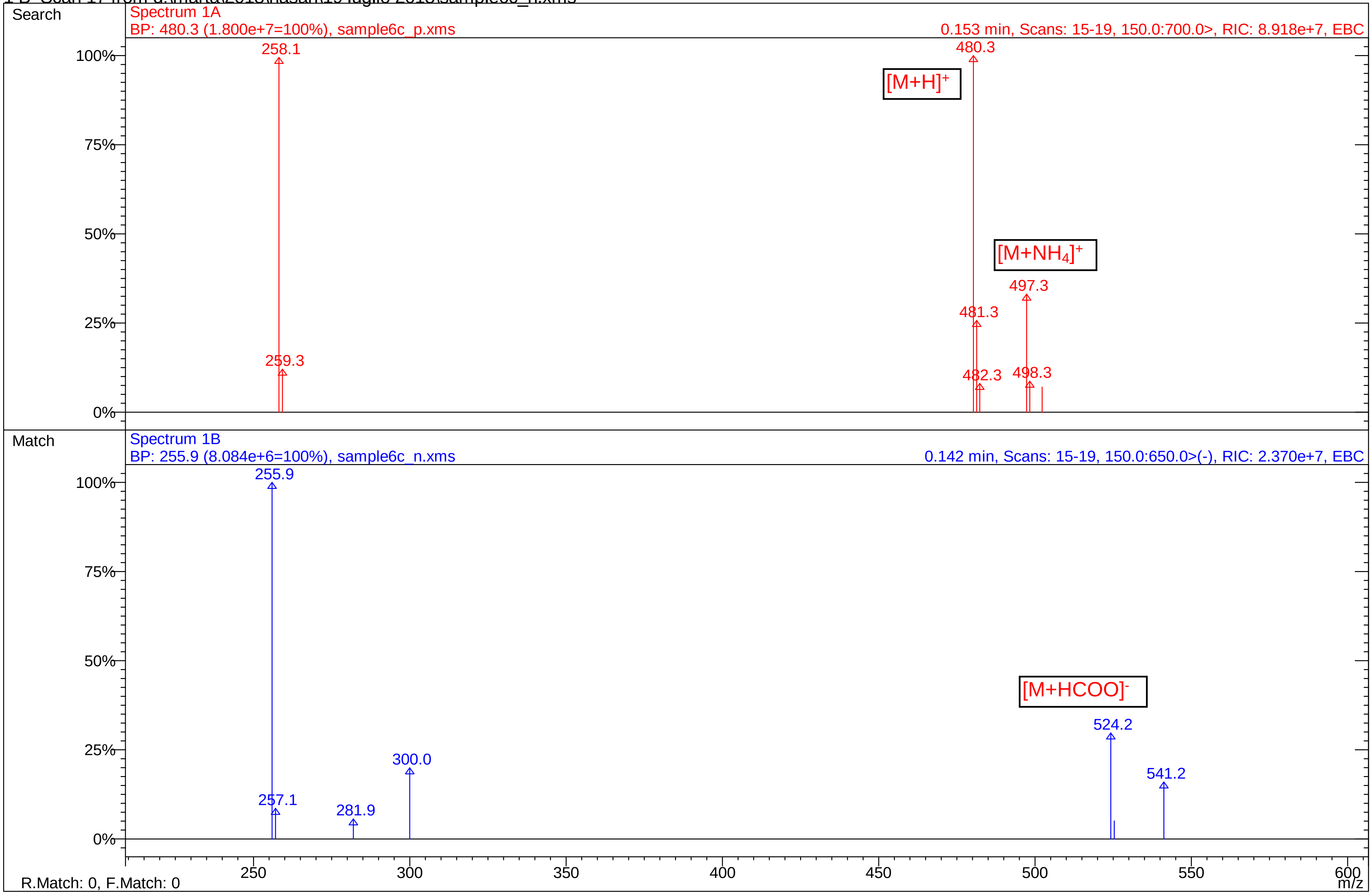

Print Date: 23 Jul 2018 11:33:04

Spectra Plots - 23/07/2018 11:32

1 A Scan 15 from d:\marta\2018\hasan\19 luglio 2018\sample7c_p.xms
1 B Scan 18 from d:\marta\2018\hasan\19 luglio 2018\sample7c_n.xms

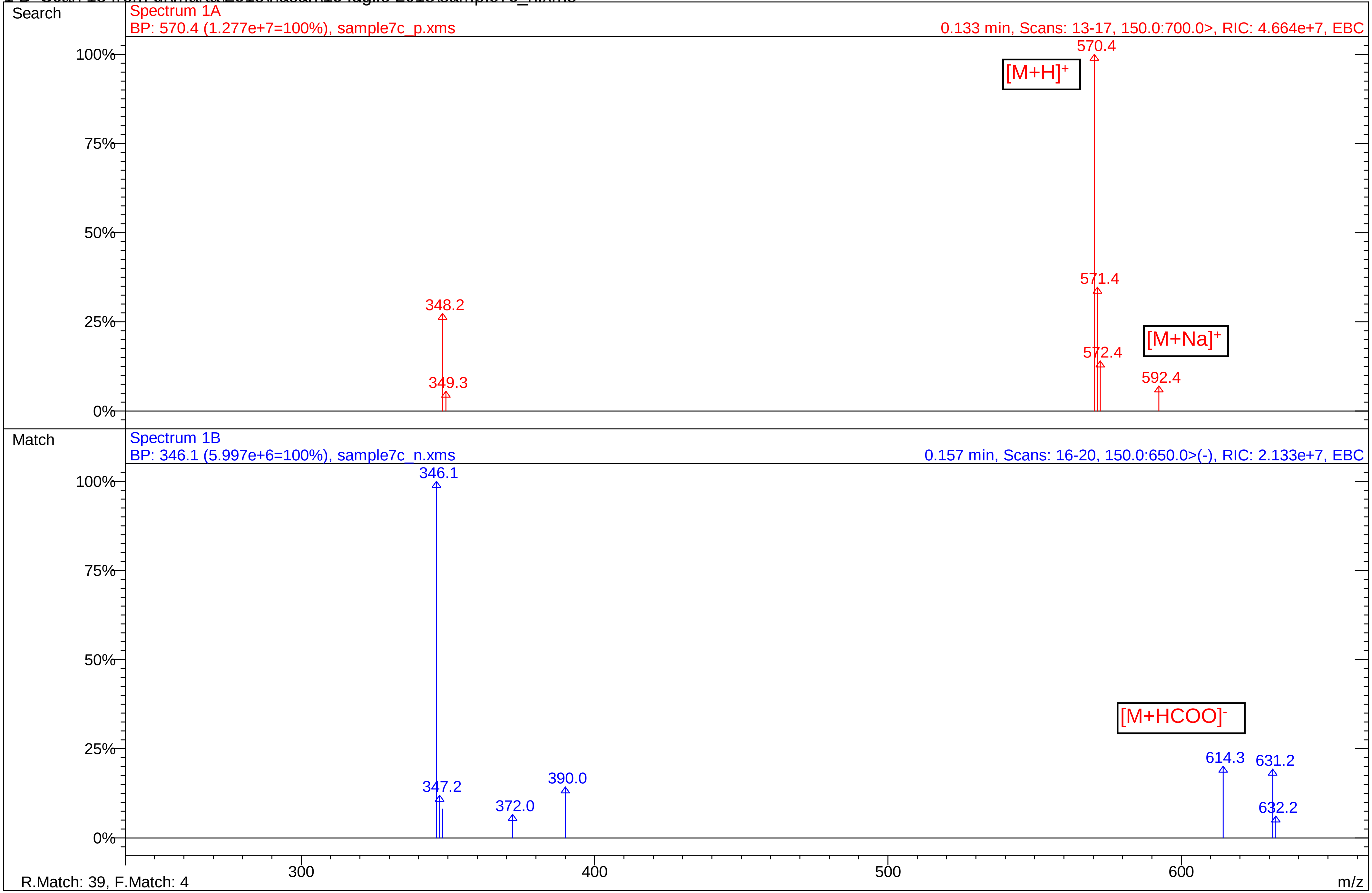

R.Match: 39, F.Match: 4

Print Date: 23 Jul 2018 11:34:19

Spectra Plots - 23/07/2018 11:34

1 A Scan 16 from d:\marta\2018\hasan\19 luglio 2018\sample8c_p.xms
1 B Scan 16 from d:\marta\2018\hasan\19 luglio 2018\sample8c_n.xms

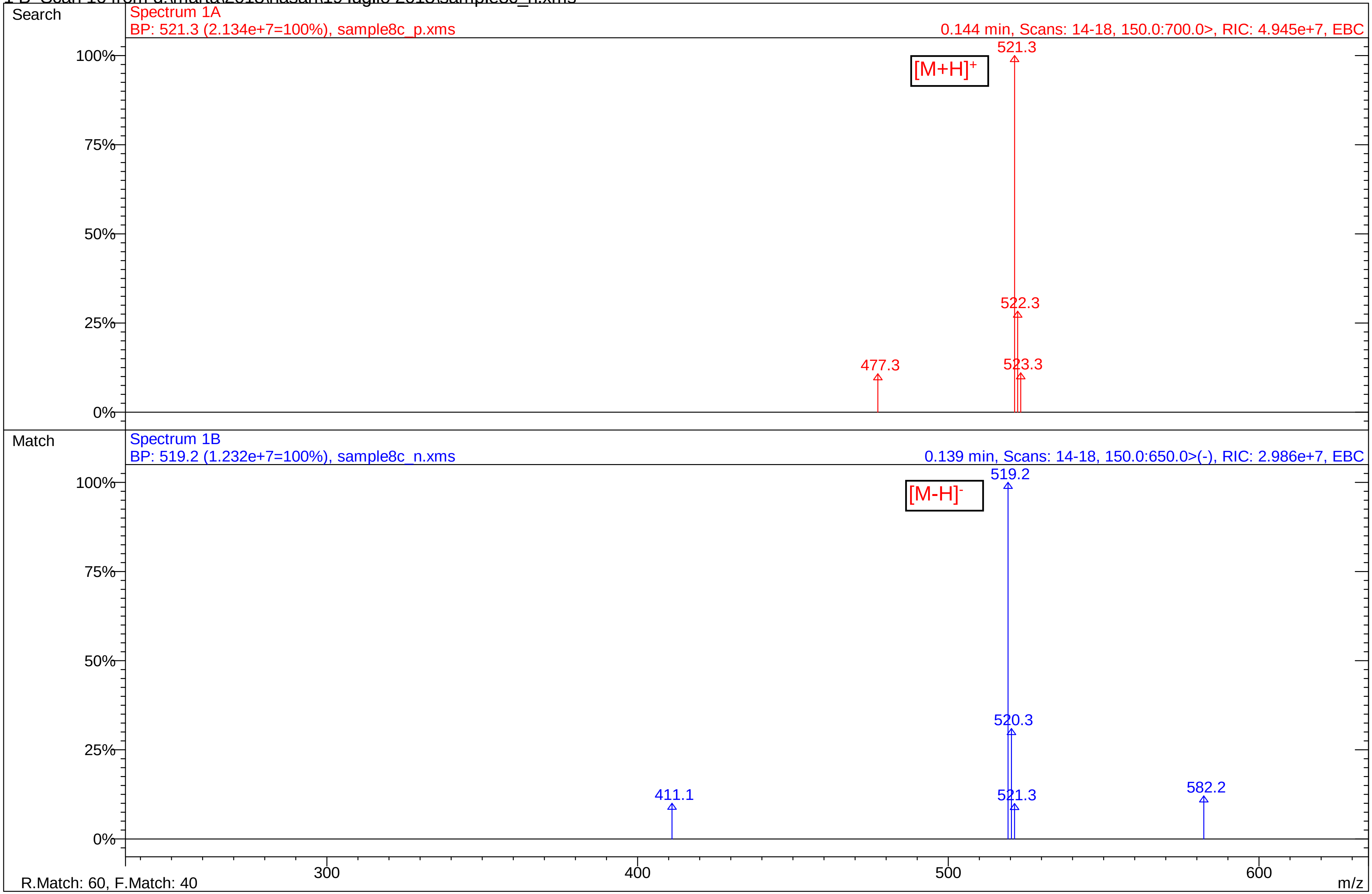

Print Date: 23 Jul 2018 11:35:37

Spectra Plots - 23/07/2018 11:35

1 A Scan 17 from d:\marta\2018\hasan\19 luglio 2018\sample9c_p.xms
1 B Scan 14 from d:\marta\2018\hasan\19 luglio 2018\sample9c_n.xms

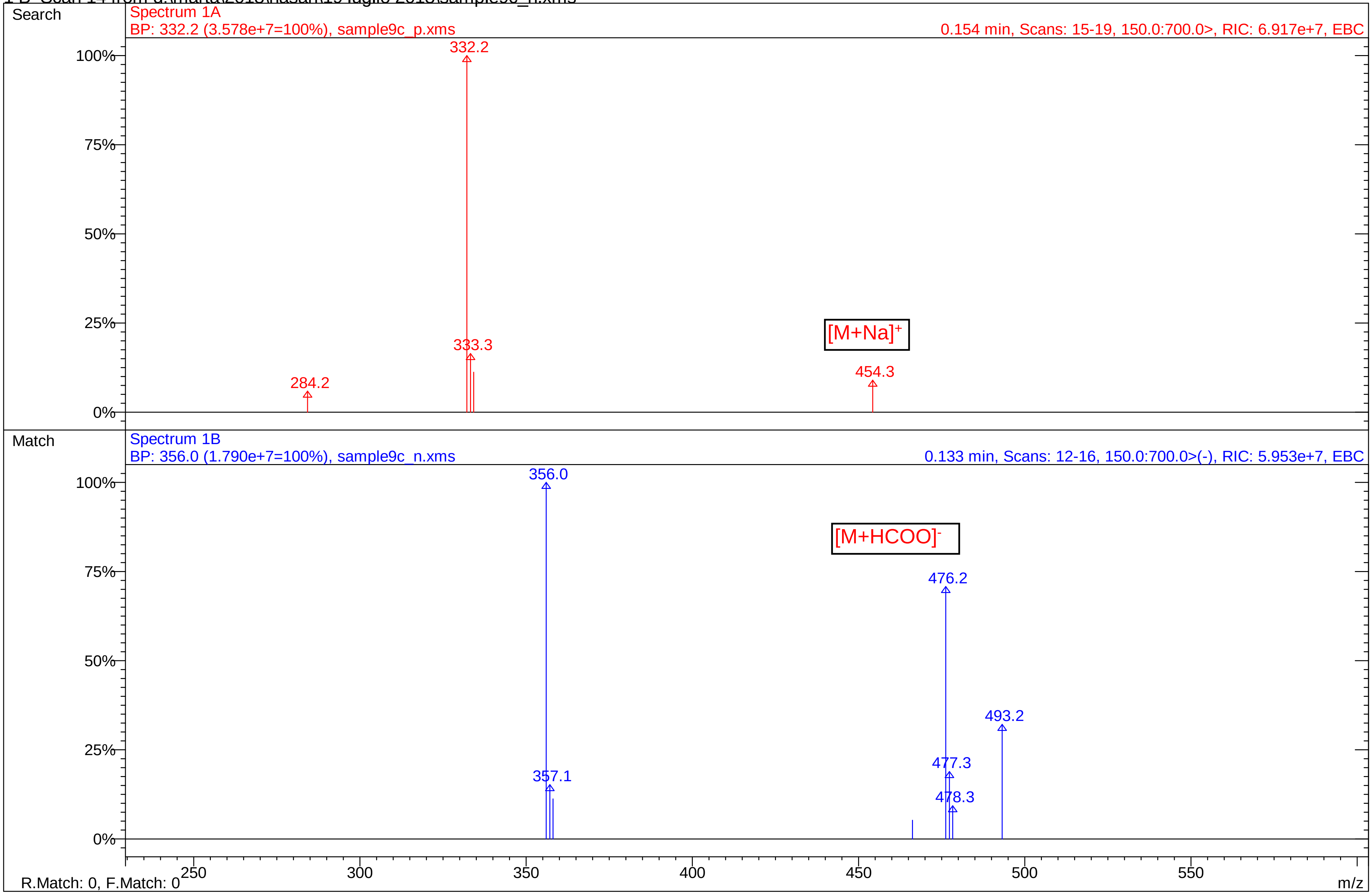

Print Date: 23 Jul 2018 11:37:07

Spectra Plots - 23/07/2018 11:37

1 A Scan 17 from d:\marta\2018\hasan\19 luglio 2018\sample10c_p.xms
1 B Scan 14 from d:\marta\2018\hasan\19 luglio 2018\sample10c_n.xms

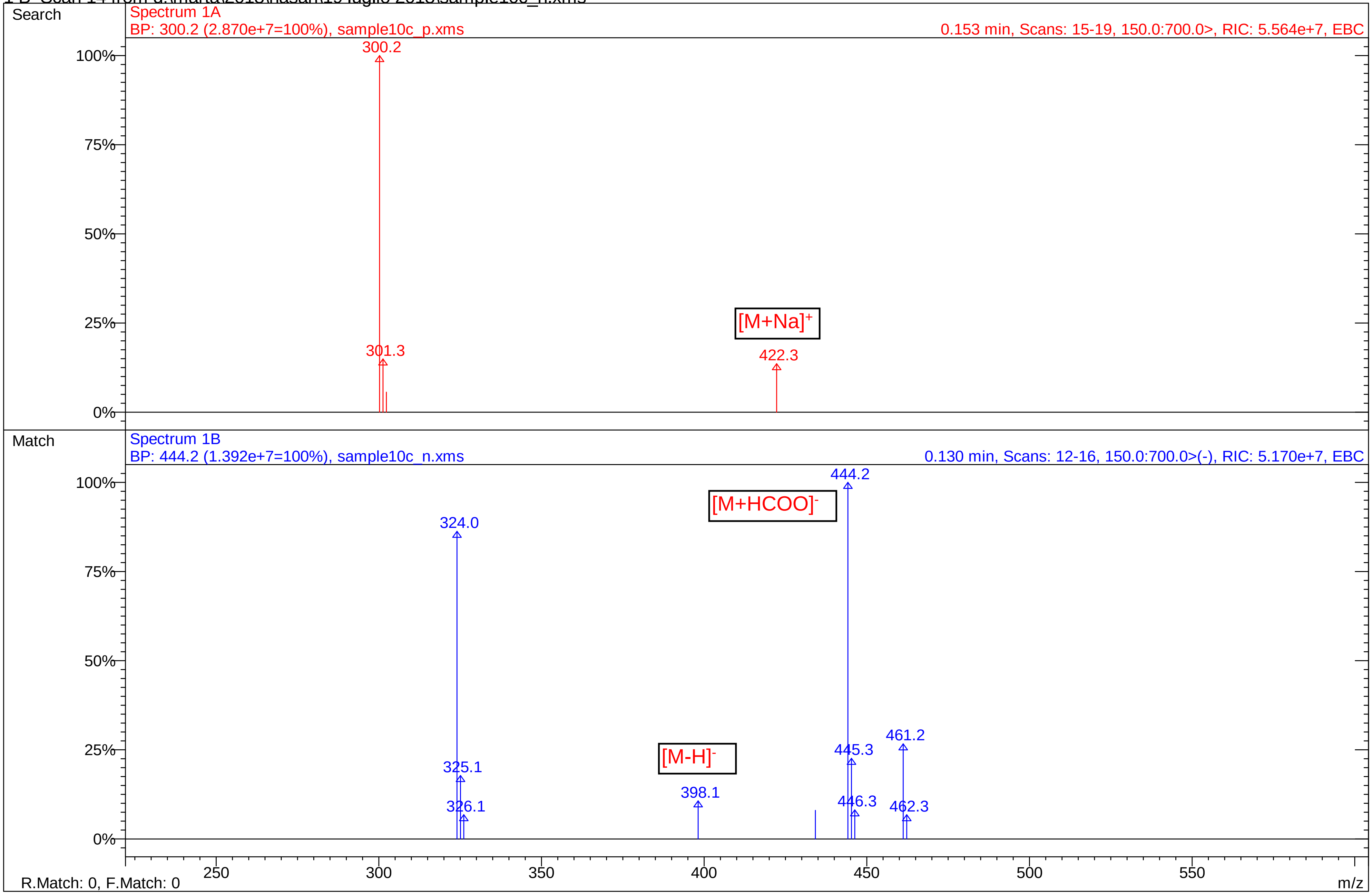

Print Date: 23 Jul 2018 11:38:33

Spectra Plots - 23/07/2018 11:38

1 A Scan 17 from d:\marta\2018\hasan\19 luglio 2018\sample11c_p.xms
1 B Scan 16 from d:\marta\2018\hasan\19 luglio 2018\sample11c_n.xms

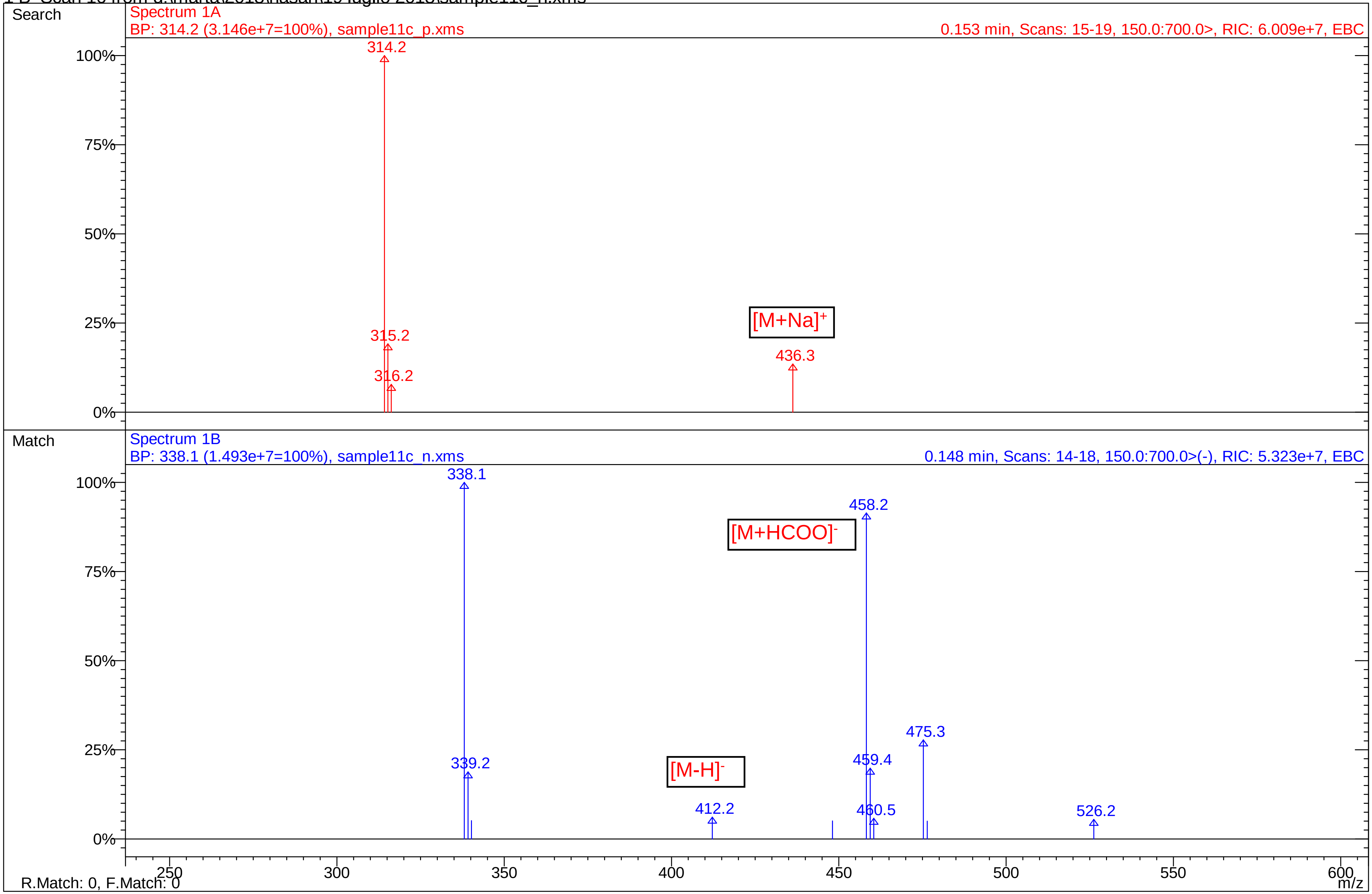

Print Date: 23 Jul 2018 11:40:10

Spectra Plots - 23/07/2018 11:40

1 A Scan 17 from d:\marta\2018\hasan\19 luglio 2018\sample12c_p.xms
1 B Scan 16 from d:\marta\2018\hasan\19 luglio 2018\sample12c_n.xms

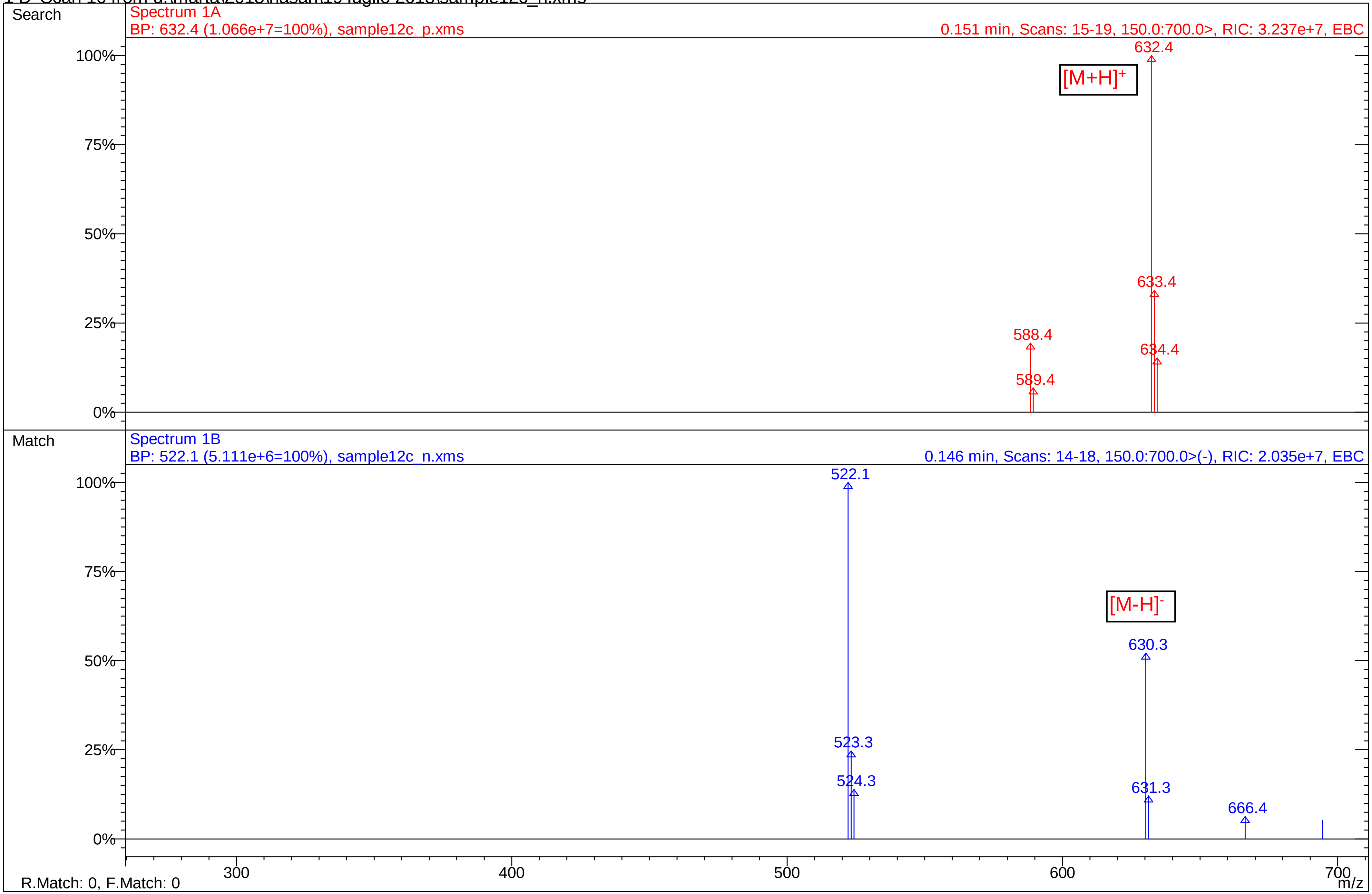

Print Date: 23 Jul 2018 11:42:08

Spectra Plots - 23/07/2018 11:42

1 A Scan 17 from d:\marta\2018\hasan\19 luglio 2018\sample13c_p.xms
1 B Scan 16 from d:\marta\2018\hasan\19 luglio 2018\sample13c_n.xms

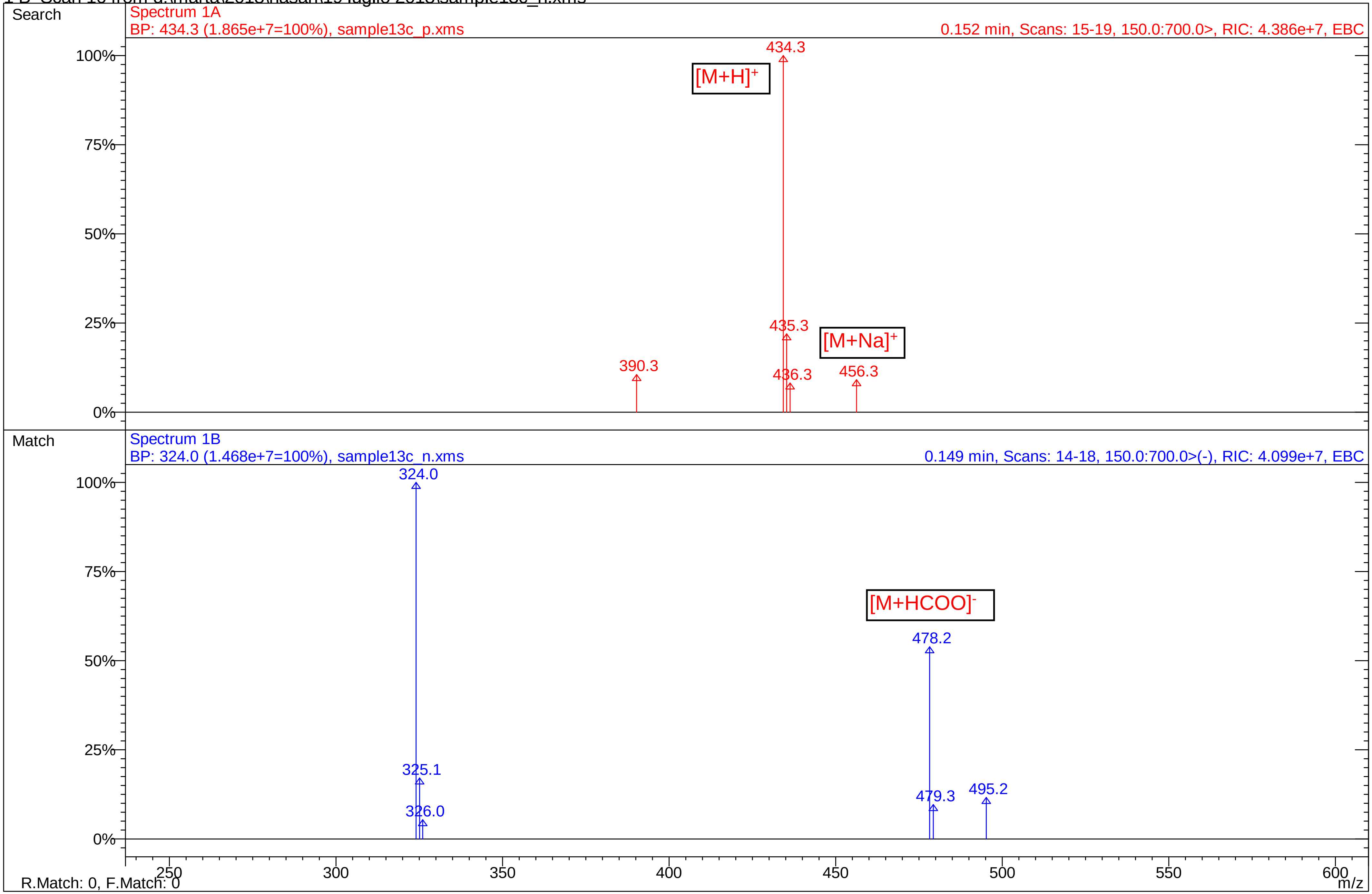

Print Date: 23 Jul 2018 11:49:54

Spectra Plots - 23/07/2018 11:43

1 A Scan 18 from d:\marta\2018\hasan\19 luglio 2018\sample14c_p.xms
1 B Scan 15 from d:\marta\2018\hasan\19 luglio 2018\sample14c_n.xms

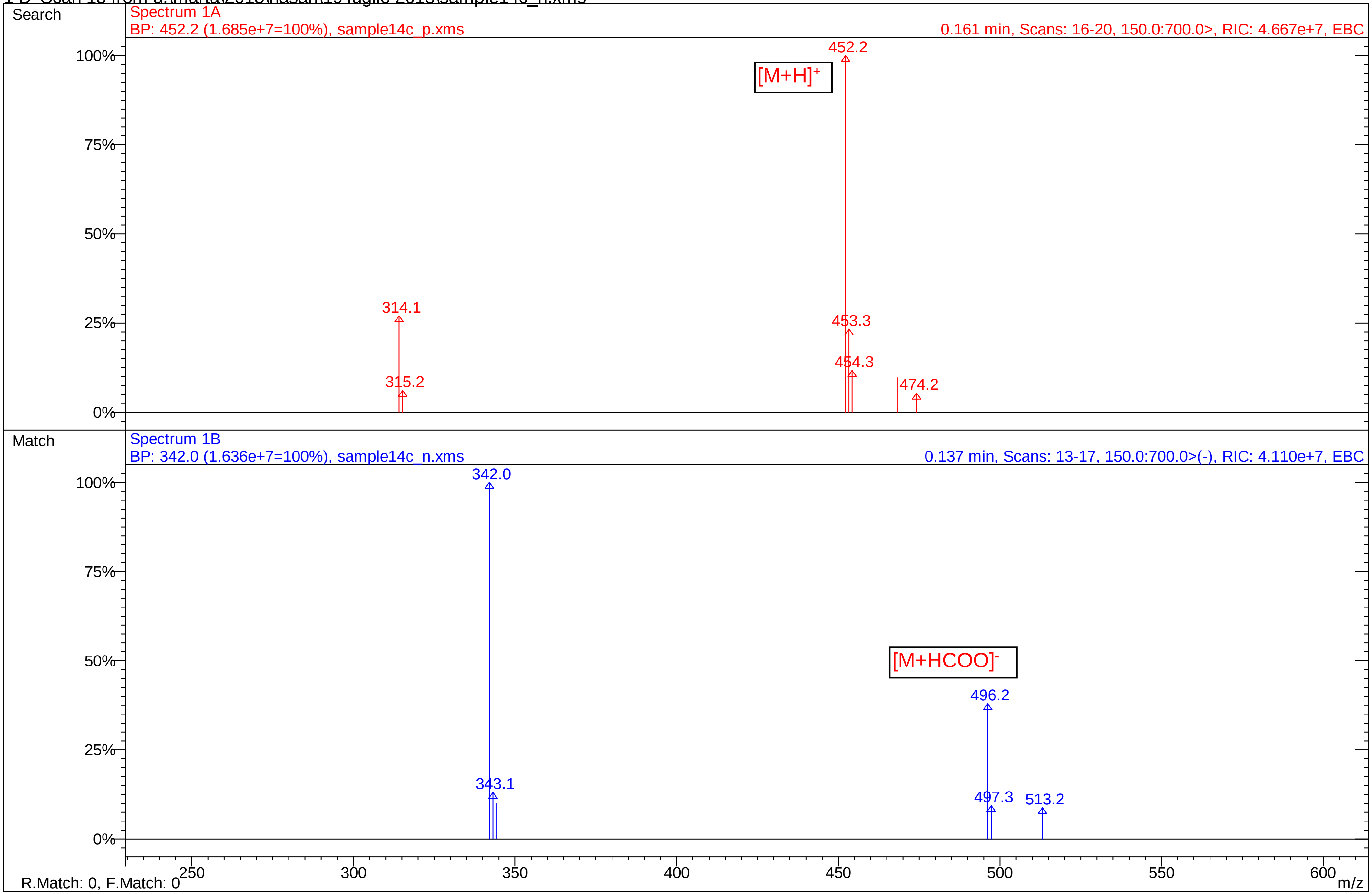

Print Date: 23 Jul 2018 11:51:03

Spectra Plots - 23/07/2018 11:50

1 A Scan 17 from d:\marta\2018\hasan\19 luglio 2018\sample15c_p.xms
1 B Scan 17 from d:\marta\2018\hasan\19 luglio 2018\sample15c_n.xms

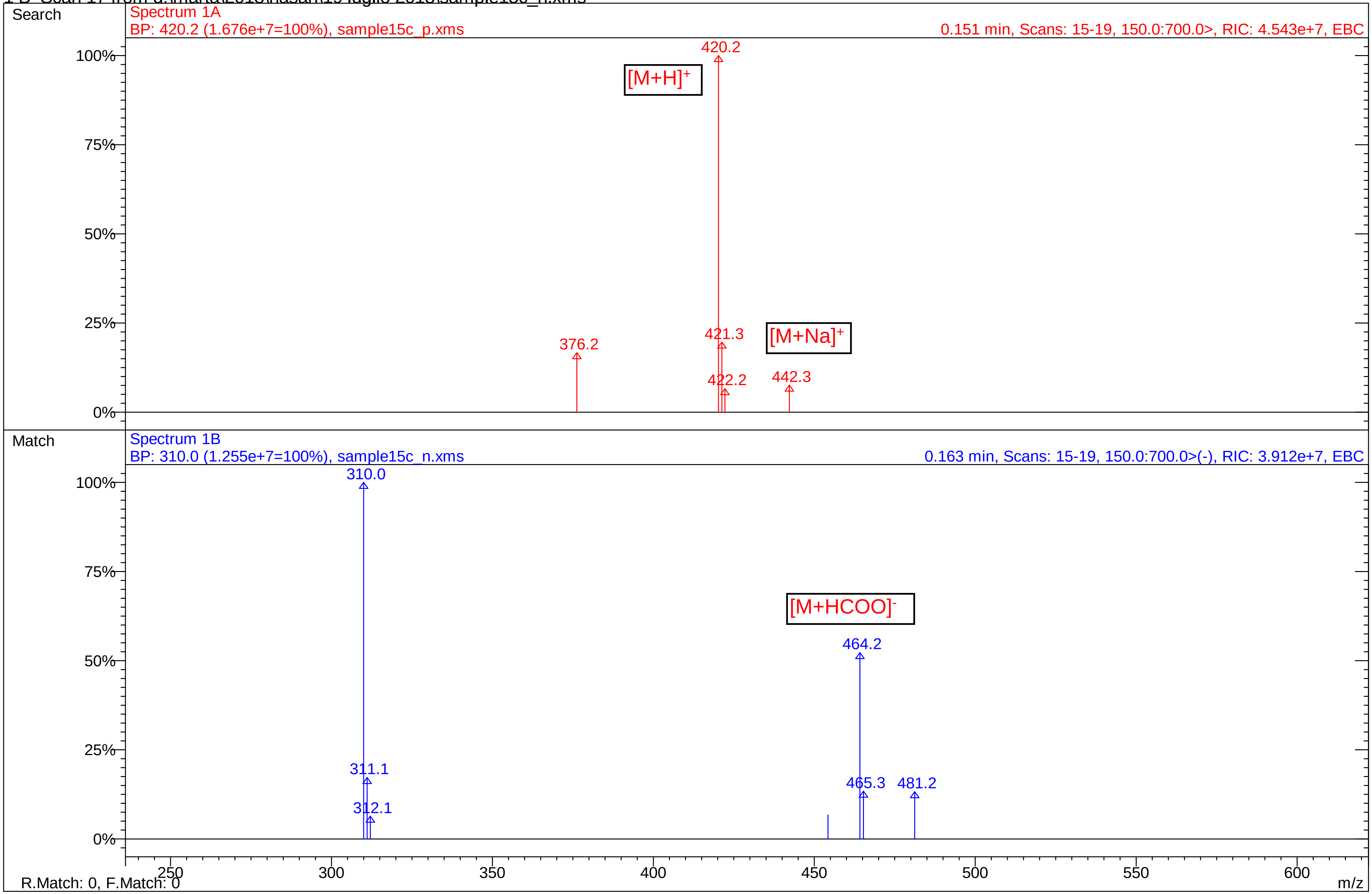

Spectra Plots - 23/07/2018 11:52

1 A Scan 18 from d:\marta\2018\hasan\19 luglio 2018\sample16c_p.xms
1 B Scan 16 from d:\marta\2018\hasan\19 luglio 2018\sample16c_n.xms

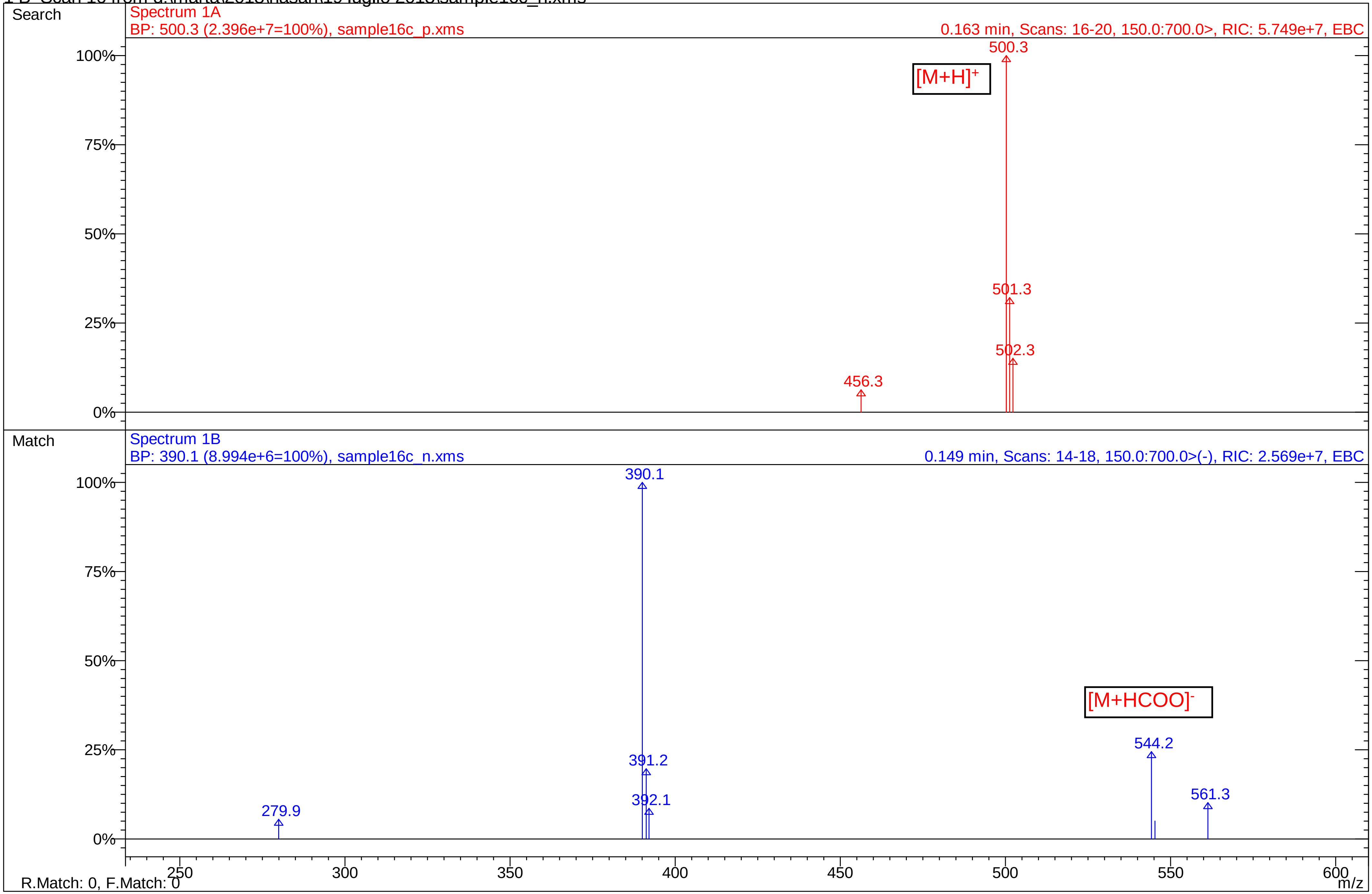

Print Date: 23 Jul 2018 11:53:21

Spectra Plots - 23/07/2018 11:53

1 A Scan 18 from d:\marta\2018\hasan\19 luglio 2018\sample17c_p.xms
1 B Scan 16 from d:\marta\2018\hasan\19 luglio 2018\sample17c_n.xms

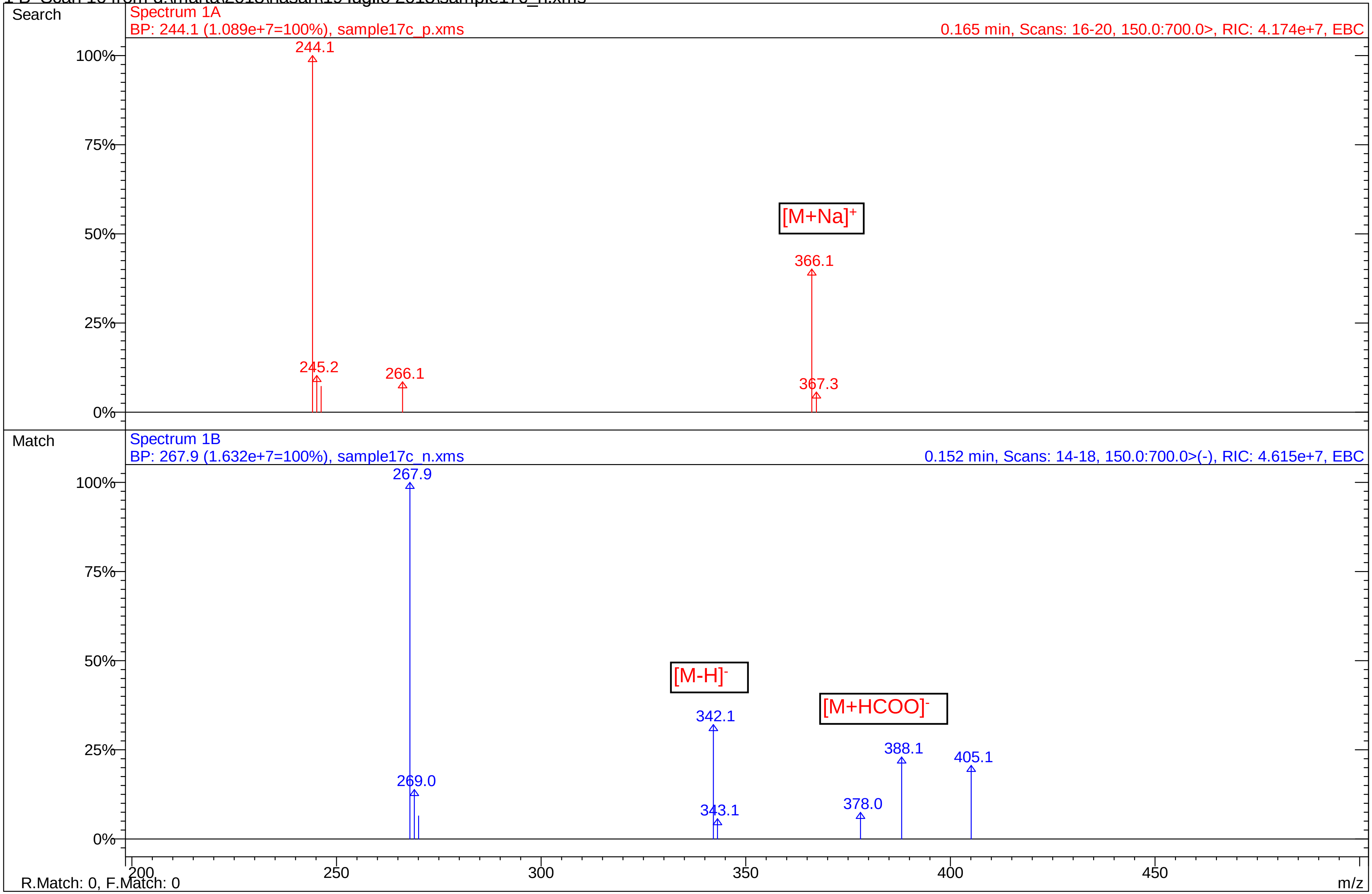

Print Date: 23 Jul 2018 12:07:22

Spectra Plots - 23/07/2018 12:07

1 A Scan 18 from d:\marta\2018\hasan\19 luglio 2018\sample18c_p.xms
1 B Scan 15 from d:\marta\2018\hasan\19 luglio 2018\sample18c_n.xms

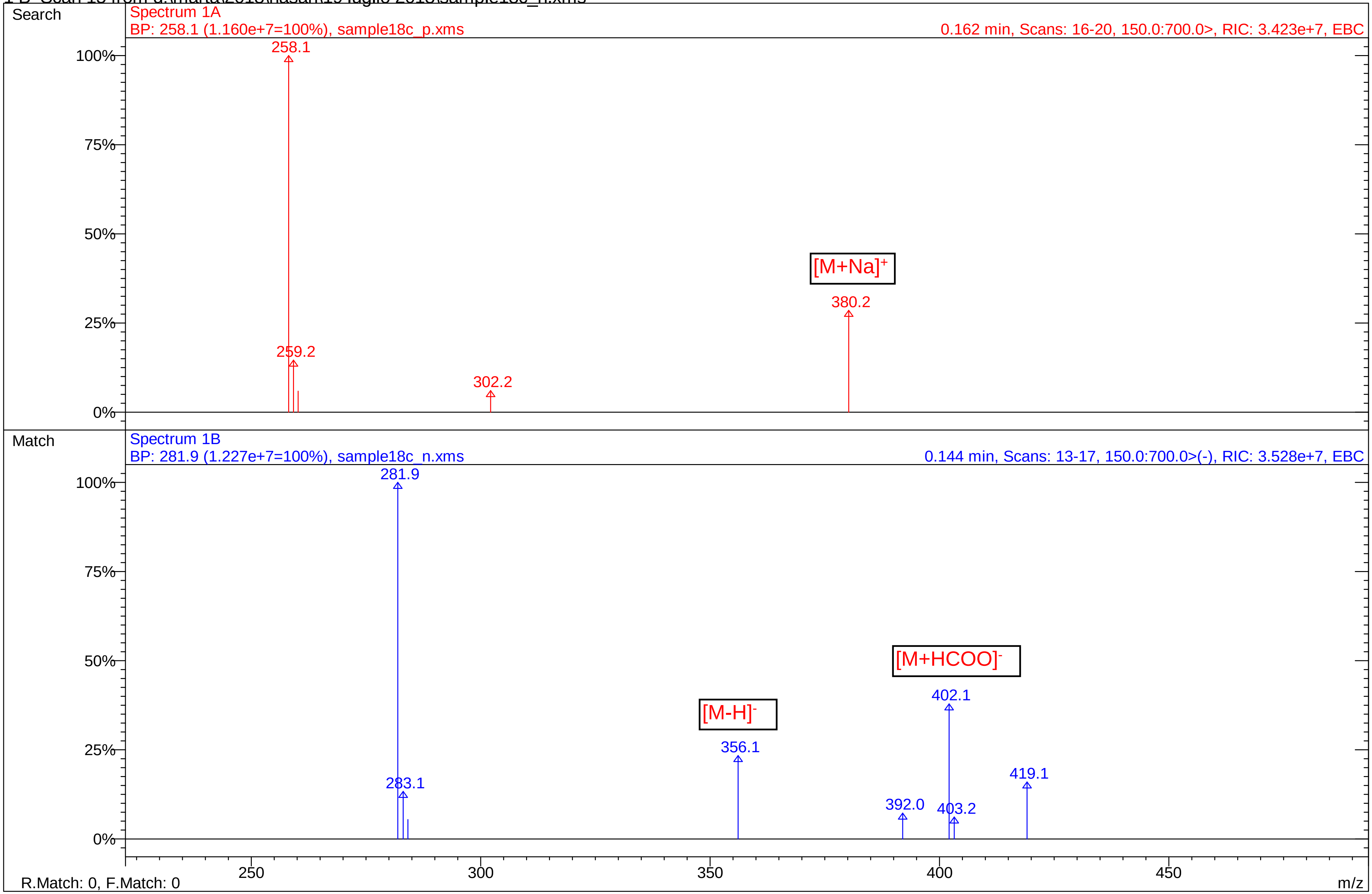

Print Date: 23 Jul 2018 12:08:35

Spectra Plots - 23/07/2018 12:08

1 A Scan 17 from d:\marta\2018\hasan\19 luglio 2018\sample19c_p.xms
1 B Scan 15 from d:\marta\2018\hasan\19 luglio 2018\sample19c_n.xms

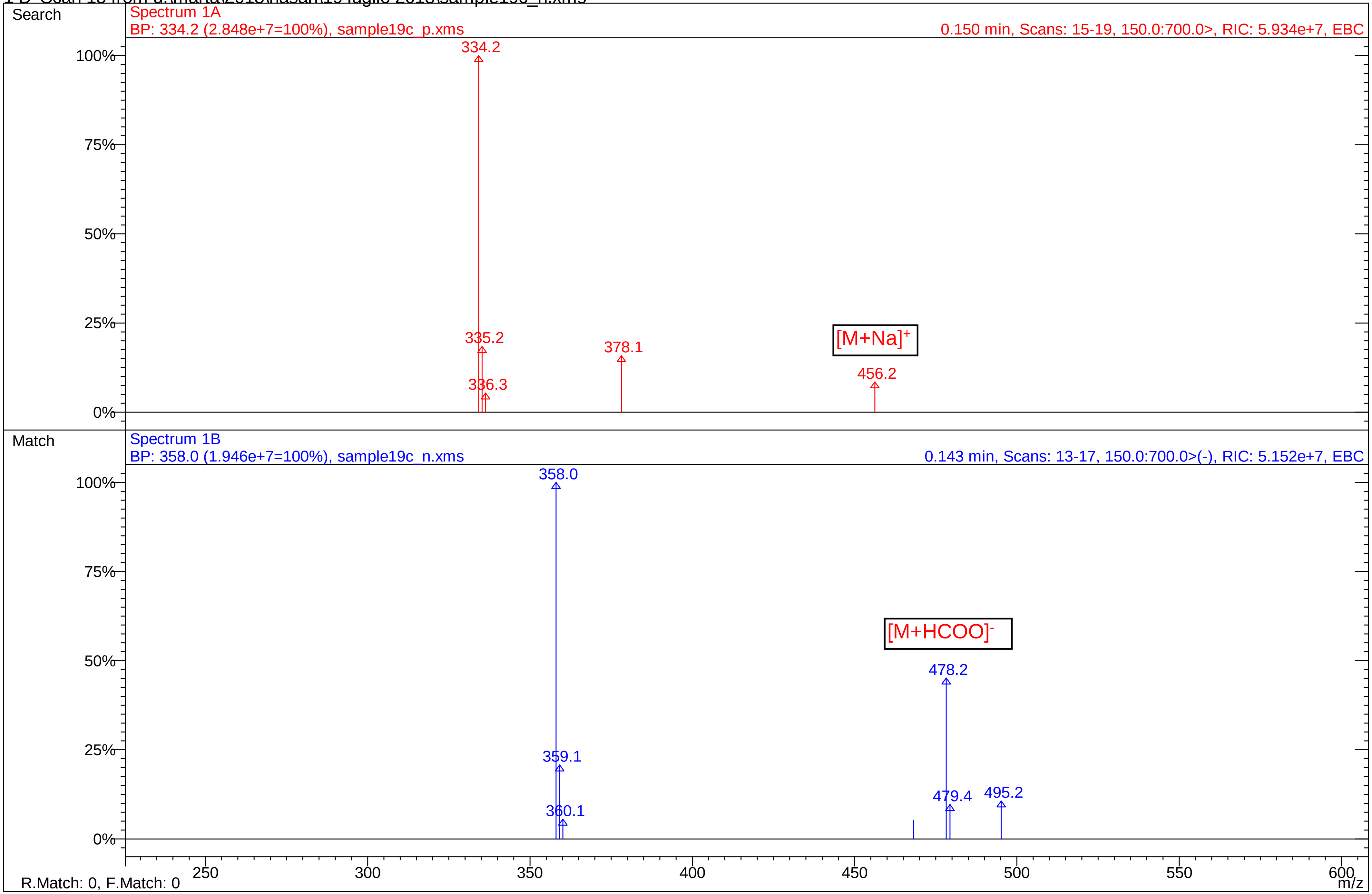

Print Date: 23 Jul 2018 12:09:36

Spectra Plots - 23/07/2018 12:09

1 A Scan 17 from d:\marta\2018\hasan\19 luglio 2018\sample20c_p.xms
1 B Scan 15 from d:\marta\2018\hasan\19 luglio 2018\sample20c_n.xms

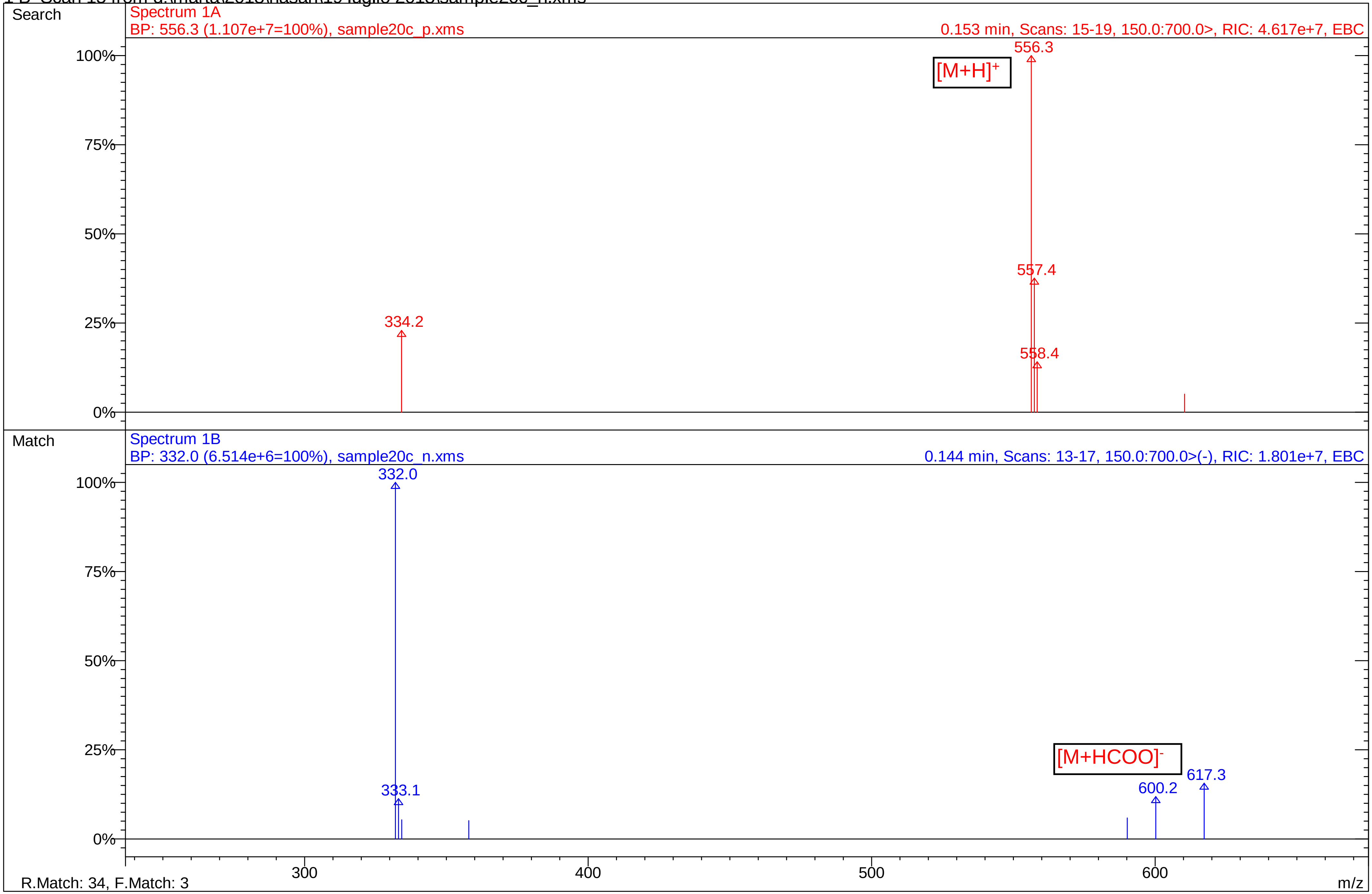

Print Date: 23 Jul 2018 12:10:54

Spectra Plots - 23/07/2018 12:10

1 A Scan 17 from d:\marta\2018\hasan\19 luglio 2018\sample21c_p.xms
1 B Scan 16 from d:\marta\2018\hasan\19 luglio 2018\sample21c_n.xms

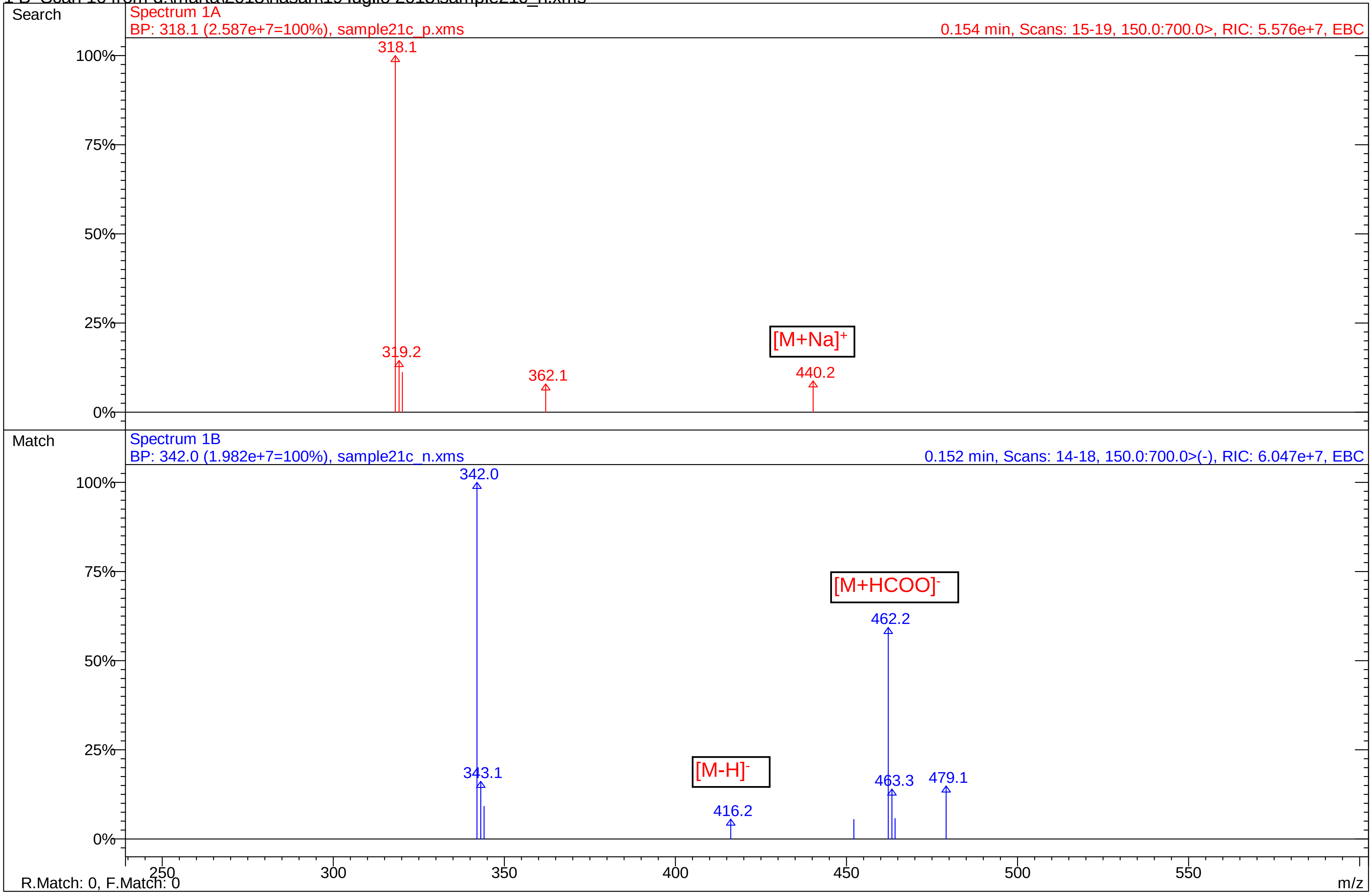

Print Date: 23 Jul 2018 12:12:07

Spectra Plots - 23/07/2018 12:12

1 A Scan 17 from d:\marta\2018\hasan\19 luglio 2018\sample22c_p.xms
1 B Scan 16 from d:\marta\2018\hasan\19 luglio 2018\sample22c_n.xms

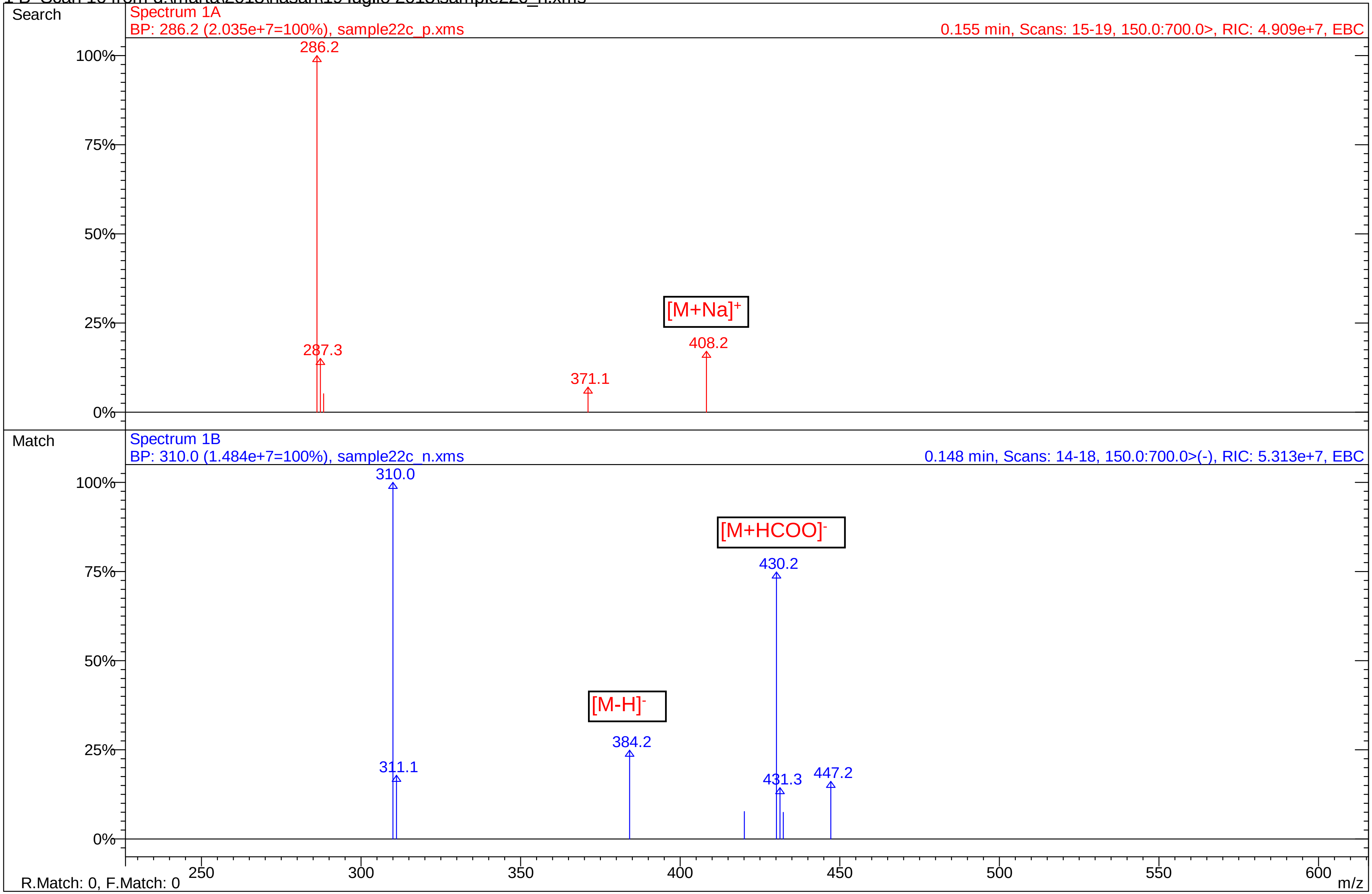

Print Date: 23 Jul 2018 12:13:22

Spectra Plots - 23/07/2018 12:13

1 A Scan 18 from d:\marta\2018\hasan\19 luglio 2018\sample23c_p.xms
1 B Scan 16 from d:\marta\2018\hasan\19 luglio 2018\sample23c_n.xms

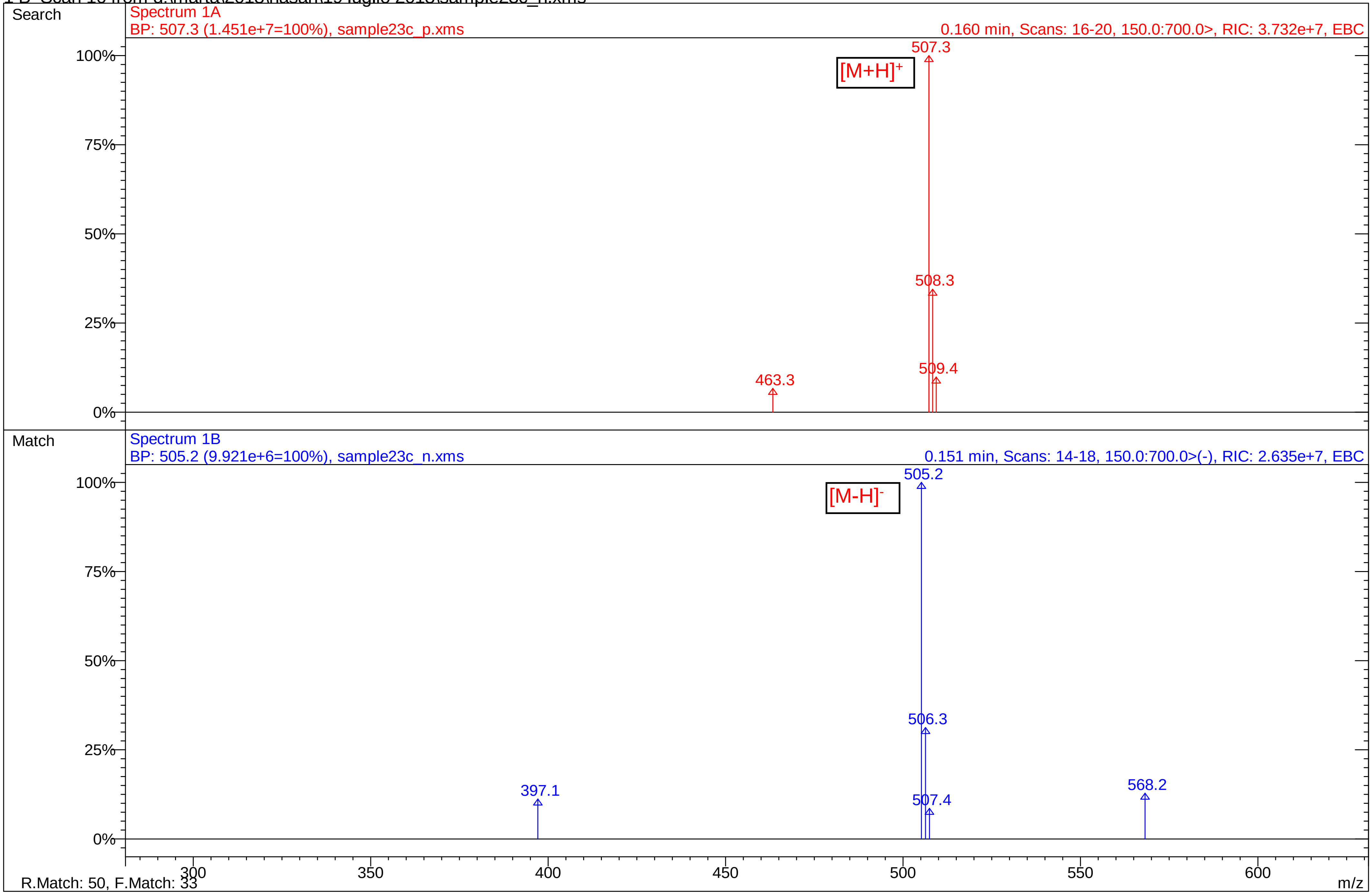

Print Date: 23 Jul 2018 12:19:11

Spectra Plots - 23/07/2018 12:19

1 A Scan 17 from d:\marta\2018\hasan\19 luglio 2018\sample24c_p.xms
1 B Scan 16 from d:\marta\2018\hasan\19 luglio 2018\sample24c_n.xms

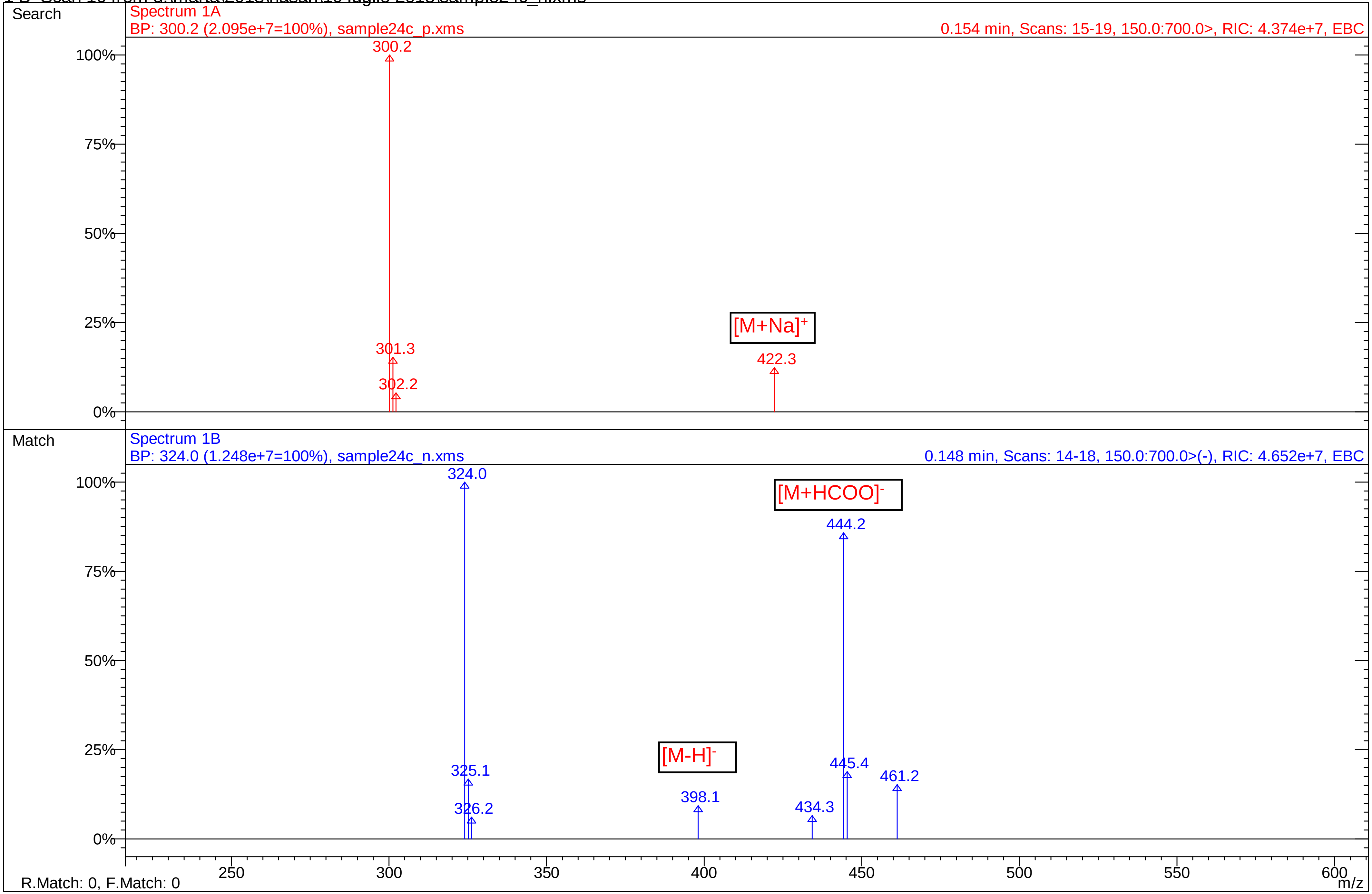

Supplement: Supplemental Material [file IENZ_A_1710503_SM6991.pdf]
